# *rm*Combi-OGAB for the Directed Evolution of a Biosynthetic Gene Cluster toward Productivity Improvement

**DOI:** 10.1021/acssynbio.4c00734

**Published:** 2025-02-05

**Authors:** Naoki Miyamoto, Kentaro Hayashi, Naohisa Ogata, Naoyuki Yamada, Kenji Tsuge

**Affiliations:** Synplogen Co., Ltd., Kobe, Hyogo 6500047, Japan

**Keywords:** rmCombi-OGAB, directed evolution, random mutagenesis, error-prone PCR, biosynthetic gene cluster, antibiotic

## Abstract

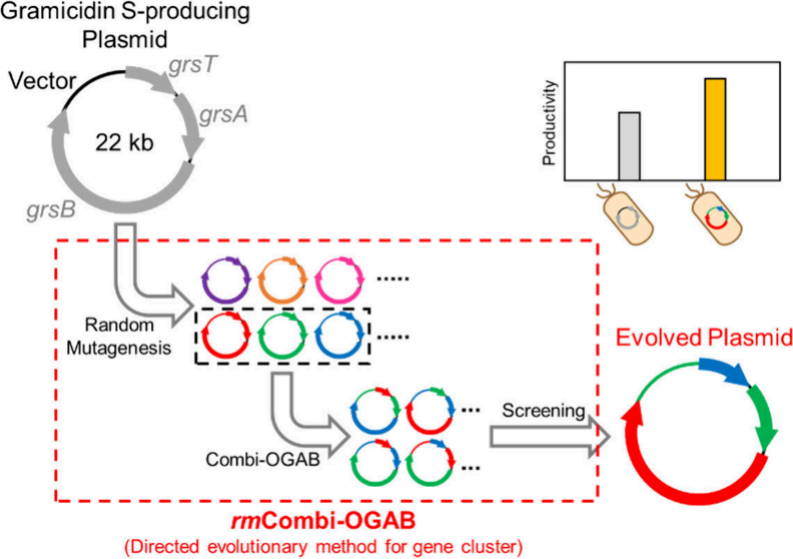

**Combi**natorial **O**rdered **G**ene **A**ssembly in ***B****acillus subtilis* (Combi-OGAB) enables construction of combinatorial libraries of various genetic elements, such as promoters in a biosynthetic gene cluster (BGC), and screening of highly productive combinations from the library. The combinations are limited by the library design, and the selectable productivity is defined within the combination. To refine the selected BGC using conventional Combi-OGAB with expanded diversity, we devised a directed evolutionary method called as **r**andom **m**utagenesis with **Combi-OGAB** (*rm*Combi-OGAB), which includes random mutagenesis by error-prone PCR and Combi-OGAB. In the present study, Gramicidin S (GS)-producing plasmids were used to examine the utility of *rm*Combi-OGAB. GS plasmids, originally generated using conventional Combi-OGAB, were successfully evolved using *rm*Combi-OGAB. *B. subtilis* carrying the evolved plasmid with unpredictable mutations showed a 1.5-fold improvement in the GS productivity. We thus expect that *rm*Combi-OGAB can be applied to various BGCs for useful products, such as antibiotics, to improve their productivity.

## Introduction

In synthetic biology, combinatorial libraries of various genetic elements (e.g., coding genes, promoters, and ribosome-binding sites) play a critical role in building novel biosynthetic gene clusters (BGCs) and optimizing the production of target products.^1,2^ The gene synthesis technology, OGAB (**O**rdered **G**ene **A**ssembly in ***B****acillus subtilis*),^3^ enables assembly of multiple DNA fragments to long and complex DNA sequences. Combi-OGAB (**Combi**natorial **OGAB**)^4^ is an application of OGAB, designed specifically for the effective construction of exhaustive combinatorial libraries to build BGC. Although the selected BGC from the combinatorial library can confer productivity to the host strain, the selectable productivity is defined within the designed library with limited combinations. Further, many genetic elements still remain uncharacterized, and genetic elements in the assembled gene cluster do not function as predicted.^5^ Therefore, the constructed BGCs may not perform perfectly. Directed evolution, which involves mutagenizing DNA sequences, analyzing the productivity of clones, selecting the best producer, and repeating these steps until productivity is maximized, can be utilized as a strategy to refine selected BGCs from the combinatorial library toward much higher productivity.^5^

In this Technical Note, we describe a directed evolutionary method, *rm*Combi-OGAB (**r**andom **m**utagenesis with **Combi-OGAB**), which includes random mutagenesis in BGC via epPCR (**e**rror-**p**rone **PCR**) for expansion to unlimited diversity, construction of a combinatorial library of mutated gene fragments, and screening for the more productive BGC with unpredictable mutations using Combi-OGAB. Based on the principles of OGAB and conventional Combi-OGAB, *rm*Combi-OGAB enables the mutagenization of long DNA (>10 kb), such as BGCs of microbial metabolites. Therefore, the whole BGC or several separate regions in the BGC can be mutagenized simultaneously for directed evolution.

In a previous report,^4^ we successfully created a productive *B. subtilis* for Gramicidin S (GS), an antibiotic peptide against Gram-positive bacteria including *B. subtilis* ([MIC] = 1.7 μM [= 1.9 μg/mL]),^6^ natively produced by *Aneurinibacillus migulanus*,^7^ using conventional Combi-OGAB. We constructed a combinatorial library for the GS BGC with growth-phase-dependent promoters^8^ of *B. subtilis* for each gene (*grsT*, *grsA*, and *grsB*), and mono-cistronically optimized the promoter combination to establish the GS producer. The created clone, 3^rd^-C2, showed a GS productivity of approximately 30 mg/L, which was 50-fold higher than that of *B. subtilis* carrying the native GS BGC plasmid. In this study, we aimed to demonstrate the utility of *rm*Combi-OGAB by evolving the GS BGC plasmids (22 kb) selected in a previous study toward a much higher GS productivity than that of 3^rd^-C2.

## Results and Discussion

**Figure 1:**
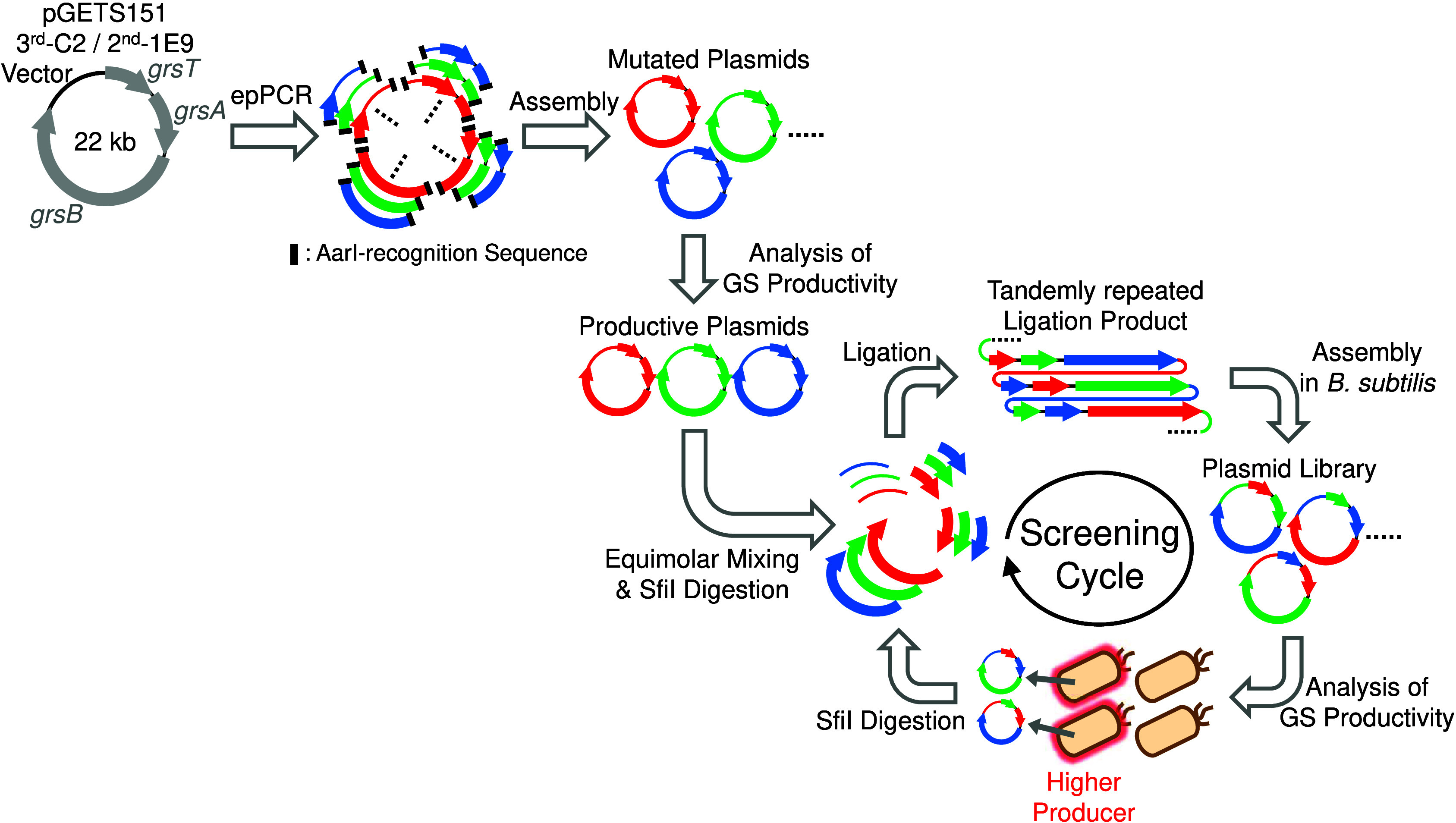
Schematic of the *rm*Combi-OGAB procedures in this study. Four fragments were amplified using epPCR from two template plasmids, pGETS151 3^rd^-C2 or 2^nd^-1E9, individually, and the fragments were assembled to construct mutated plasmids. The productive plasmids among mutated plasmids were equimolarly mixed and digested with SfiI. Digested DNA fragments were ligated to a tandemly repeated form to shuffle the mutated genes and vectors. *B. subtilis* was transformed with this ligation product, and the plasmid library with shuffled mutated fragments was assembled in *B. subtilis*. The GS productivity of each *B. subtilis* clone was analyzed, and the plasmids of some higher producers were mixed, and again digested with SfiI for the 2^nd^ cycle of library construction. These procedures were repeated until the productivity was saturated.

For mutagenesis, we selected epPCR, which can mutagenize the targeted sequences via the replication errors of DNA polymerase. Plasmids of 3^rd^-C2 and 2^nd^-1E9, which were screened using conventional Combi-OGAB in a previous study,^4^ were utilized as the templates for epPCR. A schematic of *rm*Combi-OGAB is shown in Figure 1.

The plasmids (approximately 22 kb) were divided into four fragments of approximately 5.5 kb using epPCR. Both ends of each fragment contained a recognition sequence for the restriction enzyme AarI, and the sticky ends generated by AarI-digestion were designed to define the ligation order. AarI-digested fragments were assembled into the mutated plasmids in *B. subtilis* BUSY9797 carrying pUB8.^9^ Plasmid pUB8 contains *lpa-8* coding 4′-phosphopantetheinyl transferase to activate the production mechanism of non-ribosomal peptides including GS.^9,10^ Therefore, the transformed *B. subtilis* clones were directly used for GS biosynthesis.

**Figure 2:**
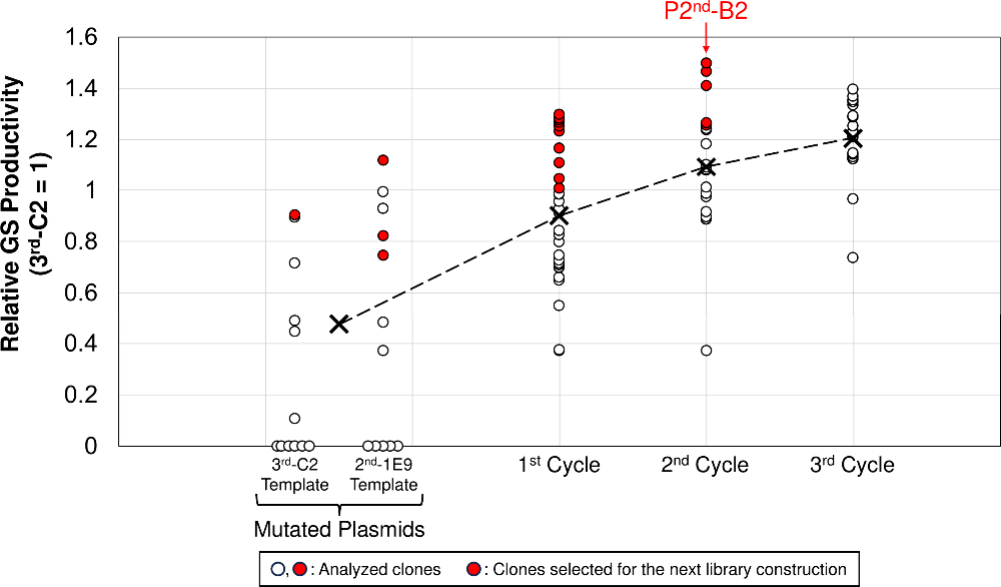
GS productivity monitoring in the screening cycles. The Mutated Plasmids, 1^st^ Cycle, 2^nd^ Cycle, and 3^rd^ Cycle included 24 (12 for 3^rd^-C2 template and 12 for 2^nd^-1E9 template), 30, 20, and 19 clones, respectively. The circles indicate analyzed clones, and the red circles indicate clones selected for the next library construction. The X-marks indicate the average productivity in each screening cycle, and the dashed line indicates the transition of average GS productivity between cycles.

Twelve transformants each from 3^rd^-C2 and 2^nd^-1E9 were randomly selected, and their GS productivity and mutation rates were analyzed. Six 3^rd^-C2 mutants and five 2^nd^-1E9 mutants showed no detectable GS production (Figure 2 and Figure S1), and their mutation rate was calculated as approximately 0.77 substitutions/kb based on that of all 24 clones. No deletions or insertions were observed. The highest producer among 3^rd^-C2 mutants, the highest producer among 2^nd^-1E9 mutants, and two 2^nd^-1E9 mutants that produced less GS-byproducts (with one or two ^L^Lys residues at the ^L^Orn positions in GS^11^) than those produced by 2^nd^-1E9, were selected as productive plasmids for the subsequent *rm*Combi-OGAB procedure.

An equimolar mixture of these four plasmids was digested into four fragments (three gene fragments and one vector fragment) using the restriction enzyme SfiI. The sticky ends generated by SfiI digestion were different in one plasmid molecule, and each sticky end at the same position between plasmid molecules was the same. Therefore, sticky ends could define the ligation order for the correct assembly of the GS BGC plasmid. The digested fragments were ligated to a tandemly repeated form, and BUSY9797 carrying pUB8 was transformed with the ligation product to construct the 1^st^ cycle-library. Thirty clones were randomly selected from approximately 7,000 transformants, and their GS productivity was analyzed. Except for clones carrying mutated plasmids, non-producers were not detected during the screening cycles. When GS biosynthesis and analysis of GS productivity were performed in each cycle, 3^rd^-C2 was also examined in every cycle as an internal standard. The top 10 producers were selected, and their plasmids were equimolarly mixed. For the 2^nd^ cycle-library, the plasmid mixture was again digested with SfiI, and the fragments were ligated and assembled to construct the 2^nd^ cycle-library in the same manner as for the 1^st^ cycle-library. Twenty clones were randomly selected from approximately 2,000 transformants, and the top five producers were used to construct the 3^rd^ cycle-library. In the 3^rd^ cycle, 19 clones were randomly selected from approximately 2,000 transformants and their GS productivity was analyzed. As none of the clones in the 3^rd^ cycle showed a productivity higher than that of the top producer in the 2^nd^ cycle, we finished the screening cycle. The highest producer was the B2 clone of the 2^nd^ cycle (**P2^nd^-B2**), and its productivity was approximately 1.50-fold higher than that of 3^rd^-C2.

Next, we conducted *rm*Combi-OGAB again (Figures S1 and S2). A plasmid mixture of the top 10 producers in the 2^nd^ cycle was used as the template for epPCR, and mutated plasmids (mutated plasmids (2^nd^)) were prepared using the procedures described above. Among the 12 analyzed clones, four were non-producers. The top four productive plasmids were used to construct the 1^st^ cycle (2^nd^)-library. Twenty-four clones were randomly selected from approximately 4,000 transformants, and their GS productivity was analyzed. The top 10 producers were then used to construct the 2^nd^ cycle (2^nd^)-library. Twenty-four clones were randomly selected from approximately 8,000 transformants, and their GS productivity was analyzed. None of the clones in the 2^nd^ cycle (2^nd^) showed a productivity higher than that of the top producer in the 1^st^ cycle (2^nd^), and the screening cycle was completed. The highest producer was the A8 clone of the 1^st^ cycle (2^nd^) (**PP1^st^-A8**), and its productivity was approximately 1.52-fold higher than that of 3^rd^-C2. The fold change was not significantly different from that of P2^nd^-B2. Therefore, the GS productivity in *B. subtilis* was suggested to have achieved a maximum owing to the toxicity of GS against *B. subtilis*.

Finally, the plasmid sequences of P2^nd^-B2 and PP1^st^-A8 were analyzed (Tables S1 and S2). In both plasmids, *P*_*mmgA*_ (2^nd^-1E9), *P*_*cdd*_ (3^rd^-C2), and *P*_*veg*_ (2^nd^-1E9) were selected as promoters for *grsT*, *grsA*, and *grsB*, respectively. P2^nd^-B2 possesses three substitutions in *grsA* (one synonymous and two non-synonymous substitutions), five substitutions in *grsB* (two synonymous and three non-synonymous substitutions), and four substitutions in the vector, whereas PP1^st^-A8 possesses two substitutions in *P*_*mmgA*_, two substitutions in *grsT* (two non-synonymous substitutions), two substitutions in *P*_*cdd*_, six substitutions in *grsA* (two synonymous and four non-synonymous substitutions), two substitutions in *P*_*veg*_, 16 substitutions in *grsB* (four synonymous and 12 non-synonymous substitutions), and six substitutions in the vector. These substitutions might enhance GS bioproduction in P2^nd^-B2 and PP1^st^-A8. We then analyzed the contribution of mutated *grsA*, mutated *grsB*, and/or the mutated vectors in P2^nd^-B2 to GS productivity. Six plasmids with a combination of mutated fragments and a mutation-free plasmid were constructed, and their GS productivity was analyzed (Figure S3). The plasmids containing a single mutated fragment (*grsA* mutant, *grsB* mutant, and vector mutant) showed lower GS productivity than that of P2^nd^-B2 and almost the same productivity as that of the mutation-free plasmid (No mutations). Plasmids containing two mutated fragments (*grsA* + *grsB* mutant, *grsA*+vector mutant, and *grsB*+vector mutant) showed higher GS productivity than that of the single mutant holders, and their average productivity was lower than that of P2^nd^-B2. These results suggest that all three mutated fragments were required for improved GS productivity in P2^nd^-B2, which was successfully realized by *rm*Combi-OGAB through the evolution of the whole 22 kb GS BGC plasmid.

epPCR has been utilized as a mutagenesis method to enhance the bioproduction, such as lycopene,^12^ C_40_ carotenoids,^13^ higher-chain alcohols,^14^ and pinene.^15^ These studies did not repeat the epPCR and screening processes. We examined whether *rm*Combi-OGAB is a more effective method to enhance productivity than repeated epPCR and screening. Repeating epPCR and screening did not generate better clones than those screened through *rm*Combi-OGAB (Figure S4). In this study, it took approximately 6 days/30 clones for GS biosynthesis, extraction, and HPLC analysis, which makes screening large numbers of clones challenging. Therefore, screening with Combi-OGAB is a realistic method. This suggests that *rm*Combi-OGAB, including mutagenesis via epPCR and combinatorial screening of mutated fragments via Combi-OGAB, has the potential to effectively enhance productivity when compared to repeating epPCR and screening.

In conclusion, we developed the *rm*Combi-OGAB method, which involves random mutagenesis in the BGC by epPCR with unlimited expansion of diversity, construction of a combinatorial library of mutated genes, and screening for a highly productive BGC with unpredictable mutations through Combi-OGAB. Here, *rm*Combi-OGAB is suggested as a desirable method for realizing the directed evolution of a productive BGC selected from a conventional Combi-OGAB library for much higher productivity. As described above, we successfully evolved previous GS producers toward 1.5-fold higher producers using *rm*Combi-OGAB. The 3^rd^-C2 BGC showed approximately 50-fold higher GS productivity than that of the native sequence in a previous study,^4^ while *rm*Combi-OGAB further showed approximately 1.5-fold productivity improvement. Therefore, the selected P2^nd^-B2 plasmid showed approximately 75-fold higher GS productivity than that of the native sequence. If the target antibiotic is nontoxic to the host or can be detoxified by the resistance system, we expect the BGC to evolve to a much higher productivity with *rm*Combi-OGAB. Therefore, *rm*Combi-OGAB can be applied to various gene clusters, which have not been targeted for directed evolution by any method, to establish higher producers, such as industrial strains.

## Methods

### Preparation of Mutated Plasmids

Four fragments were amplified from pGETS151 3^rd^-C2 (*P*_*sigW*_-*grsT*-*P*_*cdd*_-*grsA*-*P*_*lytR*_-*grsB*) and pGETS151 2^nd^-1E9 (*P*_*mmgA*_-*grsT*-*P*_*srfAA*_-*grsA*-*P*_*veg*_-*grsB*) as templates for epPCR. The 20 μL reaction mixture contained 14.3 μL of sterilized water, 2 μL of 10× Ex Taq buffer (Takara), 1.6 μL of 2.5 mM dNTPs mixture (Takara), 1 μL of primer mixture (each 3.2 μM), 0.1 μL of Ex Taq HS (Takara), and 1 μL of template (0.2 ng of total DNA). The primer sequences are listed in Table S3. The thermal cycling conditions were as follows: 96 °C for 2 min and 25 cycles of 98 °C for 10 s, 58 °C for 30 s, and 72 °C for 5 min 35 s. These conditions achieved approximately 0.7–0.8 substitutions/kb. The four epPCR products derived from 3^rd^-C2 or 2^nd^-1E9 were individually mixed, and the mixture was then digested with AarI. The AarI-recognition sequences in the epPCR products were designed to be removed via AarI-digestion, and the digested fragments were seamlessly ligated to the original GS plasmid form. The digested fragments were ligated to a tandemly repeated form in accordance with the generated sticky ends. *B. subtilis* BUSY9797 containing pUB8^9^ was transformed with tandemly repeated ligation products to construct mutated plasmids. Randomly selected transformants were transferred for analysis of their GS productivity, and their mutated plasmids were analyzed using a Miseq (Illumina) to determine the mutation rate.

### Construction of a Combinatorial Library of Mutated Fragments

Four productive plasmids were mixed in equimolar amounts and digested with the restriction enzyme SfiI. The digested DNA was purified and ligated into a tandem repeated form. The ligation product was used to transform *B. subtilis* BUSY9797 carrying pUB8. Transformants were plated on an LB plate containing 10 μg/mL tetracycline and 10 μg/mL kanamycin, and the plate was incubated overnight at 30 °C. Single colonies were individually picked and cultured in 300 μL of LB medium containing 10 μg/mL tetracycline and 10 μg/mL kanamycin in each well of a 96-well deep-well culture plate. The plate was incubated overnight with shaking (1,000 min^–1^) at 30 °C and then stored at −70 °C until GS productivity analysis.

### Combi-OGAB Screening Cycles

A small aliquot of the frozen stock was inoculated into 2 mL of YTG medium (50 g/L yeast extract, 50 g/L bacto tryptone, and 5 g/L glucose) containing 10 μg/mL tetracycline and 10 μg/mL kanamycin and cultured (200 min^–1^) at 30 °C for 72 h. GS was extracted from each culture with 2 mL using ethyl acetate, and the ethyl acetate fraction was evaporated to dryness. The residue was re-dissolved in 200 μL of 70% methanol containing 0.05% formic acid. The analytical HPLC conditions were the same as those described previously.^4^ The plasmids of the selected producers based on productivity were mixed, and the plasmid mixture was digested again with SfiI. The digested DNA was ligated for the next cycle. The subsequent procedures were performed as described above and repeated until the average GS productivity of the screening cycles was saturated.

## Supporting Information Available

**Figure S1**, GS productivity of each clone in all the screening cycles; **Figure S2**, GS productivity monitoring in the whole screening cycles; **Figure S3**, Analysis of the contribution of mutated gene fragments to GS productivity in P2^nd^-B2; **Figure S4**, GS productivity of 30 randomly selected clones after epPCR of the top 10 producers in the 1^st^ cycle; **Table S1**, Analyzed plasmid sequences of 3^rd^-C2, 2^nd^-1E9, P2^nd^-B2, and PP1^st^-A8, **Table S2**, Nucleotide and amino acid substitutions in pGETS151 P2^nd^-B2 and pGETS151 PP1^st^-A8; **Table S3**, List of primers used for epPCR (PDF)
