# Supplementary material for: rmCombi-OGAB for the Directed Evolution of a Biosynthetic Gene Cluster toward Productivity Improvement
Source: ACS Synth Biol. 2025 Feb 5;14(2):629–33. doi: 10.1021/acssynbio.4c00734 (PMC11852201; doi:10.1021/acssynbio.4c00734)
Supplement: Supplementary file 1 — sb4c00734_si_001.pdf [file sb4c00734_si_001.pdf]

Supporting information for

*rm*Combi-OGAB for the Directed Evolution of a Biosynthetic Gene Cluster toward Productivity Improvement

Naoki Miyamoto*, Kentaro Hayashi, Naohisa Ogata, Naoyuki Yamada, and Kenji Tsuge

Synplogen Co., Ltd., Kobe, Hyogo 6500047, Japan

E-mail: n_miyamoto@synplogen.com, orcid.org/0000-0001-5544-191X

Contents

Figure S1. GS productivity of each clone in all the screening cycles.

Figure S2. GS productivity monitoring in the whole screening cycles.

Figure S3. Analysis of the contribution of mutated gene fragments to GS productivity in P2nd-B2.

Figure S4. GS productivity of 30 randomly selected clones after epPCR of the top 10 producers at the 1st cycle.

Table S1. Analyzed plasmid sequences of the 3rd-C2, 2nd-1E9, P2nd-B2, and PP1st-A8.

Table S2. Nucleotide and amino acid substitutions in pGETS151 P2nd-B2 and pGETS151 PP1st-A8.

Table S3. List of primers used for epPCR.

Supplemental Figures

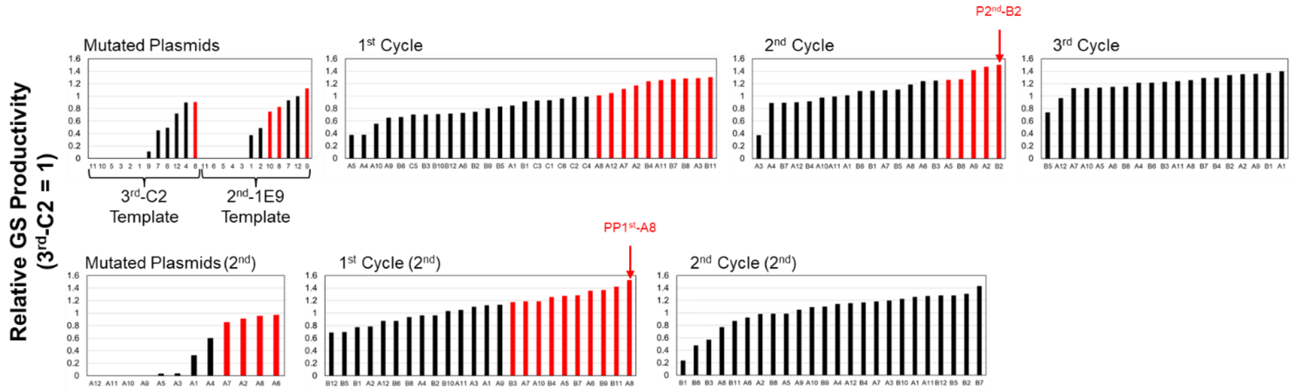

Figure S1. GS productivity of each clone in all the screening cycles. The X-axis indicates the names of the clones. Black bars indicate the GS productivity of each clone, and red bars indicate clones selected for the next library construction. The graph type is exchanged from that in Figure 2.

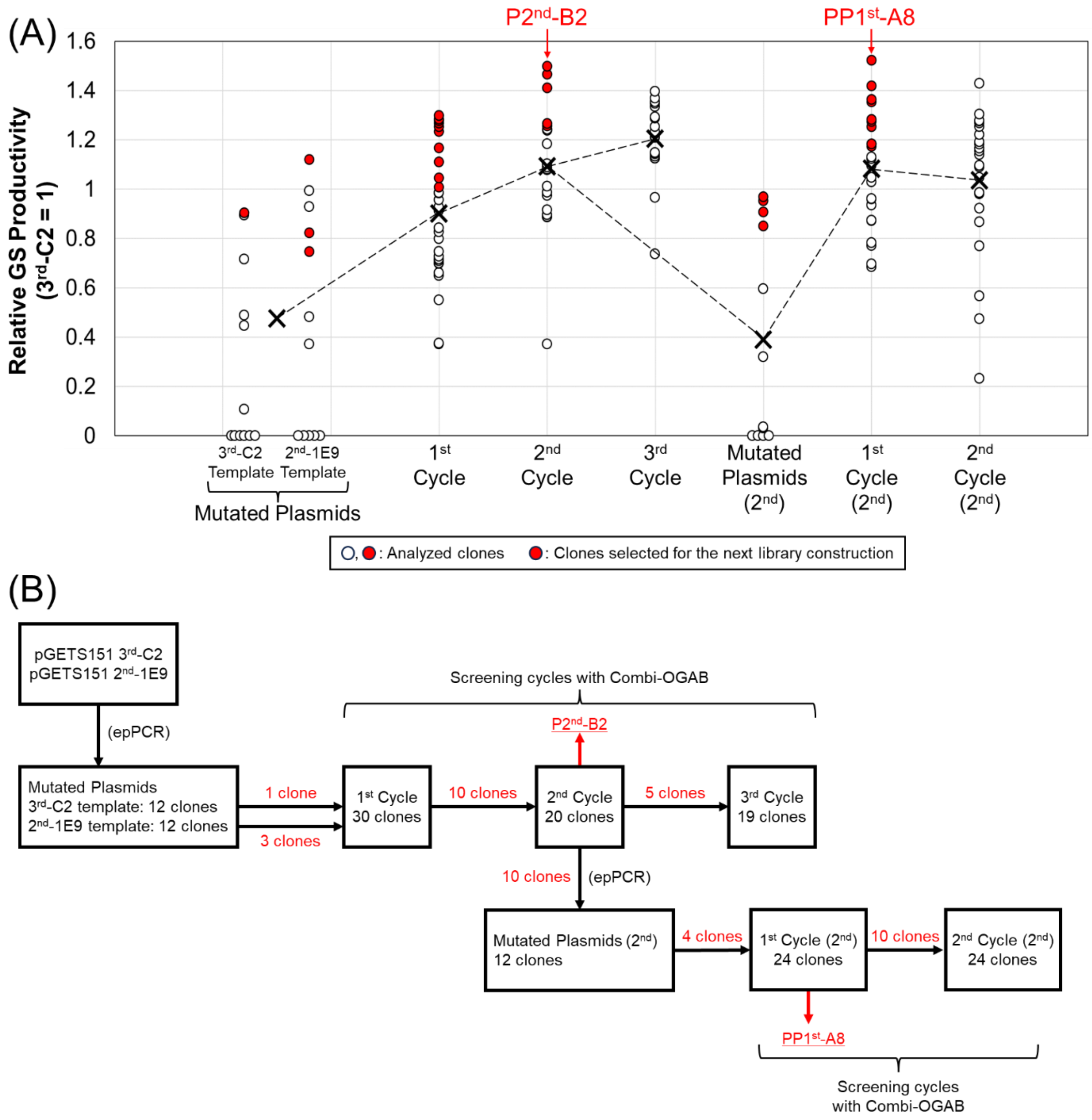

Figure S2. GS productivity monitoring in the whole screening cycles. (A) The Mutated Plasmids, 1st Cycle, 2nd Cycle, 3rd Cycle, Mutated Plasmids (2nd), 1st Cycle (2nd), and 2nd Cycle (2nd) included 24 (12 for 3rd-C2 template and 12 for 2nd-1E9 template), 30, 20, 19, 12, 24, and 24 clones, respectively. The circles indicate analyzed clones, and red circles indicate clones selected for the next library construction. The X-marks indicate average productivity in each screening cycle, and the dashed line indicates the transition of average GS productivity between cycles. (B) The overall *rm*Combi-OGAB scheme in this study. The description of each step is the same as that in (A). The black number indicates the number of analyzed clones (circles in (A)), and the red number indicates the number of clones selected for construction of the subsequent library (red circles in (A)).

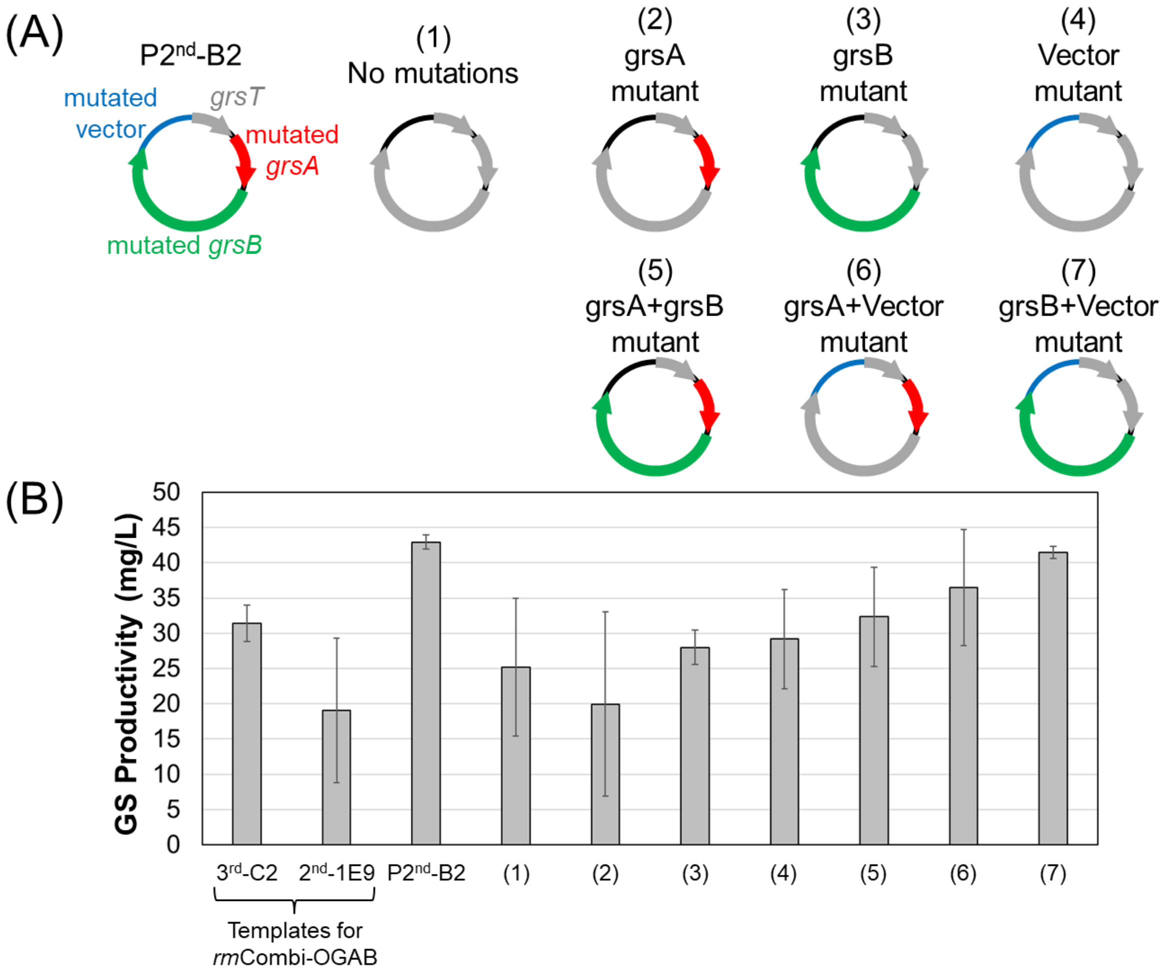

Figure S3. Analysis of the contribution of mutated gene fragments to GS productivity in P2nd-B2. (A) Images of the plasmids prepared for analyzing the contribution of mutated fragments to GS productivity. The gray arrows indicate non-mutated genes, and the black line indicates non-mutated vectors. The red arrow, green arrow, and blue line indicate mutated *grsA* (T-5,047-C, A-5,067-G, and T-5,972-A), mutated *grsB* (A-11,590-G, G-13,473-A, T-13,548-C, A-14,905-G, and A-20,013-T), and mutated vector (T-274-C, G-1,247-A, A-1,955-G, and T-3,077-C), respectively. Details of the mutations in P2nd-B2 are described in Table S2. Each mutated fragment, mutated *grsA*, mutated *grsB*, and mutated vector, was purified after SfiI digestion of pGETS151 P2nd-B2, and mutation-free fragments were purified from SfiI-digested pGETS151 3rd-C2 and 2nd-1E9. Then, seven plasmids, i.e., no mutations, *grsA* mutant, *grsB* mutant, vector mutant, *grsA*+*grsB* mutant, *grsA*+vector mutant, and *grsB*+vector mutant, were constructed by assembling the objective fragments. (B) GS productivity of *B. subtilis* carrying the constructed seven plasmids, 3rd-C2, 2nd-1E9, or P2nd-B2 (N=3). The Y-axis indicates GS productivity (mg/L), and the X-axis indicates clones of 3rd-C2, 2nd-1E9, and constructed plasmids described in (A). After transformation of *B. subtilis* carrying pUB8 with these 10 plasmids, respectively, single colonies were inoculated in 2 mL of LB containing 10 μg/mL tetracycline and 10 μg/mL kanamycin, and cultured overnight at 30°C. Each overnight culture was then stored at -70°C. Each strain was streaked onto an LB plate containing 10 μg/mL tetracycline and 10 μg/mL kanamycin from the frozen stocks. Three randomly-selected colonies were cultured overnight in 2 mL of LB containing 10 μg/mL tetracycline and 10 μg/mL kanamycin at 30°C. The 2 μL overnight culture was sub-cultured into 2 mL of YTG containing 10 μg/mL tetracycline and 10 μg/mL kanamycin and cultured overnight at 30°C. The A_{600} of the overnight culture was determined, and the aliquot with A_{600}=1 was inoculated into 2 mL of YTG containing 10 μg/mL tetracycline and 10 μg/mL kanamycin, followed by culturing at 30°C for 72 hours. The procedures used for the extraction and analysis of GS productivity are described in the methods section.

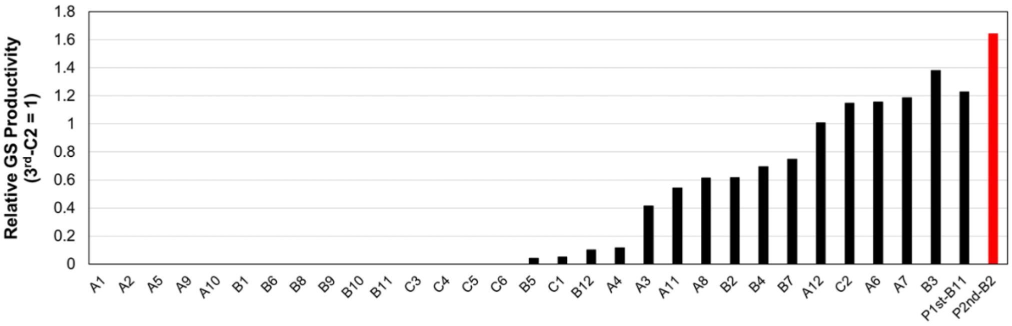

Figure S4. GS productivity of 30 randomly selected clones after epPCR of the top 10 producers at the 1st cycle. The X-axis indicates the names of the clones. Black bars represent the GS productivity of each clone, and the red bar indicates P2nd-B2 (the highest producer in first *rm*Combi-OGAB). To demonstrate the screening efficiency of *rm*Combi-OGAB, epPCR was again conducted using a mixture of the top 10 productive plasmids of the 1st cycle as the template. The transformation procedure was the same as that described in the manuscript. Thirty transformants were randomly selected, and their GS productivity was analyzed. The B11 clone of the 1st cycle (P1st-B11; the plasmid with the highest GS productivity in 1st cycle) and P2nd-B2 were also processed for GS biosynthesis, and productivity analyses were performed for comparison. Fifteen clones (A1, A2, A5, A9, A10, B1, B6, B8, B9, B10, B11, C3, C4, C5, and C6) did not show detectable production, and no plasmids with productivity higher than that of P2nd-B2 were obtained. This suggests that *rm*Combi-OGAB has potential to effectively screen productive plasmids when compared to repeating epPCR and screening without Combi-OGAB.

Supplementary Tables

Table S1. Analyzed plasmid sequences of the 3rd-C2, 2nd-1E9, P2nd-B2, and PP1st-A8 on next-generation sequencing (NGS). The red-colored and bold bases are substituted bases, and underlined bases are synonymous substitutions.

Plasmid Name	Sequence
pGETS151<br>3rd-C2	AAAAGGCCTTCTTGGCCGCCCTTCCCGGTCGATATGAACAGCTTATTTACATAATT<br>CACGTTATTGGTAGTTATAAATGAAATTCCTAATATCGGTTATGAAGTGAAATTGAA<br>TTTCTACTTGATCTTTCTCTCTATTTTTGTAAAATAAAATTAAGAATATTTAAATATT<br>CAATGATTCATTTTTGCAGAAATCGGAGGAAGAAGAATATATGAAAACATTTAAC<br>ATTTCTCAACAAGATCCCCCCATATTGTTGTATAAGTGATGAAATACTGAATTTAAA<br>ACCTAGTTTATATGTGGTAAAATGTTTTAATCAAGTTTAGGAGGAATTAATTATGAA<br>GTGTAATGAATAATGAGTGTAACAGGGTTCAATTAAAAGAGGGAAGCGTATCATT<br>AACCCTATAAACTACGTCTGCCCTCATTATTGGAGGGTGAAATGTGAATACATCCT<br>ATTCACAATCGAATTTACGACACAACCAAATTTTAATTTGGCTTTGCATTTTATCTT<br>TTTTTAGCGTATTAAATGAAATGGTTTTGAACGTGTCATTACCTGATATTGCAAATG<br>ATTTTAATAAACCACCAGCGAGTACAAACTGGGTGAACACAGCCTTTATGTTAAC<br>CTTTTCCATTGGAACAGCTGTATATGGAAAGCTATCTGATCAATTAGGCATCAAAA<br>GGTTACTCCTATTTGGAATTATAATAAATTGTTTCGGGTCGGTAATTGGGTTTGTTG<br>GCCATTCTTTCTTTTCCTTACTTATTATGGCTCGTTTTATTCAAGGGGCTGGTGCAG<br>CTGCATTTCCAGCACTCGTAATGGTTGTAGTTGCGCGCTATATTCCAAAGGAAAAT<br>AGGGGTAAAGCATTTGGTCTTATTGGATCGATAGTAGCCATGGGAGAAGGAGTCG<br>GTCCAGCGATTGGTGGAATGATAGCCCATTATATTCATTGGTCCTATCTTCTACTCA<br>TTCCTATGATAACAATTATCACTGTTCCGTTTCTTATGAAATTATTAAAGAAAGAAG<br>TAAGGATAAAAGGTCATTTTGATATCAAAGGAATTATACTAATGTCTGTAGGCATT<br>GTATTTTTTATGTTGTTTACAACATCATATAGCATTTCTTTTCTTATCGTTAGCGTGC<br>TGTCATTCCTGATATTTGTAAAACATATCAGGAAAGTAACAGATCCTTTTGTTGATC<br>CCGGATTAGGGAAAAATATACCTTTTATGATTGGAGTTCTTTGTGGGGGAATTATAT<br>TTGGAACAGTAGCAGGGTTTGTCTCTATGGTTCCTTATATGATGAAAGATGTTCAC<br>CAGCTAGGTACTGCCGAAATCGGAAGTGTAATTATTTTCCCTGGAACAATGAGTG<br>TCATTATTTTCGGCTACATTGGTGGGATACTTGTTGATAGAAGAGGTCCTTTATACG<br>TGTTAAACATCGGAGTTACATTTCTTTCTGTTAGCTTTTTAACTGCTTCCTTTCTTT<br>TAGAAACAACATCATGGTTCATGACAATTATAATCGTATTTGTTTTAGGTGGGCTTT<br>CGTTCACCAAAACAGTTATATCAACAATTGTTTCAAGTAGCTTGAAACAGCAGGA<br>AGCTGGTGCTGGAATGAGTTTGCTTAACTTTACCAGCTTTTTATCAGAGGGAACA<br>GGTATTGCAATTGTAGGTGGTTTATTATCCATACCCTTACTTGATCAAAGGTTGTTA<br>CCTATGGAAGTTGATCAGTCAACTTATCTGTATAGTAATTTGTTATTACTTTTTTCA<br>GGAATCATTGTCATTAGTTGGCTGGTTACCTTGAATGTATATAAACATTCTCAAAG<br>GGATTTCTAAATCGTTAAGGGATCAACTTTGGGAGAGAGTTCAAAATTGATCCTTT<br>TTTTATAACAGGAATTGGGCATCAAATAAAACGAAAGACTGGGCCTTTCGTTTTAT<br>CTGTTGTTTGTCGGTGAACGCTCTCCTGAGTAGGACAAGTCCGCCGGGAGCGGAT<br>TTGAACGTTGCGAAGCAACGGCCCGGAGGGTGGCGGGCAGGACGCCCGCCATA<br>AACTGCCAGGCATCAAATTAAGCAGAAGGCCATCCTGACGGATGGCCTTTTTGCG<br>TTTCTACAAACTCTTCCTGTCGTCATATCTACAATTCTACACAGCCCAGTCCAGAC<br>TATTGAATTGTATCACGGTTTTGATATCCTACCAATAACAAATTGATTGGAGGAATG<br>CAAAGTGAATAATGAACCAGTAAAACGTGGTAAGAAGAACAGATGGGAATTAAA<br>CCTACCTATAATGACTTATGTAGTAGCTGATGATTGGATTGATAAACTAGGACACG<br>AAACGTTTACTTTATGGTTGAGGTTCCATACTTGGGTAGATAGAGAAGATGAACTC<br>CGAGATTATGATCGCATACCTAGAAGTTTTGAGAACATATATAAAAAGACACTAGG<br>AATCTCAAAAAGTAAGTTTTATAGATTGATAAAACCTTTATGGGAATATGGATTAAT<br>AGACATCATAGAATACGAAGAATCTAACCGTAATTCTACTAAACCTAAAAATATAA<br>TTGTTTATGAGTATCCTTTACACGAAATAGAAAGAAAGTATAAACCACTAGAAAA<br>ATTAAGAGATTGGGATAAAGACTATAATTCCGTTTCTAAAGAATTAGGTAAAACAG<br>GTGGTAGACCAAGGAAAAAAGATAGTGAAGAAGAACCCGAAAAGAAACCCGAA<br>GAAGTAACTAAAAAGAAACGTAAATATAAGTTAAAAAGAGTTATCCACAACGGTT

	TCAAAAATGAAACGGTGGAGGGTTTCAAAAATGAAACGGTGGAGGGTTTCAAA<br>AATGAAACGGTGACCGTTTCAAAAATAAAACCCAATAATTATTCAAATATCTTTAA<br>TAACTTATCAAATATTTCTACTAATGTTTCAAATAATTTATTAATTGATGATGATGAG<br>GAAATCGAAAATGAACCAACTGGTCGTACAATAAATAGGTCATTACTTTTTTCGCA<br>AGAAGATATTAAACAGGCCTATCAATTTATTAATAGATTTTCAGTTATACAGTTACG<br>TGAAAACTTTAGCTTTGATAAACACTTTGAAGAACGGTTGGTATGTTATTTATGGA<br>AAGCAGGGATTTCTACTTTTTACACGCACGAAATCAGTAAAATGATAAAAAAAAT<br>AGCAGACTATGAAAAATCTAAAAAAGGTAGATTAAACCCAATACGTGACCGAGC<br>CTTATATATGGTAAATGGTCTTGTAATGAATAGAGCTTCTTCCCAAAGTGAACATG<br>CTACTTATAAACTAAACCAATATAAAAAACAGAAGGAACAGGAAAAACAACAAC<br>AGGAGCAACAAAGATCAAGAGTACCGTTCTATAATTGGTTGGAGGAAAGAGAAG<br>AACAAACCGAAGGTCAACTACCCACCACTTAAGCGGCCGCAAGCTTGAAGAGCT<br>CTTCTTTCAGAACGCTCGGTTGCCGCCGGGCGTTTTTTATGAGATGTCTCGGCCTG<br>TTTGGCCATTAATCGAAGAAGAAGTGTGAAAAAGCGCAGCTGAAATAGCTGCGC<br>TTTTTTGTGTCATAATCCTTGCCCCCCTCCACCATTATTGGGCTATAGCCAAGCGGT<br>AAGGCAACGGACTTTGACTCCGTCATGCGTTGGTTCGAATCCAGCTAGCCCAGTC<br>ACAGACACCTTTGATCAAAAGGTGTCTTTTTTCTTTTCGGAAAAATCATTCCAACT<br>TCTAACTGTTCAGTCTGTATAATAATTTTAAAAATATGTTAAGGTAGTTTATTCACG<br>AATTACCATCTACACCCTGCCAAAAATTTGATAAACTTATTTTATAAAAAAATTGA<br>AACCTTTTGAAACGAAGCTCGTATACATACAGACCGGTGGAAGAATAAAGGGAG<br>AGGTGAATGTGACTTTTATTTCACAAGTAAATAAATGGTTTGTTAATGCTAATGTTA<br>ACTCAGCTGCAAAGCTTAGGCTATTCTGTATTCCATATGCAGGCGGTGGTGCTTCC<br>GCCTTTTATGAATGGAGTCATTTTTTTCCAAAGGAAATTGAAGTTTGTTCAATTCA<br>ATTACCTGGAAGGGAAAATAGGGGGGCGGAAGTTCCGCTAACAAATTTACAACA<br>GATAGTAGAAATAGTAGCTGAGGAAATACAACCATTAATAAATATTCCATTTGCTTT<br>TTTGGGGCATAGCATGGGAGCATTAATAAGTTTTGAACTGGCTCGCACAATACGG<br>CAAAAGAGTAATGTTAATCCGGTTCACTTGTTTGTTTCAGGGCGACATGCACCTC<br>AAATCCCATGTGCAAAACAAGACTATCATTTACTTCCCGATGAACAATTTATACAA<br>GAATTGCGTTCATTGAATGGAACTCCAGAGATAGTATTACAAGACGCAGAGATGA<br>TGAGTATATTACTCCCAAGACTTCGGGCTGATTTTTCTGTGTGTGGCTCCTATCAG<br>TACAAAAACGACGAGCCTTTTGAATGCCCAATCACTGCTTTTGGAGGAAAAAATG<br>ATAATGGTGTTACTTATCAATCATTAGAAGCCTGGAGAGAGCAAACCAAGAGGGA<br>ATTTTCTGTGTGTATGTATCCAGGTGATCATTTTTTTCTTTACGAAAGCAAATATGA<br>AATGATTGAGTTCATGTGTAAACAATTACGTTTAGTATTAGCTCCTAAAATATAAGG<br>CCTTGATGGCCATCGAAGAAGAAGTGTGAAAAAGCGCAGCTGAAATAGCTGCGC<br>TTTTTTGTGTCATAATCCTTATTGAGTGGATGATTATATTCCTTTTGATAGGTGGTAT<br>GTTTTCGCTTGAACTTTTAAATACAGCCATTGAACATACGGTTGATTTAATAACTG<br>ACAAACATCACCCTCTTGCTAAAGCGGCCAAGGACGCTGCCGCCGGGGCTGTTT<br>GCGTTTTTGCCGTGATTTCGTGTATCATTGGTTTACTTATTTTTTTGCCAAAGCTGT<br>AATGGCTGAAAATTCTTACATTTATTTTACATTTTTAGAAATGGGCGTGAAAAAAA<br>GCGCGCGATTATGTAAAATATAAAGTGATAGCGGTACCGGAACTATTTTTTACAGG<br>GGGTATATATGTTAAACAGTTCTAAAAGTATATTGATTCATGCTCAAAATAAAAATG<br>GAACGCATGAAGAGGAGCAGTATCTCTTTGCTGTGAACAACACCAAAGCGGAGT<br>ATCCACGTGATAAGACGATCCATCAGTTATTTGAGGAGCAGGTTAGTAAGAGGCC<br>AAACAATGTAGCCATTGTATGTGAAAATGAGCAACTTACCTACCATGAGCTTAATG<br>TGAAAGCCAATCAACTAGCACGGATTTTTATAGAAAAAGGGATTGGAAAAGACA<br>CTCTTGTTGGAATTATGATGGAGAAATCTATCGATTTATTTATAGGCATATTAGCCG<br>TTTTAAAAGCTGGTGGAGCATATGTTCCGATTGATATTGAATATCCTAAGGAAAGA<br>ATTCAATATATTCTTGATGATAGTCAGGCAAGAATGCTACTTACCCAGAAGCATTT<br>GGTTCATTTAATTCATAATATTCAATTTAATGGGCAAGTGGAAATTTTTGAAGAAG<br>ATACTATCAAAATTAGAGAAGGAACTAATCTACATGTACCAAGTAAATCAACCGAT<br>CTTGCTTATGTTATTTATACTTCTGGTACAACAGGCAATCCAAAAGGTACAATGCT<br>GGAGCATAAAGGAATAAGTAATCTAAAGGTATTTTTCGAAAATAGTCTTAACGTG<br>ACTGAAAAGGATAGAATTGGTCAATTTGCCAGCATCTCTTTTGATGCATCTGTATG<br>GGAGATGTTTATGGCTTTGTTAACGGGGGCTAGCCTGTATATTATCCTGAAGGATA<br>CAATCAATGATTTTGTGAAGTTTGAACAATACATTAACCAAAAGGAAATCACTGT<br>TATTACGTTACCACCTACCTATGTAGTTCATCTTGATCCAGAACGTATTTTATCGATA

	CAAACGTTAATTACAGCAGGCTCAGCTACCTCGCCTTCCTTAGTAAACAAGTGGA<br>AGGAGAAAGTAACTTACATAAATGCCTATGGCCCTACGGAAACAACTATTTGTGC<br>GACTACATGGGTAGCCACCAAAGAAACAATAGGTCATTCAGTTCCAATCGGAGCA<br>CCAATTCAAAATACACAAATTTATATTGTCGATGAAAATCTTCAATTAAAATCGGTT<br>GGTGAAGCTGGTGAATTGTGTATTGGTGGAGAAGGGTTAGCAAGGGGATATTGGA<br>AGCGACCGGAATTAACTTCCCAGAAGTTCGTTGATAACCCGTTTGTTCCAGGAGA<br>GAAGTTGTATAAAACAGGAGATCAGGCAAGATGGCTATCTGATGGAAATATTGAA<br>TATCTCGGAAGAATAGATAACCAGGTAAAGATTAGAGGTCACCGAGTTGAACTAG<br>AAGAAGTTGAGTCTATTCTTCTAAAGCATATGTATATTAGCGAAACTGCAGTAAGT<br>GTGCATAAAGATCACCAAGAACAGCCGTATTTGTGCGCTTATTTTGTATCGGAAAA<br>GCATATACCACTAGAACAGTTAAGACAATTCTCATCAGAAGAACTGCCAACGTAT<br>ATGATCCCTTCTTATTTTATCCAGTTAGACAAAATGCCGCTTACATCAAATGGGAA<br>GATTGATCGAAAGCAGTTGCCGGAACCTGATTTAACTTTCGGGATGAGGGTAGAC<br>TATGAAGCGCCGCGAAATGAAATCGAGGAAACGCTTGTTACTATCTGGCAGGATG<br>TATTAGGTATTGAGAAAATCGGTATTAAAGATAATTTCTATGCATTAGGTGGAGATT<br>CTATTAAAGCAATACAGGTTGCTGCTCGCCTGCATTCCTACCAATTAAAGCTAGAA<br>ACAAAAGATTTATTAAAGTATCCAACAATCGATCAACTCGTTCATTATATAAAAGA<br>TAGTAAAAGAAGAAGTGAGCAAGGTATTGTGGAAGGTGAGATTGGACTTACACC<br>TATTCAGCATTGGTTCTTTGAACAACAATTTACAAATATGCACCATTGGAACCAAT<br>CGTATATGTTGTATAGACCAAATGGGTTTGATAAAGAGATCTTGCTAAGGGTATTTA<br>ATAAAATTGTTGAGCATCATGATGCATTACGTATGATATACAAACATCATAACGGAA<br>AGATCGTGCAGATAAATCGGGGGCTTGAAGGTACGTTGTTTGATTTTTATACCTTT<br>GATTTAACTGCAAATGATAATGAGCAACAGGTGATTTGTGAAGAATCTGCTCGATT<br>ACAAAATAGTATAAACTTGGAAGTAGGCCCTCTAGTAAAGATAGCGCTGTTTCATA<br>CTCAGAATGGAGATCACCTGTTTATGGCTATTCATCATTTGGTTGTGGATGGTATTT<br>CTTGGAGGATTTTGTTTGAGGATTTGGCCACAGCTTATGAACAAGCAATGCATCA<br>GCAAACGATTGCTTTACCAGAGAAAACAGATTCATTTAAGGACTGGTCTATTGAA<br>TTAGAAAAATATGCGAACAGCGAATTATTCCTAGAAGAAGCTGAATATTGGCATC<br>ATTTGAATTATTATACCGAGAACGTTCAAATTAAGAAAGATTATGTCACCATGAAC<br>AATAAACAAAAGAATATACGTTATGTAGGAATGGAGTTAACAATAGAAGAGACAG<br>AAAAATTATTGAAAAATGTAAATAAAGCGTATCGAACAGAAATTAATGATATTTTA<br>TTAACGGCACTTGGCTTTGCACTCAAAGAATGGGCCGATATTGATAAAATTGTAAT<br>TAACTTAGAGGGACACGGACGGGAAGAAATACTGGAACAGATGAACATTGCAAG<br>GACGGTAGGCTGGTTTACTTCCCAGTATCCTGTTGTACTTGATATGCAAAAATCGG<br>ATGATTTGTCTTATCAAATCAAATTAATGAAAGAAAATTTACGCAGAATACCTAAC<br>AAAGGAATCGGATATGAAATTTTTAAGTATTTAACAACTGAATATTTACGGCCTGT<br>TTTACCCTTTACATTAAAGCCGGAAATTAACTTTAACTACTTAGGACAGTTCGATA<br>CGGACGTGAAAACTGAATTGTTTACTCGTTCTCCTTATAGCATGGGTAATTCATTA<br>GGACCAGATGGAAAAAATAATTTAAGCCCAGAAGGGGAAAGTTATTTTGTACTCA<br>ATATTAATGGTTTTATTGAAGAAGGTAAGCTTCACATCACCTTTTCTTATAATGAAC<br>AGCAGTATAAGGAGGATACCATTCAGCAATTGAGCCGGAGCTATAAGCAACATCT<br>TTTGGCCATCATTGAACATTGTGTACAGAAGGAAGATACTGAGTTAACTCCAAGT<br>GATTTCAGTTTCAAGGAACTTGAATTAGAAGAGATGGATGATATTTTCGATTTGTT<br>GGCCGATTCATTAACGTAAGGCCTCGATGGCCATCGAAGAAGAAGTGTGAAAAA<br>GCGCAGCTGAAATAGCTGCGCTTTTTTGTGTCATAATCCTCTAACCCTACATAAGT<br>ACCTTCTTTTGTTTCAATGTTACTGTCTGGCGATACATCTTCACCTTGACTCTTTTG<br>ACTATTAACCCCGCAACCCGAAAGAAGCAATATAAAGAACAGTAAAGCAATAAAT<br>TTTTTCATTTTTTTCACCTCATTATATTTTATCGTCAACCTATTTTATATTTTAAAGAA<br>AAATTAAGAAACAATGAAACTTTTTTTTATAAAAAACGACTATTTTAGGATTTCAT<br>TCTTGTATTAAATAGAGTTGTATTTATTGGAAATTTAACTCATAATGAAAGTAATTT<br>GGAACATTATTATGAGGTGCTAGCATGAGTACATTTAAAAAAGAACATGTTCAGG<br>ATATGTATCGTTTATCTCCCATGCAGGAAGGCATGTTGTTTCACGCATTACTTGATA<br>AAGATAAAAATGCTCACCTGGTACAAATGTCTATCGCGATCGAAGGTATCGTGGAT<br>GTGGAGCTGCTTAGTGAAAGCTTGAACATATTGATTGATAGATACGATGTGTTTAG<br>AACAACATTCTTACATGAAAAAATTAAACAACCGCTTCAGGTAGTGCTAAAGGAA<br>CGGCCTGTTCAGCTTCAATTTAAAGACATATCATCCTTAGATGAAGAAAAAAGAG<br>AACAGGCTATTGAGCAGTATAAGTATCAAGATGGGGAAACAGTCTTTGATTTAAC

	AAGAGATCCCTTGATGAGAGTAGCTATTTTTCAAACTGGTAAGGTTAACTACCAA<br>ATGATCTGGAGCTTCCACCATATTTTAATGGATGGTTGGTGCTTCAACATTATATTT<br>AATGACTTGTTCAATATATATCTGTCATTAAAAGAGAAGAAACCTCTTCAGTTAGA<br>GGCGGTGCAACCATATAAGCAGTTTATTAAGTGGCTTGAAAAACAAGATAAACAG<br>GAAGCACTTCGCTACTGGAAAGAACATTTAATGAATTATGATCAATCAGTAACATT<br>ACCTAAAAAGAAAGCAGCTATTAATAATACTACATATGAACCAGCACAGTTTCGTT<br>TTGCGTTTGACAAAGTGCTTACCCAGCAGCTGCTTCGTATTGCCAATCAAAGCCA<br>AGTAACACTAAATATTGTTTTTCAAACAATATGGGGGATTGTACTTCAAAAATACA<br>ATTCCACTAATGATGTTGTATATGGCTCTGTTGTATCAGGCCGTCCTTCTGAAATAT<br>CGGGAATTGAGAAAATGGTTGGACTATTTATTAATACTCTTCCATTACGTATCCAA<br>ACGCAAAAAGATCAATCATTTATTGAATTAGTAAAGACTGTTCATCAAAACGTCCT<br>TTTCTCGCAACAGCATGAGTATTTTCCATTGTATGAAATACAAAATCATACAGAAT<br>TAAAACAGAATCTGATTGATCATATTATGGTAATTGAAAATTATCCTTTAGTAGAAG<br>AATTGCAAAAGAATAGTATCATGCAAAAAGTAGGGTTTACAGTTCGTGATGTCAA<br>AATGTTTGAACCAACTAATTATGATATGACAGTTATGGTTTTACCTCGTGATGAAAT<br>TAGTGTCCGACTCGATTATAACGCAGCCGTTTATGATATAGATTTCATAAAAAAAAT<br>TGAAGGTCACATGAAAGAAGTGGCTTTATGCGTGGCAAATAATCCACATGTGTTA<br>GTACAGGACGTTCCTCTGCTTACAAAGCAAGAAAAACAACATTTATTGGTAGAGC<br>TGCATGATTCGATAACAGAGTATCCTGATAAGACGATTCATCAGTTATTTACAGAA<br>CAGGTAGAAAAAACACCAGAGCATGTGGCAGTTGTATTCGAAGATGAGAAAGTG<br>ACCTATAGAGAGCTGCATGAGAGATCTAATCAATTAGCCAGATTCTTAAGAGAAA<br>AAGGCGTAAAAAAAGAAAGCATCATAGGCATTATGATGGAGCGTTCAGTTGAAAT<br>GATTGTTGGGATCTTAGGGATTTTAAAAGCTGGTGGAGCTTTTGTGCCTATTGATC<br>CTGAATATCCAAAAGAAAGAATCGGCTATATGTTAGATTCTGTACGGCTAGTACTT<br>ACACAACGCCATTTAAAGGATAAATTTGCTTTTACGAAAGAAACGATAGTAATTG<br>AAGATCCAAGTATTTCACACGAGTTAACTGAAGAAATAGATTATATTAATGAATCA<br>GAGGACTTGTTTTATATTATTTATACATCAGGAACAACAGGTAAACCAAAAGGGGT<br>TATGCTAGAGCACAAAAACATCGTTAATCTGCTTCATTTTACTTTCGAGAAAACAA<br>ATATCAACTTTAGTGACAAAGTATTACAGTATACAACATGCAGTTTTGACGTGTGT<br>TACCAAGAAATTTTTTCGACGCTCTTGTCTGGAGGGCAATTATATCTTATTAGGAA<br>AGAAACTCAACGCGATGTAGAGCAATTATTTGATTTAGTAAAACGTGAAAATATT<br>GAAGTATTATCCTTTCCTGTGGCTTTTCTAAAATTTATTTTCAATGAAAGAGAATTT<br>ATCAATCGTTTTCCAACTTGCGTGAAACATATTATCACAGCAGGAGAACAATTAGT<br>AGTTAACAATGAGTTTAAACGTTATTTGCATGAACATAACGTACATTTACACAATC<br>ATTATGGTCCATCAGAAACGCATGTTGTTACCACCTATACTATTAATCCTGAAGCTG<br>AAATTCCTGAATTACCACCGATAGGAAAACCTATCTCCAATACATGGATTTATATTT<br>TGGATCAAGAACAACAACTACAACCACAAGGAATTGTAGGAGAGTTATATATTTC<br>GGGCGCAAATGTTGGAAGAGGATATTTGAATAATCAAGAATTAACGGCAGAAAA<br>ATTCTTTGCAGATCCCTTTAGGCCAAACGAACGGATGTACCGAACAGGGGATTTA<br>GCAAGGTGGTTGCCAGACGGAAATATCGAATTTTTAGGAAGGGCCGATCATCAGG<br>TGAAAATTAGGGGGCATCGAATAGAGCTTGGTGAGATCGAGGCACAATTATTAAA<br>TTGTAAGGGTGTAAAAGAAGCTGTTGTTATCGATAAAGCGGATGATAAAGGCGGA<br>AAATATTTATGTGCCTATGTTGTTATGGAAGTAGAAGTAAATGACTCTGAGCTTCG<br>AGAATATTTGGGGAAAGCTTTGCCTGATTATATGATCCCGTCGTTCTTTGTTCCGTT<br>GGATCAGCTGCCGCTTACACCAAACGGAAAAATAGACAGAAAATCTCTTCCGAAT<br>CTAGAGGGGATTGTGAATACAAACGCAAAATATGTAGTACCTACAAATGAGCTGG<br>AAGAAAAATTGGCTAAAATCTGGGAAGAAGTACTTGGGATTTCTCAGATCGGTAT<br>ACAAGACAATTTCTTTTCGTTAGGCGGGCATTCTCTTAAAGCCATTACGCTTATTT<br>CCCGTATGAACAAAGAGTGTAATGTAGACATTCCTCTACGTTTGTTATTTGAAGCA<br>CCAACCATTCAGGAAATCTCTAATTATATAAACGGGGCAAAGAAAGAAAGCTATG<br>TTGCCATTCAGCCTGTACCAGAACAAGAGTACTATCCTGTATCATCAGTTCAAAAA<br>AGAATGTTTATTCTTAATGAATTTGATCGTTCAGGTACGGCCTATAATTTACCTGGT<br>GTTATGTTTCTAGATGGAAAATTGAACTACCGACAATTGGAAGCAGCGGTAAAAA<br>AATTAGTTGAGCGACATGAAGCGCTGCGTACTTCCTTTCATTCAATTAATGGGGAA<br>CCAGTTCAGCGGGTGCATCAAAATGTAGAACTGCAGATTGCTTATTCAGAGTCAA<br>CGGAAGATCAGGTGGAGCGAATTATTGCGGAATTTATGCAACCATTTGCTCTTGA<br>AGTTGCTCCGTTACTTCGTGTAGGTCTTGTTAAATTGGAGGCAGAACGTCATCTAT

	TTATAATGGATATGCATCATATCATCTCGGATGGGGTATCCATGCAGATCATGATTC<br>AAGAAATTGCTGATTTGTATAAAGAAAAGGAACTTCCTACGTTAGGCATTCAATAT<br>AAAGACTTTACTGTTTGGCATAATCGCTTGCTTCAATCGGATGTTATTGAAAAACA<br>AGAAGCTTACTGGCTGAACGTATTTGCAGAAGAGATTCCAGTATTGAATCTACCG<br>ACCGATTACCCAAGACCAACCATTCAAAGCTTTGATGGTAAAAGATTTACATTCA<br>GTACAGGAAAGCAGCTTATGGATGATTTATACAAGGTGGCAACAGAAACAGGAA<br>CAACACTATATATGGTTTTACTTGCTGCGTATAATGTTTTCTTATCGAAGTATTCCGG<br>GCAAGATGACATCGTTGTAGGAACACCGATTGCTGGTAGGTCCCATGCTGATGTG<br>GAAAATATGCTGGGGATGTTTGTAAATACATTAGCAATAAGAAGTCGTTTAAATAA<br>TGAGGATACTTTTAAAGATTTTTTAGCAAATGTAAAACAAACGGCTTTGCATGCCT<br>ATGAAAATCCAGATTACCCATTTGATACGCTTGTCGAAAAGTTGGGTATACAGAG<br>AGATTTAAGTAGAAATCCATTATTTGATACGATGTTTGTTTTGCAAAATACGGATAG<br>AAAGTCTTTTGAGGTTGAACAGATAACGATTACACCATATGTTCCAAATAGCAGA<br>CATTCTAAATTTGATCTTACATTAGAGGTTAGCGAAGAACAAAATGAGATTTTATT<br>ATGCCTAGAATATTGCACTAAATTATTTACGGATAAAACAGTTGAAAGAATGGCTG<br>GTCATTTTTTACAGATCTTGCATGCAATTGTTGGGAACCCAACGATTATAATATCAG<br>AAATCGAGATATTGTCTGAAGAAGAAAAACAACATATTTTATTCGAGTTCAACGA<br>TACGAAAACCACATATCCACATATGCAAACAATTCAAGGATTATTTGAGGAACAG<br>GTGGAGAAAACGCCCGACCATGTTGCAGTTGGATGGAAAGACCAAACATTAACG<br>TATCGGGAACTTAACGAAAGAGCGAATCAGGTCGCAAGAGTCTTACGGCAAAAA<br>GGAGTCCAACCCGATAATATCGTGGGATTGCTGGTTGAGCGTTCACCTGAAATGC<br>TCGTGGGTATCATGGGAATTCTTAAAGCAGGGGGAGCTTATTTACCTCTTGATCCG<br>GAGTACCCAGCGGATAGAATTTCGTACATGATACAAGATTGTGGTGTACGCATTAT<br>GCTTACCCAACAGCATCTTTTATCTTTAGTACATGATGAATTTGATTGTGTTATTTT<br>GGATGAGGACAGTTTGTACAAGGGGGATTCTTCCAATTTGGCTCCGGTTAACCAG<br>GCCGGGGATTTAGCCTACATCATGTACACTTCTGGTTCTACAGGAAAGCCTAAAG<br>GTGTTATGGTAGAACATCGAAATGTGATTCGCCTTGTGAAAAATACAAATTATGTT<br>CAGGTCCGCGAGGACGATCGTATAATACAGACCGGAGCAATTGGATTCGATGCAC<br>TGACATTTGAAGTTTTTGGCTCATTGCTGCATGGAGCTGAATTGTATCCTGTTACT<br>AAAGACGTGCTATTAGATGCAGAGAAACTACACAAATTTTTACAAGCGAATCAAA<br>TTACGATTATGTGGTTAACTTCTCCGTTATTTAACCAATTGTCACAAGGAACCGAA<br>GAGATGTTTGCTGGCCTTCGCTCCCTAATTGTAGGTGGAGATGCCTTGTCTCCGAA<br>ACACATCAATAATGTAAAGCGAAAATGCCCTAATCTGACTATGTGGAACGGTTAC<br>GGCCCAACAGAAAACACCACTTTTTCTACATGCTTTCTTATTGATAAAGAATATGA<br>TGACAATATTCCGATAGGGAAGGCCATTAGTAATTCAACAGTGTATATCATGGACC<br>GGTATGGCCAGCTTCAGCCGGTGGGTGTACCAGGAGAATTATGTGTAGGAGGGG<br>ATGGGGTTGCCAGGGGATATATGAATCAGCCTGCATTAACAGAAGAGAAGTTTGT<br>CCCAAATCCATTCGCTCCTGGTGAGAGAATGTATCGCACGGGGGATTTGGCAAGA<br>TGGTTGCCTGATGGAACAATTGAGTATTTAGGTCGTATTGATCAGCAAGTGAAAAT<br>CAGGGGCTACCGTATTGAACCGGGAGAGATTGAAACGCTTCTTGTGAAGCACAA<br>AAAAGTCAAAGAATCGGTAATCATGGTAGTAGAGGATAATAATGGACAAAAGGCT<br>CTATGCGCTTATTACGTTCCGGAAGAAGAAGTAACGGTATCTGAACTGAGGGAAT<br>ATATAGCTAAAGAGTTGCCTGTTTACATGGTTCCAGCCTATTTTGTACAGATTGAA<br>CAAATGCCTCTTACACAGAACGGTAAAGTAAATCGAAGCGCGTTACCAAAACCA<br>GATGGTGAATTTGGTACAGCAACCGAATATGTAGCGCCTAGCAGCGACATTGAAA<br>TGAAGCTGGCAGAGATTTGGCATAATGTGTTAGGGGTAAACAAAATCGGGGTACT<br>GGATAACTTCTTTGAATTAGGTGGTCATTCATTAAGAGCTATGACAATGATTTCCC<br>AGGTACATAAAGAGTTCGACGTTGAATTGCCATTAAAAGTGTTATTTGAAACACC<br>AACGATCTCTGCATTAGCTCAATACATTGCTGATGGAGAAAAAGGAATGTACCTG<br>GCCATTCAACCTGTTACCCCGCAGGATTACTATCCAGTATCATCTGCGCAAAAGAG<br>GATGTACATCCTTTATGAATTTGAAGGGGCTGGCATTACCTATAATGTACCTAATGT<br>AATGTTTATAGAAGGAAAGCTGGATTATCAGCGCTTTGAATACGCTATAAAAAGTT<br>TGGTAAATCGACATGAGGCGCTTCGAACGTCTTTCTATTCGCTTAATGGAGAACC<br>AGTTCAGCGTGTACATCAAAATGTAGAGCTACAGATTGCTTATTCGGAGGCGAAA<br>GAAGATGAGATAGAGCAAATTGTAGAAAGCTTTGTTCAACCATTTGACCTTGAAA<br>TAGCTCCGCTGCTTCGCGTAGGGCTTGTTAAATTGGCATCGGATCGCTATTTATTCC<br>TAATGGATATGCATCATATTATCTCAGATGGTGTATCAATGCAAATTATAACAAAAG

	AAATTGCCGACTTATATAAAGGAAAAGAGCTTGCTGAACTGCATATTCAGTATAAA<br>GATTTTGCTGTATGGCAAAACGAATGGTTTCAATCTGACGCTCTTGAAAAACAGA<br>AAACGTATTGGTTGAACACCTTTGCAGAGGATATTCCGGTTTTAAATTTGTCAACT<br>GATTATCCAAGACCGACAATTCAAAGTTTTGAAGGAGATATTGTCACGTTTAGTG<br>CAGGGAAGCAACTTGCGGAAGAATTGAAACGCCTGGCTGCAGAAACAGGGACG<br>ACTTTGTATATGCTTCTGTTAGCGGCGTACAATGTACTTTTACACAAATACTCGGG<br>ACAGGAAGAAATTGTAGTAGGAACGCCTATTGCCGGGCGATCTCACGCAGATGTG<br>GAAAATATTGTTGGGATGTTTGTCAATACGCTTGCATTGAAAAATACCCCTATAGC<br>CGTACGCACCTTCCACGAATTCCTGTTGGAAGTAAAACAAAATGCTTTAGAAGCT<br>TTTGAAAATCAAGACTATCCATTTGAAAATTTGATAGAGAAGCTGCAAGTGCGTC<br>GCGACTTAAGTCGCAATCCATTATTTGATACAATGTTTAGCCTAAGCAATATTGAC<br>GAACAAGTAGAGATAGGGATTGAGGGATTGAACTTCAGCCCATATGAAATGCAGT<br>ATTGGATTGCAAAATTTGATATTTCATTCGATATTTTAGAAAAGCAAGATGACATTC<br>AATTTTATTTTAACTATTGCACGAATCTGTTTAAAAAAGAAACGATAGAACGATTA<br>GCGACACACTTTATGCATATTTTACAGGAGATTGTTATTAATCCTGAGATTAAGTTA<br>TGTGAAATTAATATGCTGTCCGAAGAAGAACAGCAGCGTGTCCTGTATGACTTTA<br>ATGGCACAGATGCAACCTACGCTACGAATAAAATATTCCATGAGTTATTTGAAGAA<br>CAGGTTGAAAAAACACCAGATCATATAGCGGTGATAGATGAAAGAGAAAAGCTT<br>TCCTATCAGGAGCTTAATGCGAAAGCGAATCAGCTGGCACGAGTGCTGCGCCAA<br>AAAGGAGTACAGCCTAATAGCATGGTAGGTATTATGGTAGATCGCTCACTCGACAT<br>GATTGTAGGAATGCTTGGGGTTTTAAAAGCAGGAGGAGCATATGTGCCTATCGAT<br>ATAGACTATCCTCAGGAACGGATTAGCTACATGATGGAAGATAGTGGTGCAGCGC<br>TCTTGTTAACACAACAAAAGTTGACACAGCAAATTGCGTTTTCTGGTGACATTTT<br>GTATCTTGACCAAGAAGAATGGCTTCATGAGGAAGCTTCAAATTTAGAACCCATC<br>GCTCGTCCGCAGGATATAGCCTATATCATTTACACTTCTGGTACAACCGGAAAGCC<br>AAAAGGTGTGATGATTGAGCATCAAAGCTATGTGAATGTAGCAATGGCATGGAAA<br>GATGCCTATCGGTTAGATACATTCCCGGTCCGTTTGCTTCAGATGGCTAGCTTTGC<br>CTTTGACGTATCTGCGGGTGATTTTGCCAGAGCACTACTTACAGGTGGGCAATTA<br>ATTGTATGTCCAAATGAAGTAAAGATGGACCCAGCTTCTTTATATGCCATTATTAAG<br>AAATATGACATTACTATTTTTGAAGCAACGCCTGCTCTAGTGATTCCATTGATGGA<br>GTATATTTATGAACAGAAGCTGGATATTAGCCAGTTACAGATTCTGATTGTCGGATC<br>GGACAGTTGTTCGATGGAGGACTTTAAAACCTTGGTTTCCCGTTTTGGTTCAACT<br>ATACGTATTGTGAATAGCTATGGAGTAACCGAAGCGTGCATTGATTCTAGCTATTAT<br>GAACAACCGCTTTCTTCGTTACATGTAACAGGAACTGTACCGATTGGAAAACCGT<br>ACGCTAACATGAAAATGTATATTATGAATCAATATTTGCAGATTCAGCCTGTAGGTG<br>TAATTGGAGAATTATGTATTGGAGGAGCCGGGGTTGCCCGTGGATATTTAAATAGA<br>CCGGACTTAACAGCAGAAAAGTTTGTCCCTAATCCTTTTGTTCCAGGTGAAAAGC<br>TGTATCGAACAGGCGACTTGGCAAGATGGATGCCGGATGGGAATGTTGAGTTTCT<br>TGGTCGAAATGACCATCAGGTGAAAATCAGAGGGATTCGAATCGAGCTTGGAGA<br>AATCGAAGCACAACTGCGTAAACATGATAGCATAAAAGAAGCAACTGTGATCGC<br>AAGAGAAGATCACATGAAAGAGAAATATTTATGTGCGTATATGGTGACCGAAGGA<br>GAAGTAAATGTAGCTGAACTGCGTGCGTATCTAGCAAATGATCTGCCTGCGGCAA<br>TGATTCCGTCATATTTTGTATCGCTCGAAGCAATGCCACTTACTGCTAATGGAAAA<br>ATTGATAAGCGATCTTTACCAGAGCCCGATGGTTCCATATCGATAGGAACAGAATA<br>TGTAGCTCCGCGTACCATGCTTGAGGGAAAACTAGAAGAGATATGGAAAGATGTA<br>TTGGGTTTACAGCGTGTTGGCATTCACGATGACTTCTTTACAATAGGTGGCCATTC<br>ATTGAAGGCTATGGCTGTTATTTCGCAAGTTCATAAAGAATGCCAGACTGAAGTT<br>CCTCTGCGTGTCTTATTTGAAACACCTACCATTCAAGGACTGGCTAAATATATAGA<br>GGAAACGGACACAGAGCAATATATGGCTATTCAGCCGGTTAGCGGACAGGACTAT<br>TATCCAGTATCATCAGCACAAAAGAGAATGTTTATTGTTAATCAATTTGATGGAGT<br>AGGAATTAGCTACAATATGCCTTCCATCATGCTGATTGAAGGAAAACTTGAGCGA<br>ACACGCTTGGAATCAGCATTTAAAAGATTGATAGAACGACATGAGAGCCTTCGAA<br>CATCTTTTGAAATAATAAATGGTAAGCCTGTACAGAAGATTCATGAGGAAGTTGAT<br>TTCAATATGTCCTATCAGGTGGCTTCTAATGAACAAGTAGAGAAGATGATCGATGA<br>GTTCATTCAGCCTTTCGATTTAAGTGTTGCACCGCTGCTTCGTGTGGAACTTTTAA<br>AATTGGAAGAGGACCGTCATGTGCTTATATTTGATATGCATCATATTATCTCAGATG<br>GTATATCTTCCAATATTTTGATGAAAGAATTAGGAGAACTATATCAAGGTAATGCTT

	TACCAGAACTTCGTATTCAATACAAGGATTTCGCTGTATGGCAAAATGAGTGGTTC<br>CAGTCAGAAGCCTTTAAAAAGCAAGAAGAATACTGGGTAAATGTTTTCGCAGAT<br>GAACGCCCGATTCTGGATATACCGACGGATTATCCAAGGCCGATGCAACAAAGCT<br>TTGATGGTGCTCAACTTACATTTGGAACCGGAAAGCAGCTTATGGATGGGTTATAC<br>AGGGTAGCAACGGAAACGGGAACAACGCTTTATATGGTTTTGCTTGCGGCATATA<br>ATGTTCTTCTTTCCAAATATTCTGGTCAAGAAGATATTATTGTAGGGACACCGATTG<br>TGGGTAGATCCCATACTGACCTTGAGAATATTGTCGGGATGTTTGTCAACACGTTA<br>GCAATGAGAAATAAACCGGAAGGAGAAAAGACGTTCAAAGCATTTGTATCAGAA<br>ATAAAGCAGAATGCACTAGCGGCTTTTGAGAATCAGGATTATCCATTTGAGGAGC<br>TTATCGAAAAACTAGAGATACAAAGGGACTTAAGCAGAAATCCATTATTTGATAC<br>GCTCTTTAGCCTTCAAAACATAGGTGAAGAATCATTTGAACTAGCCGAATTAACAT<br>GCAAACCTTTCGATTTGGTAAGCAAATTAGAGCATGCCAAGTTTGATCTGAGTCT<br>TGTGGCAGTAGAAAAAGAGGAAGAAATTGCATTTGGGCTTCAATACTGCACAAA<br>ACTGTATAAGGAAAAAACAGTTGAACAACTGGCTCAACATTTTATTCAAATAGTA<br>AAAGCAATTGTAGAAAATCCAGATGTCAAATTATCTGATATTGATATGTTATCTGAA<br>GAAGAGAAGAAACAAATCATGCTTGAGTTCAATGATACGAAAATACAATATCCGC<br>AGAATCAAACAATACAGGAATTGTTTGAGGAGCAAGTGAAGAAAACACCTGAAC<br>ATATAGCAATCGTATGGGAAGGGCAAGCATTAACCTATCATGAGCTAAATATAAAA<br>GCTAATCAGTTAGCTCGTGTATTACGAGAAAAAGGGGTAACCCCTAATCATCCTGT<br>AGCGATTATGACGGAACGCTCATTAGAGATGATCGTAGGTATCTTTAGTATTTTGA<br>AAGCAGGAGGAGCATATGTTCCAATTGATCCAGCCTATCCACAAGAACGTATTCA<br>ATACTTGCTTGAAGATAGCGGAGCGACGCTACTGCTTACTCAGTCACATGTATTAA<br>ATAAATTACCGGTCGATATCGAATGGTTGGATCTTACAGATGAACAAAACTATGTA<br>GAAGATGGTACCAATCTTCCATTTATGAATCAGTCAACAGATCTTGCCTATATTATT<br>TATACATCCGGTACAACAGGCAAGCCTAAAGGGGTTATGATTGAACATCAAAGCA<br>TCATCAACTGCCTGCAATGGCGGAAGGAAGAATACGAATTTGGACCAGGGGATA<br>CGGCTCTACAAGTGTTTTCCTTTGCTTTTGATGGATTTGTAGCAAGTTTGTTTGCT<br>CCGATTCTTGCTGGTGCAACGTCTGTTCTCCCTAAGGAGGAAGAAGCAAAAGATC<br>CAGTTGCATTGAAAAAACTGATCGCATCAGAAGAGATTACACATTACTACGGTGT<br>GCCTAGTTTGTTTAGTGCCATTCTTGATGTTTCTTCTAGTAAGGATTTGCAAAATTT<br>ACGCTGCGTCACTTTGGGAGGAGAGAAATTACCGGCTCAAATTGTTAAAAAAATC<br>AAAGAAAAAAATAAAGAAATTGAAGTCAACAACGAATATGGGCCTACTGAAAAT<br>AGTGTAGTAACTACTATTATGCGCGATATACAGGTAGAACAAGAGATTACTATTGG<br>TCGCCCATTATCTAACGTAGATGTATATATTGTCAATTGTAATCATCAATTACAACCA<br>GTAGGTGTAGTAGGGGAATTATGTATTGGTGGACAGGGACTTGCAAGAGGATATT<br>TGAATAAACCAGAGCTTACAGCAGATAAATTTGTTGTAAATCCATTCGTACCTGGT<br>GAACGTATGTACAAAACCGGTGACCTTGCAAAATGGCGCTCAGATGGAATGATTG<br>AATATGTGGGGCGTGTTGATGAACAAGTAAAAGTAAGAGGATATCGGATTGAGCT<br>TGGTGAAATTGAATCAGCTATCCTAGAATACGAAAAAATTAAGGAAGCGGTAGTT<br>ATGGTTTCGGAGCATACTGCATCTGAACAGATGTTATGTGCTTATATTGTAGGGGA<br>AGAAGATGTACTGACTCTGGACTTAAGAAGCTATCTAGCAAAATTACTACCAAGT<br>TATATGATTCCAAACTATTTTATCCAATTGGATAGTATTCCGCTTACACCAAACGGT<br>AAAGTGGATCGTAAAGCATTGCCTGAACCTCAAACCATTGGCTTAATGGCAAGGG<br>AGTATGTTGCACCAAGGAATGAAATCGAAGCACAGCTAGTACTCATTTGGCAAGA<br>GGTATTAGGAATAGAACTGATCGGTATTACCGATAATTTCTTTGAATTAGGAGGGC<br>ATTCTTTAAAGGCAACGCTTTTAGTTGCAAAAATTTACGAGTACATGCAAATAGA<br>GATGCCATTAAATGTTGTGTTTAAACATTCAACTATTATGAAAATAGCGGAATATAT<br>TACACATCAAGAATCAGAAAATAATGTACATCAGCCTATTTTGGTAAATGTAGAAG<br>CAGATAGAGAGGCGCTATCTCTTAACGGCGAGAAGCAAAGAAAAAATATAGAGC<br>TACCTATTCTGCTAAACGAAGAAACAGATCGAAACGTATTCTGCTTCGCGCCCATT<br>GGTGCACAAGGTGTTTTTTATAAAAAGCTTGCTGAACAAATCCCTACTGCATCCTT<br>GTATGGCTTTGACTTCATTGAAGATGATGATCGAATTCAGCAATATATTGAATCGAT<br>GATTCAAACTCAGTCAGACGGACAATATGTGCTAATTGGTTATTCTTCAGGAGGG<br>AACCTGGCTTTTGAAGTAGCAAAAGAAATGGAAAGGCAAGGATATAGTGTATCTG<br>ATTTGGTCTTGTTCGATGTTTACTGGAAGGGAAAAGTATTCGAGCAAACAAAAGA<br>AGAAGAAGAAGAAAACATAAAAATAATAATGGAAGAATTAAGGGAAAATCCAGG<br>AATGTTCAATATGACACGAGAGGATTTTGAACTGTATTTTGCGAATGAATTTGTGA

	AACAAAGTTTCACACGGAAAATGCGCAAATACATGAGTTTTTATACGCAGTTAGT<br>TAATTATGGGGAAGTAGAAGCTACAATTCACCTTATACAAGCAGAATTTGAGGAA<br>GAAAAAATTGACGAAAACGAAAAAGCCGACGAAGAAGAAAAAACATATCTAGA<br>GGAAAAATGGAATGAAAAAGCATGGAACAAAGCAGCAAAAAGATTTGTAAAATA<br>TAACGGATATGGCGCTCATTCTAACATGCTAGGAGGTGATGGTTTAGAGAGAAATT<br>CCTCTATCCTTAAACAGATACTACAAGGGACATTTGTAGTAAAATAAAAGAAGAA<br>GTGTGAAAAAGCGCAGCTGAAATAGCTGCGCTTTTTTGTGTCATAA
pGETS151<br>2nd-1E9	AAAAGGCCTTCTTGGCCGCCCTTCCCGGTCGATATGAACAGCTTATTTACATAATT<br>CACGTTATTGGTAGTTATAAATGAAATTCCTAATATCGGTTATGAAGTGAAATTGAA<br>TTTCTACTTGATCTTTCTCTCTATTTTTGTAAAATAAAATTAAGAATATTTAAATATT<br>CAATGATTCATTTTTGCAGAAATCGGAGGAAGAAGAATATATGAAAACATTTAAC<br>ATTTCTCAACAAGATCCCCCCATATTGTTGTATAAGTGATGAAATACTGAATTTAAA<br>ACCTAGTTTATATGTGGTAAAATGTTTTAATCAAGTTTAGGAGGAATTAATTATGAA<br>GTGTAATGAATAATGAGTGTAACAGGGTTCAATTAAAAGAGGGAAGCGTATCATT<br>AACCCTATAAACTACGTCTGCCCTCATTATTGGAGGGTGAAATGTGAATACATCCT<br>ATTCACAATCGAATTTACGACACAACCAAATTTTAATTTGGCTTTGCATTTTATCTT<br>TTTTTAGCGTATTAAATGAAATGGTTTTGAACGTGTCATTACCTGATATTGCAAATG<br>ATTTTAATAAACCACCAGCGAGTACAAACTGGGTGAACACAGCCTTTATGTTAAC<br>CTTTTCCATTGGAACAGCTGTATATGGAAAGCTATCTGATCAATTAGGCATCAAAA<br>GGTTACTCCTATTTGGAATTATAATAAATTGTTTCGGGTCGGTAATTGGGTTTGTTG<br>GCCATTCTTTCTTTTCCTTACTTATTATGGCTCGTTTTATTCAAGGGGCTGGTGCAG<br>CTGCATTTCCAGCACTCGTAATGGTTGTAGTTGCGCGCTATATTCCAAAGGAAAAT<br>AGGGGTAAAGCATTTGGTCTTATTGGATCGATAGTAGCCATGGGAGAAGGAGTCG<br>GTCCAGCGATTGGTGGAATGATAGCCCATTATATTCATTGGTCCTATCTTCTACTCA<br>TTCCTATGATAACAATTATCACTGTTCCGTTTCTTATGAAATTATTAAAGAAAGAAG<br>TAAGGATAAAAGGTCATTTTGATATCAAAGGAATTATACTAATGTCTGTAGGCATT<br>GTATTTTTTATGTTGTTTACAACATCATATAGCATTTCTTTTCTTATCGTTAGCGTGC<br>TGTCATTCCTGATATTTGTAAAACATATCAGGAAAGTAACAGATCCTTTTGTTGATC<br>CCGGATTAGGGAAAAATATACCTTTTATGATTGGAGTTCTTTGTGGGGGAATTATAT<br>TTGGAACAGTAGCAGGGTTTGTCTCTATGGTTCCTTATATGATGAAAGATGTTCAC<br>CAGCTAGGTACTGCCGAAATCGGAAGTGTAATTATTTTCCCTGGAACAATGAGTG<br>TCATTATTTTCGGCTACATTGGTGGGATACTTGTTGATAGAAGAGGTCCTTTATACG<br>TGTTAAACATCGGAGTTACATTTCTTTCTGTTAGCTTTTTAACTGCTTCCTTTCTTT<br>TAGAAACAACATCATGGTTCATGACAATTATAATCGTATTTGTTTTAGGTGGGCTTT<br>CGTTCACCAAAACAGTTATATCAACAATTGTTTCAAGTAGCTTGAAACAGCAGGA<br>AGCTGGTGCTGGAATGAGTTTGCTTAACTTTACCAGCTTTTTATCAGAGGGAACA<br>GGTATTGCAATTGTAGGTGGTTTATTATCCATACCCTTACTTGATCAAAGGTTGTTA<br>CCTATGGAAGTTGATCAGTCAACTTATCTGTATAGTAATTTGTTATTACTTTTTTCA<br>GGAATCATTGTCATTAGTTGGCTGGTTACCTTGAATGTATATAAACATTCTCAAAG<br>GGATTTCTAAATCGTTAAGGGATCAACTTTGGGAGAGAGTTCAAAATTGATCCTTT<br>TTTTATAACAGGAATTGGGCATCAAATAAAACGAAAGACTGGGCCTTTCGTTTTAT<br>CTGTTGTTTGTCGGTGAACGCTCTCCTGAGTAGGACAAGTCCGCCGGGAGCGGAT<br>TTGAACGTTGCGAAGCAACGGCCCGGAGGGTGGCGGGCAGGACGCCCGCCATA<br>AACTGCCAGGCATCAAATTAAGCAGAAGGCCATCCTGACGGATGGCCTTTTTGCG<br>TTTCTACAAACTCTTCCTGTCGTCATATCTACAATTCTACACAGCCCAGTCCAGAC<br>TATTGAATTGTATCACGGTTTTGATATCCTACCAATAACAAATTGATTGGAGGAATG<br>CAAAGTGAATAATGAACCAGTAAAACGTGGTAAGAAGAACAGATGGGAATTAAA<br>CCTACCTATAATGACTTATGTAGTAGCTGATGATTGGATTGATAAACTAGGACACG<br>AAACGTTTACTTTATGGTTGAGGTTCCATACTTGGGTAGATAGAGAAGATGAACTC<br>CGAGATTATGATCGCATACCTAGAAGTTTTGAGAACATATATAAAAAGACACTAGG<br>AATCTCAAAAAGTAAGTTTTATAGATTGATAAAACCTTTATGGGAATATGGATTAAT<br>AGACATCATAGAATACGAAGAATCTAACCGTAATTCTACTAAACCTAAAAATATAA<br>TTGTTTATGAGTATCCTTTACACGAAATAGAAAGAAAGTATAAACCACTAGAAAA<br>ATTAAGAGATTGGGATAAAGACTATAATTCCGTTTCTAAAGAATTAGGTAAAACAG<br>GTGGTAGACCAAGGAAAAAAGATAGTGAAGAAGAACCCGAAAAGAAACCCGAA<br>GAAGTAACTAAAAAGAAACGTAAATATAAGTTAAAAAGAGTTATCCACAACGGTT<br>TCAAAAATGAAACGGTGGAGGGTTTCAAAAATGAAACGGTGGAGGGTTTCAAA

	AATGAAACGGTGACCGTTTCAAAAATAAAACCCAATAATTATTCAAATATCTTTAA<br>TAACTTATCAAATATTTCTACTAATGTTTCAAATAATTTATTAATTGATGATGATGAG<br>GAAATCGAAAATGAACCAACTGGTCGTACAATAAATAGGTCATTACTTTTTTCGCA<br>AGAAGATATTAAACAGGCCTATCAATTTATTAATAGATTTTCAGTTATACAGTTACG<br>TGAAAACTTTAGCTTTGATAAACACTTTGAAGAACGGTTGGTATGTTATTTATGGA<br>AAGCAGGGATTTCTACTTTTTACACGCACGAAATCAGTAAAATGATAAAAAAAAT<br>AGCAGACTATGAAAAATCTAAAAAAGGTAGATTAAACCCAATACGTGACCGAGC<br>CTTATATATGGTAAATGGTCTTGTAATGAATAGAGCTTCTTCCCAAAGTGAACATG<br>CTACTTATAAACTAAACCAATATAAAAAACAGAAGGAACAGGAAAAACAACAAC<br>AGGAGCAACAAAGATCAAGAGTACCGTTCTATAATTGGTTGGAGGAAAGAGAAG<br>AACAAACCGAAGGTCAACTACCCACCACTTAAGCGGCCGCAAGCTTGAAGAGCT<br>CTTCTTTCAGAACGCTCGGTTGCCGCCGGGCGTTTTTTATGAGATGTCTCGGCCTG<br>TTTGGCCATTAATCGAAGAAGAAGTGTGAAAAAGCGCAGCTGAAATAGCTGCGC<br>TTTTTTGTGTCATAATCCTTCGATATATCGCGTCTATTCCGGCTTCCGGCTATCACCC<br>GAAGATAAACAGCCCAGGGGTCACAGATGAAGTACTGAAGAAAATGAGGAACG<br>GTTTGATTAAGGTAAGGCCGTATACAGTCAATCGTCCGGAAGATATGAAGCGTCT<br>CATTGAAGCGGGAGCAGACGGCATGTTTACCGACTTTCCAGAAAAGGCTTCGGC<br>ATTGCTGAAAAATGAATAGTTGTTAGAAGGAGGCTGTTTGACGCAGCCTTCTTTT<br>TTCATTCATTCATGCCCGTTTCAAAGCATACATTCATAGAAGACGGAAGAATAAAG<br>GGAGAGGTGAATGTGACTTTTATTTCACAAGTAAATAAATGGTTTGTTAATGCTAA<br>TGTTAACTCAGCTGCAAAGCTTAGGCTATTCTGTATTCCATATGCAGGCGGTGGTG<br>CTTCCGCCTTTTATGAATGGAGTCATTTTTTTCCAAAGGAAATTGAAGTTTGTTCA<br>ATTCAATTACCTGGAAGGGAAAATAGGGGGGCGGAAGTTCCGCTAACAAATTTAC<br>AACAGATAGTAGAAATAGTAGCTGAGGAAATACAACCATTAATAAATATTCCATTT<br>GCTTTTTTGGGGCATAGCATGGGAGCATTAATAAGTTTTGAACTGGCTCGCACAAT<br>ACGGCAAAAGAGTAATGTTAATCCGGTTCACTTGTTTGTTTCAGGGCGACATGCA<br>CCTCAAATCCCATGTGCAAAACAAGACTATCATTTACTTCCCGATGAACAATTTAT<br>ACAAGAATTGCGTTCATTGAATGGAACTCCAGAGATAGTATTACAAGACGCAGAG<br>ATGATGAGTATATTACTCCCAAGACTTCGGGCTGATTTTTCTGTGTGTGGCTCCTAT<br>CAGTACAAAAACGACGAGCCTTTTGAATGCCCAATCACTGCTTTTGGAGGAAAA<br>AATGATAATGGTGTTACTTATCAATCATTAGAAGCCTGGAGAGAGCAAACCAAGA<br>GGGAATTTTCTGTGTGTATGTATCCAGGTGATCATTTTTTTCTTTACGAAAGCAAAT<br>ATGAAATGATTGAGTTCATGTGTAAACAATTACGTTTAGTATTAGCTCCTAAAATAT<br>AAGGCCTTGATGGCCATCGAAGAAGAAGTGTGAAAAAGCGCAGCTGAAATAGCT<br>GCGCTTTTTTGTGTCATAATCCTGACGCTCTTCGCAAGGGTGTCTTTTTTTGCCTTT<br>TTTTCGGTTTTTGCGCGGTACACATAGTCATGTAAAGATTGTAAATTGCATTCAGC<br>AATAAAAAAAGATTGAACGCAGCAGTTTGGTTTAAAAATTTTTATTTTTCTGTAAA<br>TAATGTTTAGTGGAAATGATTGCGGCATCCCGCAAAAAATATTGCTGTAAATAAAC<br>TGGAATCTTTCGGCATCCCGCATGAAACTTTTCACCCATTTTTCGGTGATAAAAAC<br>ATTTTTTTCATTTAAACTGAACGGTAGAAAGATAAAAAATATGGAACTATTTTTTA<br>CAGGGGGTATATATGTTAAACAGTTCTAAAAGTATATTGATTCATGCTCAAAATAA<br>AAATGGAACGCATGAAGAGGAGCAGTATCTCTTTGCTGTGAACAACACCAAAGC<br>GGAGTATCCACGTGATAAGACGATCCATCAGTTATTTGAGGAGCAGGTTAGTAAG<br>AGGCCAAACAATGTAGCCATTGTATGTGAAAATGAGCAACTTACCTACCATGAGC<br>TTAATGTGAAAGCCAATCAACTAGCACGGATTTTTATAGAAAAAGGGATTGGAAA<br>AGACACTCTTGTTGGAATTATGATGGAGAAATCTATCGATTTATTTATAGGCATATT<br>AGCCGTTTTAAAAGCTGGTGGAGCATATGTTCCGATTGATATTGAATATCCTAAGG<br>AAAGAATTCAATATATTCTTGATGATAGTCAGGCAAGAATGCTACTTACCCAGAAG<br>CATTTGGTTCATTTAATTCATAATATTCAATTTAATGGGCAAGTGGAAATTTTTGAA<br>GAAGATACTATCAAAATTAGAGAAGGAACTAATCTACATGTACCAAGTAAATCAA<br>CCGATCTTGCTTATGTTATTTATACTTCTGGTACAACAGGCAATCCAAAAGGTACA<br>ATGCTGGAGCATAAAGGAATAAGTAATCTAAAGGTATTTTTCGAAAATAGTCTTAA<br>CGTGACTGAAAAGGATAGAATTGGTCAATTTGCCAGCATCTCTTTTGATGCATCTG<br>TATGGGAGATGTTTATGGCTTTGTTAACGGGGGCTAGCCTGTATATTATCCTGAAG<br>GATACAATCAATGATTTTGTGAAGTTTGAACAATACATTAACCAAAAGGAAATCA<br>CTGTTATTACGTTACCACCTACCTATGTAGTTCATCTTGATCCAGAACGTATTTTATC<br>GATACAAACGTTAATTACAGCAGGCTCAGCTACCTCGCCTTCCTTAGTAAACAAG

	TGGAAGGAGAAAGTAACTTACATAAATGCCTATGGCCCTACGGAAACAACTATTT<br>GTGCGACTACATGGGTAGCCACCAAAGAAACAATAGGTCATTCAGTTCCAATCGG<br>AGCACCAATTCAAAATACACAAATTTATATTGTCGATGAAAATCTTCAATTAAAAT<br>CGGTTGGTGAAGCTGGTGAATTGTGTATTGGTGGAGAAGGGTTAGCAAGGGGAT<br>ATTGGAAGCGACCGGAATTAACTTCCCAGAAGTTCGTTGATAACCCGTTTGTTCC<br>AGGAGAGAAGTTGTATAAAACAGGAGATCAGGCAAGATGGCTATCTGATGGAAA<br>TATTGAATATCTCGGAAGAATAGATAACCAGGTAAAGATTAGAGGTCACCGAGTT<br>GAACTAGAAGAAGTTGAGTCTATTCTTCTAAAGCATATGTATATTAGCGAAACTGC<br>AGTAAGTGTGCATAAAGATCACCAAGAACAGCCGTATTTGTGCGCTTATTTTGTAT<br>CGGAAAAGCATATACCACTAGAACAGTTAAGACAATTCTCATCAGAAGAACTGCC<br>AACGTATATGATCCCTTCTTATTTTATCCAGTTAGACAAAATGCCGCTTACATCAAA<br>TGGGAAGATTGATCGAAAGCAGTTGCCGGAACCTGATTTAACTTTCGGGATGAGG<br>GTAGACTATGAAGCGCCGCGAAATGAAATCGAGGAAACGCTTGTTACTATCTGGC<br>AGGATGTATTAGGTATTGAGAAAATCGGTATTAAAGATAATTTCTATGCATTAGGTG<br>GAGATTCTATTAAAGCAATACAGGTTGCTGCTCGCCTGCATTCCTACCAATTAAAG<br>CTAGAAACAAAAGATTTATTAAAGTATCCAACAATCGATCAACTCGTTCATTATAT<br>AAAAGATAGTAAAAGAAGAAGTGAGCAAGGTATTGTGGAAGGTGAGATTGGACT<br>TACACCTATTCAGCATTGGTTCTTTGAACAACAATTTACAAATATGCACCATTGGA<br>ACCAATCGTATATGTTGTATAGACCAAATGGGTTTGATAAAGAGATCTTGCTAAGG<br>GTATTTAATAAAATTGTTGAGCATCATGATGCATTACGTATGATATACAAACATCAT<br>AACGGAAAGATCGTGCAGATAAATCGGGGGCTTGAAGGTACGTTGTTTGATTTTT<br>ATACCTTTGATTTAACTGCAAATGATAATGAGCAACAGGTGATTTGTGAAGAATCT<br>GCTCGATTACAAAATAGTATAAACTTGGAAGTAGGCCCTCTAGTAAAGATAGCGCT<br>GTTTCATACTCAGAATGGAGATCACCTGTTTATGGCTATTCATCATTTGGTTGTGGA<br>TGGTATTTCTTGGAGGATTTTGTTTGAGGATTTGGCCACAGCTTATGAACAAGCAA<br>TGCATCAGCAAACGATTGCTTTACCAGAGAAAACAGATTCATTTAAGGACTGGTC<br>TATTGAATTAGAAAAATATGCGAACAGCGAATTATTCCTAGAAGAAGCTGAATATT<br>GGCATCATTTGAATTATTATACCGAGAACGTTCAAATTAAGAAAGATTATGTCACC<br>ATGAACAATAAACAAAAGAATATACGTTATGTAGGAATGGAGTTAACAATAGAAG<br>AGACAGAAAAATTATTGAAAAATGTAAATAAAGCGTATCGAACAGAAATTAATGA<br>TATTTTATTAACGGCACTTGGCTTTGCACTCAAAGAATGGGCCGATATTGATAAAA<br>TTGTAATTAACTTAGAGGGACACGGACGGGAAGAAATACTGGAACAGATGAACA<br>TTGCAAGGACGGTAGGCTGGTTTACTTCCCAGTATCCTGTTGTACTTGATATGCAA<br>AAATCGGATGATTTGTCTTATCAAATCAAATTAATGAAAGAAAATTTACGCAGAAT<br>ACCTAACAAAGGAATCGGATATGAAATTTTTAAGTATTTAACAACTGAATATTTAC<br>GGCCTGTTTTACCCTTTACATTAAAGCCGGAAATTAACTTTAACTACTTAGGACAG<br>TTCGATACGGACGTGAAAACTGAATTGTTTACTCGTTCTCCTTATAGCATGGGTAA<br>TTCATTAGGACCAGATGGAAAAAATAATTTAAGCCCAGAAGGGGAAAGTTATTTT<br>GTACTCAATATTAATGGTTTTATTGAAGAAGGTAAGCTTCACATCACCTTTTCTTAT<br>AATGAACAGCAGTATAAGGAGGATACCATTCAGCAATTGAGCCGGAGCTATAAGC<br>AACATCTTTTGGCCATCATTGAACATTGTGTACAGAAGGAAGATACTGAGTTAAC<br>TCCAAGTGATTTCAGTTTCAAGGAACTTGAATTAGAAGAGATGGATGATATTTTCG<br>ATTTGTTGGCCGATTCATTAACGTAAGGCCTCGATGGCCATCGAAGAAGAAGTGT<br>GAAAAAGCGCAGCTGAAATAGCTGCGCTTTTTTGTGTCATAATCCTTATGGGAAG<br>TGCTCCGTAATACGCTGACAAGAGAGAAAGGGCTTGGAGGTATTGAAACAAGAG<br>GAGTTCTGAGAATTGGTATGCCTTATAAGTCCAATTAACAGTTGAAAACCTGCATA<br>GGAGAGCTATGCGGGTTTTTTATTTTACATAATGATACATAATTTACCGAAACTTGC<br>GGAACATAATTGAGGAATCATAGAATTTTGTCAAAATAATTTTATTGACAACGTCT<br>TATTAACGTTGATATAATTTAAATTTTATTTGACAAAAATGGGCTCGTGTTGTACAA<br>TAAATGTAGTGGGAACATTATTATGAGGTGCTAGCATGAGTACATTTAAAAAAGAA<br>CATGTTCAGGATATGTATCGTTTATCTCCCATGCAGGAAGGCATGTTGTTTCACGC<br>ATTACTTGATAAAGATAAAAATGCTCACCTGGTACAAATGTCTATCGCGATCGAAG<br>GTATCGTGGATGTGGAGCTGCTTAGTGAAAGCTTGAACATATTGATTGATAGATAC<br>GATGTGTTTAGAACAACATTCTTACATGAAAAAATTAAACAACCGCTTCAGGTAG<br>TGCTAAAGGAACGGCCTGTTCAGCTTCAATTTAAAGACATATCATCCTTAGATGAA<br>GAAAAAAGAGAACAGGCTATTGAGCAGTATAAGTATCAAGATGGGGAAACAGTC<br>TTTGATTTAACAAGAGATCCCTTGATGAGAGTAGCTATTTTTCAAACTGGTAAGGT

	TAACTACCAAATGATCTGGAGCTTCCACCATATTTAATGGATGGTTGGTGCTTCA<br>ACATTATATTTAATGACTTGTTCAATATATATCTGTCATTAAAAGAGAAGAAACCTC<br>TTCAGTTAGAGGCGGTGCAACCATATAAGCAGTTTATTAAGTGGCTTGAAAAACA<br>AGATAAACAGGAAGCACTTCGCTACTGGAAAGAACATTTAATGAATTATGATCAA<br>TCAGTAACATTACCTAAAAAGAAAGCAGCTATTAATAATACTACATATGAACCAGC<br>ACAGTTTCGTTTTGCGTTTGACAAAGTGCTTACCCAGCAGCTGCTTCGTATTGCC<br>AATCAAAGCCAAGTAACACTAAATATTGTTTTTCAAACAATATGGGGGATTGTACT<br>TCAAAAATACAATTCCACTAATGATGTTGTATATGGCTCTGTTGTATCAGGCCGTCC<br>TTCTGAAATATCGGGAATTGAGAAAATGGTTGGACTATTTATTAATACTCTTCCATT<br>ACGTATCCAAACGCAAAAAGATCAATCATTTATTGAATTAGTAAAGACTGTTCATC<br>AAAACGTCCTTTTCTCGCAACAGCATGAGTATTTTCCATTGTATGAAATACAAAAT<br>CATACAGAATTAAAACAGAATCTGATTGATCATATTATGGTAATTGAAAATTATCCT<br>TTAGTAGAAGAATTGCAAAAGAATAGTATCATGCAAAAAGTAGGGTTTACAGTTC<br>GTGATGTCAAAATGTTTGAACCAACTAATTATGATATGACAGTTATGGTTTTACCTC<br>GTGATGAAATTAGTGTCCGACTCGATTATAACGCAGCCGTTTATGATATAGATTTCA<br>TAAAAAAAATTGAAGGTCACATGAAAGAAGTGGCTTTATGCGTGGCAAATAATCC<br>ACATGTGTTAGTACAGGACGTTCCTCTGCTTACAAAGCAAGAAAAACAACATTTA<br>TTGGTAGAGCTGCATGATTCGATAACAGAGTATCCTGATAAGACGATTCATCAGTT<br>ATTTACAGAACAGGTAGAAAAAACACCAGAGCATGTGGCAGTTGTATTCGAAGA<br>TGAGAAAGTGACCTATAGAGAGCTGCATGAGAGATCTAATCAATTAGCCAGATTC<br>TTAAGAGAAAAAGGCGTAAAAAAAGAAAGCATCATAGGCATTATGATGGAGCGT<br>TCAGTTGAAATGATTGTTGGGATCTTAGGGATTTTAAAAGCTGGTGGAGCTTTTGT<br>GCCTATTGATCCTGAATATCCAAAAGAAAGAATCGGCTATATGTTAGATTCTGTAC<br>GGCTAGTACTTACACAACGCCATTTAAAGGATAAATTTGCTTTTACGAAAGAAAC<br>GATAGTAATTGAAGATCCAAGTATTTCACACGAGTTAACTGAAGAAATAGATTATA<br>TTAATGAATCAGAGGACTTGTTTTATATTATTTATACATCAGGAACAACAGGTAAA<br>CCAAAAGGGGTTATGCTAGAGCACAAAAACATCGTTAATCTGCTTCATTTTACTTT<br>CGAGAAAACAAATATCAACTTTAGTGACAAAGTATTACAGTATACAACATGCAGT<br>TTTGACGTGTGTTACCAAGAAATTTTTTCGACGCTCTTGTCTGGAGGGCAATTATA<br>TCTTATTAGGAAAGAAACTCAACGCGATGTAGAGCAATTATTTGATTTAGTAAAAC<br>GTGAAAATATTGAAGTATTATCCTTTCCTGTGGCTTTTCTAAAATTTATTTTCAATG<br>AAAGAGAATTTATCAATCGTTTTCCAACTTGCGTGAAACATATTATCACAGCAGGA<br>GAACAATTAGTAGTTAACAATGAGTTTAAACGTTATTTGCATGAACATAACGTACA<br>TTTACACAATCATTATGGTCCATCAGAAACGCATGTTGTTACCACCTATACTATTAA<br>TCCTGAAGCTGAAATTCCTGAATTACCACCGATAGGAAAACCTATCTCCAATACAT<br>GGATTTATATTTTGGATCAAGAACAACAACTACAACCACAAGGAATTGTAGGAGA<br>GTTATATATTTCGGGCGCAAATGTTGGAAGAGGATATTTGAATAATCAAGAATTAA<br>CGGCAGAAAAATTCTTTGCAGATCCCTTTAGGCCAAACGAACGGATGTACCGAAC<br>AGGGGATTTAGCAAGGTGGTTGCCAGACGGAAATATCGAATTTTTAGGAAGGGCC<br>GATCATCAGGTGAAAATTAGGGGGCATCGAATAGAGCTTGGTGAGATCGAGGCAC<br>AATTATTAAATTGTAAGGGTGTAAAAGAAGCTGTTGTTATCGATAAAGCGGATGAT<br>AAAGGCGGAAAATATTTATGTGCCTATGTTGTTATGGAAGTAGAAGTAAATGACTC<br>TGAGCTTCGAGAATATTTGGGGAAAGCTTTGCCTGATTATATGATCCCGTCGTTCT<br>TTGTTCCGTTGGATCAGCTGCCGCTTACACCAAACGGAAAAATAGACAGAAAATC<br>TCTTCCGAATCTAGAGGGGATTGTGAATACAAACGCAAAATATGTAGTACCTACA<br>AATGAGCTGGAAGAAAAATTGGCTAAAATCTGGGAAGAAGTACTTGGGATTTCTC<br>AGATCGGTATACAAGACAATTTCTTTTCGTTAGGCGGGCATTCTCTTAAAGCCATT<br>ACGCTTATTTCCCGTATGAACAAAGAGTGTAATGTAGACATTCCTCTACGTTTGTT<br>ATTTGAAGCACCAACCATTCAGGAAATCTCTAATTATATAAACGGGGCAAAGAAA<br>GAAAGCTATGTTGCCATTCAGCCTGTACCAGAACAAGAGTACTATCCTGTATCATC<br>AGTTCAAAAAAGAATGTTTATTCTTAATGAATTTGATCGTTCAGGTACGGCCTATA<br>ATTTACCTGGTGTTATGTTTCTAGATGGAAAATTGAACTACCGACAATTGGAAGCA<br>GCGGTAAAAAAATTAGTTGAGCGACATGAAGCGCTGCGTACTTCCTTTCATTCAA<br>TTAATGGGGAACCAGTTCAGCGGGTGCATCAAAATGTAGAACTGCAGATTGCTTA<br>TTCAGAGTCAACGGAAGATCAGGTGGAGCGAATTATTGCGGAATTTATGCAACCA<br>TTTGCTCTTGAAGTTGCTCCGTTACTTCGTGTAGGTCTTGTTAAATTGGAGGCAGA<br>ACGTCATCTATTTATAATGGATATGCATCATATCATCTCGGATGGGGTATCCATGCAG

	ATCATGATTCAAGAAATTGCTGATTTGTATAAAGAAAAGGAACTTCCTACGTTAGG<br>CATTCAATATAAAGACTTTACTGTTTGGCATAATCGCTTGCTTCAATCGGATGTTAT<br>TGAAAAACAAGAAGCTTACTGGCTGAACGTATTTGCAGAAGAGATTCCAGTATTG<br>AATCTACCGACCGATTACCCAAGACCAACCATTCAAAGCTTTGATGGTAAAAGAT<br>TTACATTCAGTACAGGAAAGCAGCTTATGGATGATTTATACAAGGTGGCAACAGA<br>AACAGGAACAACACTATATATGGTTTTACTTGCTGCGTATAATGTTTTCTTATCGAA<br>GTATTCCGGGCAAGATGACATCGTTGTAGGAACACCGATTGCTGGTAGGTCCCAT<br>GCTGATGTGGAAAATATGCTGGGGATGTTTGTAAATACATTAGCAATAAGAAGTCG<br>TTTAAATAATGAGGATACTTTTAAAGATTTTTTAGCAAATGTAAAACAAACGGCTT<br>TGCATGCCTATGAAAATCCAGATTACCCATTTGATACGCTTGTCGAAAAGTTGGGT<br>ATACAGAGAGATTTAAGTAGAAATCCATTATTTGATACGATGTTTGTTTTGCAAAA<br>TACGGATAGAAAGTCTTTTGAGGTTGAACAGATAACGATTACACCATATGTTCCAA<br>ATAGCAGACATTCTAAATTTGATCTTACATTAGAGGTTAGCGAAGAACAAAATGA<br>GATTTTATTATGCCTAGAATATTGCACTAAATTATTTACGGATAAAACAGTTGAAAG<br>AATGGCTGGTCATTTTTTACAGATCTTGCATGCAATTGTTGGGAACCCAACGATTA<br>TAATATCAGAAATCGAGATATTGTCTGAAGAAGAAAAACAACATATTTTATTCGAG<br>TTCAACGATACGAAAACCACATATCCACATATGCAAACAATTCAAGGATTATTTGA<br>GGAACAGGTGGAGAAAACGCCCGACCATGTTGCAGTTGGATGGAAAGACCAAA<br>CATTAACGTATCGGGAACTTAACGAAAGAGCGAATCAGGTCGCAAGAGTCTTACG<br>GCAAAAAGGAGTCCAACCCGATAATATCGTGGGATTGCTGGTTGAGCGTTCACCT<br>GAAATGCTCGTGGGTATCATGGGAATTCTTAAAGCAGGGGGAGCTTATTTACCTCT<br>TGATCCGGAGTACCCAGCGGATAGAATTTCGTACATGATACAAGATTGTGGTGTAC<br>GCATTATGCTTACCCAACAGCATCTTTTATCTTTAGTACATGATGAATTTGATTGTG<br>TTATTTTGGATGAGGACAGTTTGTACAAGGGGGATTCTTCCAATTTGGCTCCGGTT<br>AACCAGGCCGGGGATTTAGCCTACATCATGTACACTTCTGGTTCTACAGGAAAGC<br>CTAAAGGTGTTATGGTAGAACATCGAAATGTGATTCGCCTTGTGAAAAATACAAA<br>TTATGTTCAGGTCCGCGAGGACGATCGTATAATACAGACCGGAGCAATTGGATTC<br>GATGCACTGACATTTGAAGTTTTTGGCTCATTGCTGCATGGAGCTGAATTGTATCC<br>TGTTACTAAAGACGTGCTATTAGATGCAGAGAAACTACACAAATTTTTACAAGCG<br>AATCAAATTACGATTATGTGGTTAACTTCTCCGTTATTTAACCAATTGTCACAAGG<br>AACCGAAGAGATGTTTGCTGGCCTTCGCTCCCTAATTGTAGGTGGAGATGCCTTG<br>TCTCCGAAACACATCAATAATGTAAAGCGAAAATGCCCTAATCTGACTATGTGGA<br>ACGGTTACGGCCCAACAGAAAACACCACTTTTTCTACATGCTTTCTTATTGATAAA<br>GAATATGATGACAATATTCCGATAGGGAAGGCCATTAGTAATTCAACAGTGTATAT<br>CATGGACCGGTATGGCCAGCTTCAGCCGGTGGGTGTACCAGGAGAATTATGTGTA<br>GGAGGGGATGGGGTTGCCAGGGGATATATGAATCAGCCTGCATTAACAGAAGAG<br>AAGTTTGTCCCAAATCCATTCGCTCCTGGTGAGAGAATGTATCGCACGGGGGATT<br>TGGCAAGATGGTTGCCTGATGGAACAATTGAGTATTTAGGTCGTATTGATCAGCA<br>AGTGAAAATCAGGGGCTACCGTATTGAACCGGGAGAGATTGAAACGCTTCTTGT<br>GAAGCACAAAAAAGTCAAAGAATCGGTAATCATGGTAGTAGAGGATAATAATGG<br>ACAAAAGGCTCTATGCGCTTATTACGTTCCGGAAGAAGAAGTAACGGTATCTGAA<br>CTGAGGGAATATATAGCTAAAGAGTTGCCTGTTTACATGGTTCCAGCCTATTTTGT<br>ACAGATTGAACAAATGCCTCTTACACAGAACGGTAAAGTAAATCGAAGCGCGTTA<br>CCAAAACCAGATGGTGAATTTGGTACAGCAACCGAATATGTAGCGCCTAGCAGCG<br>ACATTGAAATGAAGCTGGCAGAGATTTGGCATAATGTGTTAGGGGTAAACAAAAT<br>CGGGGTACTGGATAACTTCTTTGAATTAGGTGGTCATTCATTAAGAGCTATGACAA<br>TGATTTCCCAGGTACATAAAGAGTTCGACGTTGAATTGCCATTAAAAGTGTTATTT<br>GAAACACCAACGATCTCTGCATTAGCTCAATACATTGCTGATGGAGAAAAAGGAA<br>TGTACCTGGCCATTCAACCTGTTACCCCGCAGGATTACTATCCAGTATCATCTGCG<br>CAAAAGAGGATGTACATCCTTTATGAATTTGAAGGGGCTGGCATTACCTATAATGT<br>ACCTAATGTAATGTTTATAGAAGGAAAGCTGGATTATCAGCGCTTTGAATACGCTA<br>TAAAAAGTTTGGTAAATCGACATGAGGCGCTTCGAACGTCTTTCTATTCGCTTAAT<br>GGAGAACCAGTTCAGCGTGTACATCAAAATGTAGAGCTACAGATTGCTTATTCGG<br>AGGCGAAAGAAGATGAGATAGAGCAAATTGTAGAAAGCTTTGTTCAACCATTTG<br>ACCTTGAAATAGCTCCGCTGCTTCGCGTAGGGCTTGTTAAATTGGCATCGGATCGC<br>TATTTATTCCTAATGGATATGCATCATATTATCTCAGATGGTGTATCAATGCAAATTA<br>TAACAAAAGAAATTGCCGACTTATATAAAGGAAAAGAGCTTGCTGAACTGCATAT

	TCAGTATAAAGATTTTGCTGTATGGCAAAACGAATGGTTTCAATCTGACGCTCTTG<br>AAAAACAGAAAACGTATTGGTTGAACACCTTTGCAGAGGATATTCCGGTTTTAAA<br>TTTGTCAACTGATTATCCAAGACCGACAATTCAAAGTTTTGAAGGAGATATTGTCA<br>CGTTTAGTGCAGGGAAGCAACTTGCGGAAGAATTGAAACGCCTGGCTGCAGAAA<br>CAGGGACGACTTTGTATATGCTTCTGTTAGCGGCGTACAATGTACTTTTACACAAA<br>TACTCGGGACAGGAAGAAATTGTAGTAGGAACGCCTATTGCCGGGCGATCTCACG<br>CAGATGTGGAAAATATTGTTGGGATGTTTGTCAATACGCTTGCATTGAAAAATACC<br>CCTATAGCCGTACGCACCTTCCACGAATTCCTGTTGGAAGTAAAACAAAATGCTT<br>TAGAAGCTTTTGAAAATCAAGACTATCCATTTGAAAATTTGATAGAGAAGCTGCA<br>AGTGCGTCGCGACTTAAGTCGCAATCCATTATTTGATACAATGTTTAGCCTAAGCA<br>ATATTGACGAACAAGTAGAGATAGGGATTGAGGGATTGAACTTCAGCCCATATGA<br>AATGCAGTATTGGATTGCAAAATTTGATATTTCATTCGATATTTTAGAAAAGCAAG<br>ATGACATTCAATTTTATTTTAACTATTGCACGAATCTGTTTAAAAAAGAAACGATA<br>GAACGATTAGCGACACACTTTATGCATATTTTACAGGAGATTGTTATTAATCCTGA<br>GATTAAGTTATGTGAAATTAATATGCTGTCCGAAGAAGAACAGCAGCGTGTCCTG<br>TATGACTTTAATGGCACAGATGCAACCTACGCTACGAATAAAATATTCCATGAGTT<br>ATTTGAAGAACAGGTTGAAAAAACACCAGATCATATAGCGGTGATAGATGAAAG<br>AGAAAAGCTTTCCTATCAGGAGCTTAATGCGAAAGCGAATCAGCTGGCACGAGT<br>GCTGCGCCAAAAAGGAGTACAGCCTAATAGCATGGTAGGTATTATGGTAGATCGC<br>TCACTCGACATGATTGTAGGAATGCTTGGGGTTTTAAAAGCAGGAGGAGCATATG<br>TGCCTATCGATATAGACTATCCTCAGGAACGGATTAGCTACATGATGGAAGATAGT<br>GGTGCAGCGCTCTTGTTAACACAACAAAAGTTGACACAGCAAATTGCGTTTTCTG<br>GTGACATTTTGTATCTTGACCAAGAAGAATGGCTTCATGAGGAAGCTTCAAATTT<br>AGAACCCATCGCTCGTCCGCAGGATATAGCCTATATCATTTACACTTCTGGTACAA<br>CCGGAAAGCCAAAAGGTGTGATGATTGAGCATCAAAGCTATGTGAATGTAGCAAT<br>GGCATGGAAAGATGCCTATCGGTTAGATACATTCCCGGTCCGTTTGCTTCAGATGG<br>CTAGCTTTGCCTTTGACGTATCTGCGGGTGATTTTGCCAGAGCACTACTTACAGGT<br>GGGCAATTAATTGTATGTCCAAATGAAGTAAAGATGGACCCAGCTTCTTTATATGC<br>CATTATTAAGAAATATGACATTACTATTTTTGAAGCAACGCCTGCTCTAGTGATTCC<br>ATTGATGGAGTATATTTATGAACAGAAGCTGGATATTAGCCAGTTACAGATTCTGAT<br>TGTCGGATCGGACAGTTGTTCGATGGAGGACTTTAAAACCTTGGTTTCCCGTTTT<br>GGTTCAACTATACGTATTGTGAATAGCTATGGAGTAACCGAAGCGTGCATTGATTC<br>TAGCTATTATGAACAACCGCTTTCTTCGTTACATGTAACAGGAACTGTACCGATTG<br>GAAAACCGTACGCTAACATGAAAATGTATATTATGAATCAATATTTGCAGATTCAG<br>CCTGTAGGTGTAATTGGAGAATTATGTATTGGAGGAGCCGGGGTTGCCCGTGGAT<br>ATTTAAATAGACCGGACTTAACAGCAGAAAAGTTTGTCCCTAATCCTTTTGTTCCA<br>GGTGAAAAGCTGTATCGAACAGGCGACTTGGCAAGATGGATGCCGGATGGGAAT<br>GTTGAGTTTCTTGGTCGAAATGACCATCAGGTGAAAATCAGAGGGATTCGAATCG<br>AGCTTGGAGAAATCGAAGCACAACTGCGTAAACATGATAGCATAAAAGAAGCAA<br>CTGTGATCGCAAGAGAAGATCACATGAAAGAGAAATATTTATGTGCGTATATGGTG<br>ACCGAAGGAGAAGTAAATGTAGCTGAACTGCGTGCGTATCTAGCAAATGATCTGC<br>CTGCGGCAATGATTCCGTCATATTTTGTATCGCTCGAAGCAATGCCACTTACTGCT<br>AATGGAAAAATTGATAAGCGATCTTTACCAGAGCCCGATGGTTCCATATCGATAGG<br>AACAGAATATGTAGCTCCGCGTACCATGCTTGAGGGAAAACTAGAAGAGATATGG<br>AAAGATGTATTGGGTTTACAGCGTGTTGGCATTCACGATGACTTCTTTACAATAGG<br>TGGCCATTCATTGAAGGCTATGGCTGTTATTTCGCAAGTTCATAAAGAATGCCAGA<br>CTGAAGTTCCTCTGCGTGTCTTATTTGAAACACCTACCATTCAAGGACTGGCTAA<br>ATATATAGAGGAAACGGACACAGAGCAATATATGGCTATTCAGCCGGTTAGCGGA<br>CAGGACTATTATCCAGTATCATCAGCACAAAAGAGAATGTTTATTGTTAATCAATT<br>TGATGGAGTAGGAATTAGCTACAATATGCCTTCCATCATGCTGATTGAAGGAAAAC<br>TTGAGCGAACACGCTTGGAATCAGCATTTAAAAGATTGATAGAACGACATGAGAG<br>CCTTCGAACATCTTTTGAAATAATAAATGGTAAGCCTGTACAGAAGATTCATGAGG<br>AAGTTGATTTCAATATGTCCTATCAGGTGGCTTCTAATGAACAAGTAGAGAAGATG<br>ATCGATGAGTTCATTCAGCCTTTCGATTTAAGTGTTGCACCGCTGCTTCGTGTGGA<br>ACTTTTAAAATTGGAAGAGGACCGTCATGTGCTTATATTTGATATGCATCATATTAT<br>CTCAGATGGTATATCTTCCAATATTTTGATGAAAGAATTAGGAGAACTATATCAAG<br>GTAATGCTTTACCAGAACTTCGTATTCAATACAAGGATTTCGCTGTATGGCAAAAT

	GAGTGGTTCCAGTCAGAAGCCTTTAAAAAGCAAGAAGAATACTGGGTAAATGTT<br>TTCGCAGATGAACGCCCGATTCTGGATATACCGACGGATTATCCAAGGCCGATGC<br>AACAAAGCTTTGATGGTGCTCAACTTACATTTGGAACCGGAAAGCAGCTTATGGA<br>TGGGTTATACAGGGTAGCAACGGAAACGGGAACAACGCTTTATATGGTTTTGCTT<br>GCGGCATATAATGTTCTTCTTTCCAAATATTCTGGTCAAGAAGATATTATTGTAGGG<br>ACACCGATTGTGGGTAGATCCCATACTGACCTTGAGAATATTGTCGGGATGTTTGT<br>CAACACGTTAGCAATGAGAAATAAACCGGAAGGAGAAAAGACGTTCAAAGCATT<br>TGTATCAGAAATAAAGCAGAATGCACTAGCGGCTTTTGAGAATCAGGATTATCCAT<br>TTGAGGAGCTTATCGAAAAACTAGAGATACAAAGGGACTTAAGCAGAAATCCATT<br>ATTTGATACGCTCTTTAGCCTTCAAAACATAGGTGAAGAATCATTTGAACTAGCCG<br>AATTAACATGCAAACCTTTCGATTTGGTAAGCAAATTAGAGCATGCCAAGTTTGAT<br>CTGAGTCTTGTGGCAGTAGAAAAAGAGGAAGAAATTGCATTTGGGCTTCAATACT<br>GCACAAAACTGTATAAGGAAAAAACAGTTGAACAACTGGCTCAACATTTTATTCA<br>AATAGTAAAAGCAATTGTAGAAAATCCAGATGTCAAATTATCTGATATTGATATGTT<br>ATCTGAAGAAGAGAAGAAACAAATCATGCTTGAGTTCAATGATACGAAAATACA<br>ATATCCGCAGAATCAAACAATACAGGAATTGTTTGAGGAGCAAGTGAAGAAAAC<br>ACCTGAACATATAGCAATCGTATGGGAAGGGCAAGCATTAACCTATCATGAGCTAA<br>ATATAAAAGCTAATCAGTTAGCTCGTGTATTACGAGAAAAAGGGGTAACCCCTAAT<br>CATCCTGTAGCGATTATGACGGAACGCTCATTAGAGATGATCGTAGGTATCTTTAG<br>TATTTTGAAAGCAGGAGGAGCATATGTTCCAATTGATCCAGCCTATCCACAAGAA<br>CGTATTCAATACTTGCTTGAAGATAGCGGAGCGACGCTACTGCTTACTCAGTCAC<br>ATGTATTAAATAAATTACCGGTCGATATCGAATGGTTGGATCTTACAGATGAACAA<br>AACTATGTAGAAGATGGTACCAATCTTCCATTTATGAATCAGTCAACAGATCTTGC<br>CTATATTATTTATACATCCGGTACAACAGGCAAGCCTAAAGGGGTTATGATTGAAC<br>ATCAAAGCATCATCAACTGCCTGCAATGGCGGAAGGAAGAATACGAATTTGGACC<br>AGGGGATACGGCTCTACAAGTGTTTTCCTTTGCTTTTGATGGATTTGTAGCAAGTT<br>TGTTTGCTCCGATTCTTGCTGGTGCAACGTCTGTTCTCCCTAAGGAGGAAGAAGC<br>AAAAGATCCAGTTGCATTGAAAAAACTGATCGCATCAGAAGAGATTACACATTAC<br>TACGGTGTGCCTAGTTTGTTTAGTGCCATTCTTGATGTTTCTTCTAGTAAGGATTTG<br>CAAAATTTACGCTGCGTCACTTTGGGAGGAGAGAAATTACCGGCTCAAATTGTTA<br>AAAAAATCAAAGAAAAAAATAAAGAAATTGAAGTCAACAACGAATATGGGCCTA<br>CTGAAAATAGTGTAGTAACTACTATTATGCGCGATATACAGGTAGAACAAGAGATT<br>ACTATTGGTCGCCCATTATCTAACGTAGATGTATATATTGTCAATTGTAATCATCAAT<br>TACAACCAGTAGGTGTAGTAGGGGAATTATGTATTGGTGGACAGGGACTTGCAAG<br>AGGATATTTGAATAAACCAGAGCTTACAGCAGATAAATTTGTTGTAAATCCATTCG<br>TACCTGGTGAACGTATGTACAAAACCGGTGACCTTGCAAAATGGCGCTCAGATGG<br>AATGATTGAATATGTGGGGCGTGTTGATGAACAAGTAAAAGTAAGAGGATATCGG<br>ATTGAGCTTGGTGAAATTGAATCAGCTATCCTAGAATACGAAAAAATTAAGGAAG<br>CGGTAGTTATGGTTTCGGAGCATACTGCATCTGAACAGATGTTATGTGCTTATATTG<br>TAGGGGAAGAAGATGTACTGACTCTGGACTTAAGAAGCTATCTAGCAAAATTACT<br>ACCAAGTTATATGATTCCAAACTATTTTATCCAATTGGATAGTATTCCGCTTACACC<br>AAACGGTAAAGTGGATCGTAAAGCATTGCCTGAACCTCAAACCATTGGCTTAATG<br>GCAAGGGAGTATGTTGCACCAAGGAATGAAATCGAAGCACAGCTAGTACTCATTT<br>GGCAAGAGGTATTAGGAATAGAACTGATCGGTATTACCGATAATTTCTTTGAATTA<br>GGAGGGCATTCTTTAAAGGCAACGCTTTTAGTTGCAAAAATTTACGAGTACATGC<br>AAATAGAGATGCCATTAAATGTTGTGTTTAAACATTCAACTATTATGAAAATAGCG<br>GAATATATTACACATCAAGAATCAGAAAATAATGTACATCAGCCTATTTTGGTAAAT<br>GTAGAAGCAGATAGAGAGGCGCTATCTCTTAACGGCGAGAAGCAAAGAAAAAAT<br>ATAGAGCTACCTATTCTGCTAAACGAAGAAACAGATCGAAACGTATTCTGCTTCG<br>CGCCCATTGGTGCACAAGGTGTTTTTTATAAAAAGCTTGCTGAACAAATCCCTAC<br>TGCATCCTTGTATGGCTTTGACTTCATTGAAGATGATGATCGAATTCAGCAATATAT<br>TGAATCGATGATTCAAACTCAGTCAGACGGACAATATGTGCTAATTGGTTATTCTT<br>CAGGAGGGAACCTGGCTTTTGAAGTAGCAAAAGAAATGGAAAGGCAAGGATATA<br>GTGTATCTGATTTGGTCTTGTTCGATGTTTACTGGAAGGGAAAAGTATTCGAGCAA<br>ACAAAAGAAGAAGAAGAAGAAAACATAAAAATAATAATGGAAGAATTAAGGGA<br>AAATCCAGGAATGTTCAATATGACACGAGAGGATTTTGAACTGTATTTTGCGAATG<br>AATTTGTGAAACAAAGTTTCACACGGAAAATGCGCAAATACATGAGTTTTTATAC

	GCAGTTAGTTAATTATGGGGAAGTAGAAGCTACAATTCACCTTATACAAGCAGAAT<br>TTGAGGAAGAAAAAATTGACGAAAACGAAAAAGCCGACGAAGAAGAAAAAAC<br>ATATCTAGAGGAAAAATGGAATGAAAAAGCATGGAACAAAGCAGCAAAAAGATT<br>TGTAAAATATAACGGATATGGCGCTCATTCTAACATGCTAGGAGGTGATGGTTTAG<br>AGAGAAATTCCTCTATCCTTAAACAGATACTACAAGGGACATTTGTAGTAAAATAA<br>AAGAAGAAGTGTGAAAAAGCGCAGCTGAAATAGCTGCGCTTTTTTGTGTCATAA
pGETS151<br>P2nd-B2	AAAAGGCCTTCTTGGCCGCCCTTCCCGGTCGATATGAACAGCTTATTTACATAATT<br>CACGTTATTGGTAGTTATAAATGAAATTCCTAATATCGGTTATGAAGTGAAATTGAA<br>TTTCTACTTGATCTTTCTCTCTATTTTTGTAAAATAAAATTAAGAATATTTAAATATT<br>CAATGATTCATTTTTGCAGAAATCGGAGGAAGAAGAATATATGAAAACATTTAAC<br>ATTTCTCAACAAGATCCCCCCATATTGTTGTATAAGTGATGAAATAC**C**GAATTTAA<br>AACCTAGTTTATATGTGGTAAAATGTTTTAATCAAGTTTAGGAGGAATTAATTATGA<br>AGTGTAATGAATAATGAGTGTAACAGGGTTCAATTAAAAGAGGGAAGCGTATCAT<br>TAACCCTATAAACTACGTCTGCCCTCATTATTGGAGGGTGAAATGTGAATACATCC<br>TATTCACAATCGAATTTACGACACAACCAAATTTTAATTTGGCTTTGCATTTTATCT<br>TTTTTTAGCGTATTAAATGAAATGGTTTTGAACGTGTCATTACCTGATATTGCAAAT<br>GATTTTAATAAACCACCAGCGAGTACAAACTGGGTGAACACAGCCTTTATGTTAA<br>CCTTTTCCATTGGAACAGCTGTATATGGAAAGCTATCTGATCAATTAGGCATCAAA<br>AGGTTACTCCTATTTGGAATTATAATAAATTGTTTCGGGTCGGTAATTGGGTTTGTT<br>GGCCATTCTTTCTTTTCCTTACTTATTATGGCTCGTTTTATTCAAGGGGCTGGTGCA<br>GCTGCATTTCCAGCACTCGTAATGGTTGTAGTTGCGCGCTATATTCCAAAGGAAAA<br>TAGGGGTAAAGCATTTGGTCTTATTGGATCGATAGTAGCCATGGGAGAAGGAGTC<br>GGTCCAGCGATTGGTGGAATGATAGCCCATTATATTCATTGGTCCTATCTTCTACTC<br>ATTCCTATGATAACAATTATCACTGTTCCGTTTCTTATGAAATTATTAAAGAAAGAA<br>GTAAGGATAAAAGGTCATTTTGATATCAAAGGAATTATACTAATGTCTGTAGGCAT<br>TGTATTTTTTATGTTGTTTACAACATCATATAGCATTTCTTTTCTTATCGTTAGCGTG<br>CTGTCATTCCTGATATTTGTAAAACATATCAGGAAAGTAACAGATCCTTTTGTTGAT<br>CCCGGATTAGGGAAAAATATACCTTTTATGATTGGAGTTCTTTGTGGGGGAATTAT<br>ATTTG**A**AACAGTAGCAGGGTTTGTCTCTATGGTTCCTTATATGATGAAAGATGTTC<br>ACCAGCTAGGTACTGCCGAAATCGGAAGTGTAATTATTTTCCCTGGAACAATGAG<br>TGTCATTATTTTCGGCTACATTGGTGGGATACTTGTTGATAGAAGAGGTCCTTTATA<br>CGTGTTAAACATCGGAGTTACATTTCTTTCTGTTAGCTTTTTAACTGCTTCCTTTCT<br>TTTAGAAACAACATCATGGTTCATGACAATTATAATCGTATTTGTTTTAGGTGGGCT<br>TTCGTTCACCAAAACAGTTATATCAACAATTGTTTCAAGTAGCTTGAAACAGCAG<br>GAAGCTGGTGCTGGAATGAGTTTGCTTAACTTTACCAGCTTTTTATCAGAGGGAA<br>CAGGTATTGCAATTGTAGGTGGTTTATTATCCATACCCTTACTTGATCAAAGGTTGT<br>TACCTATGGAAGTTGATCAGTCAACTTATCTGTATAGTAATTTGTTATTACTTTTTTC<br>AGGAATCATTGTCATTAGTTGGCTGGTTACCTTGAATGTATATAAACATTCTCAAA<br>GGGATTTCTAAATCGTTAAGGGATCAACTTTGGGAGAGAGTTCAAAATTGATCCT<br>TTTTTTATAACAGGAATTGGGCATCAAATAAAACGAAAGACTGGGCCTTTCGTTTT<br>ATCTGTTGTTTGTCGGTGAACGCTCTCCTGAGTAGGACA**G**GTCCGCCGGGAGCG<br>GATTTGAACGTTGCGAAGCAACGGCCCGGAGGGTGGCGGGCAGGACGCCCGCC<br>ATAAACTGCCAGGCATCAAATTAAGCAGAAGGCCATCCTGACGGATGGCCTTTTT<br>GCGTTTCTACAAACTCTTCCTGTCGTCATATCTACAATTCTACACAGCCCAGTCCA<br>GACTATTGAATTGTATCACGGTTTTGATATCCTACCAATAACAAATTGATTGGAGG<br>AATGCAAAGTGAATAATGAACCAGTAAAACGTGGTAAGAAGAACAGATGGGAAT<br>TAAACCTACCTATAATGACTTATGTAGTAGCTGATGATTGGATTGATAAACTAGGAC<br>ACGAAACGTTTACTTTATGGTTGAGGTTCCATACTTGGGTAGATAGAGAAGATGA<br>ACTCCGAGATTATGATCGCATACCTAGAAGTTTTGAGAACATATATAAAAAGACAC<br>TAGGAATCTCAAAAAGTAAGTTTTATAGATTGATAAAACCTTTATGGGAATATGGA<br>TTAATAGACATCATAGAATACGAAGAATCTAACCGTAATTCTACTAAACCTAAAAA<br>TATAATTGTTTATGAGTATCCTTTACACGAAATAGAAAGAAAGTATAAACCACTAG<br>AAAAATTAAGAGATTGGGATAAAGACTATAATTCCGTTTCTAAAGAATTAGGTAAA<br>ACAGGTGGTAGACCAAGGAAAAAAGATAGTGAAGAAGAACCCGAAAAGAAACC<br>CGAAGAAGTAACTAAAAAGAAACGTAAATATAAGTTAAAAAGAGTTATCCACAA<br>CGGTTTCAAAAATGAAACGGTGGAGGGTTTCAAAAATGAAACGGTGGAGGGTTT<br>CAAAAATGAAACGGTGACCGTTTCAAAAATAAAACCCAATAATTATTCAAATATCT

	TTAATAACTTATCAAATATTTCTACTAATGTTTCAAATAATTTATTAATTGATGATGA<br>TGAGGAAATCGAAAATGAACCAACTGGTCGTACAATAAATAGGTCATTACTTTTTT<br>CGCAAGAAGATATTAAACAGGCCTATCAATTTATTAATAGATTTTCAGTTATACAGT<br>TACGTGAAAACTTTAGCTTTGATAAACACTTTGAAGAACGGTTGGTATGTTAT**C**TA<br>TGGAAAGCAGGGATTTCTACTTTTTACACGCACGAAATCAGTAAAATGATAAAAA<br>AAATAGCAGACTATGAAAAATCTAAAAAAGGTAGATTAAACCCAATACGTGACCG<br>AGCCTTATATATGGTAAATGGTCTTGTAATGAATAGAGCTTCTTCCCAAAGTGAAC<br>ATGCTACTTATAAACTAAACCAATATAAAAAACAGAAGGAACAGGAAAAACAAC<br>AACAGGAGCAACAAAGATCAAGAGTACCGTTCTATAATTGGTTGGAGGAAAGAG<br>AAGAACAAACCGAAGGTCAACTACCCACCACTTAAGCGGCCGCAAGCTTGAAG<br>AGCTCTTCTTTCAGAACGCTCGGTTGCCGCCGGGCGTTTTTTATGAGATGTCTCGG<br>CCTGTTTGGCCATTAATCGAAGAAGAAGTGTGAAAAAGCGCAGCTGAAATAGCT<br>GCGCTTTTTTGTGTCATAATCCTTCGATATATCGCGTCTATTCCGGCTTCCGGCTAT<br>CACCCGAAGATAAACAGCCCAGGGGTCACAGATGAAGTACTGAAGAAAATGAGG<br>AACGGTTTGATTAAGGTAAGGCCGTATACAGTCAATCGTCCGGAAGATATGAAGC<br>GTCTCATTGAAGCGGGAGCAGACGGCATGTTTACCGACTTTCCAGAAAAGGCTTC<br>GGCATTGCTGAAAAATGAATAGTTGTTAGAAGGAGGCTGTTTGACGCAGCCTTCT<br>TTTTTCATTCATTCATGCCCGTTTCAAAGCATACATTCATAGAAGACGGAAGAATA<br>AAGGGAGAGGTGAATGTGACTTTTATTTCACAAGTAAATAAATGGTTTGTTAATGC<br>TAATGTTAACTCAGCTGCAAAGCTTAGGCTATTCTGTATTCCATATGCAGGCGGTG<br>GTGCTTCCGCCTTTTATGAATGGAGTCATTTTTTTCCAAAGGAAATTGAAGTTTGT<br>TCAATTCAATTACCTGGAAGGGAAAATAGGGGGGCGGAAGTTCCGCTAACAAATT<br>TACAACAGATAGTAGAAATAGTAGCTGAGGAAATACAACCATTAATAAATATTCCA<br>TTTGCTTTTTTGGGGCATAGCATGGGAGCATTAATAAGTTTTGAACTGGCTCGCAC<br>AATACGGCAAAAGAGTAATGTTAATCCGGTTCACTTGTTTGTTTCAGGGCGACAT<br>GCACCTCAAATCCCATGTGCAAAACAAGACTATCATTTACTTCCCGATGAACAATT<br>TATACAAGAATTGCGTTCATTGAATGGAACTCCAGAGATAGTATTACAAGACGCA<br>GAGATGATGAGTATATTACTCCCAAGACTTCGGGCTGATTTTTCTGTGTGTGGCTC<br>CTATCAGTACAAAAACGACGAGCCTTTTGAATGCCCAATCACTGCTTTTGGAGGA<br>AAAAATGATAATGGTGTTACTTATCAATCATTAGAAGCCTGGAGAGAGCAAACCA<br>AGAGGGAATTTTCTGTGTGTATGTATCCAGGTGATCATTTTTTTCTTTACGAAAGC<br>AAATATGAAATGATTGAGTTCATGTGTAAACAATTACGTTTAGTATTAGCTCCTAA<br>AATATAAGGCCTTGATGGCCATCGAAGAAGAAGTGTGAAAAAGCGCAGCTGAAA<br>TAGCTGCGCTTTTTTGTGTCATAATCCTTATTGAGTGGATGATTATATTCCTTTTGAT<br>AGGTGGTATGTTTTCGCTTGAACTTTTAAATACAGCCATTGAACATACGGTTGATT<br>TAATAACTGACAAACATCACCCTCTTGCTAAAGCGGCCAAGGACGCTGCCGCCG<br>GGGCTGTTTGCGTTTTTGCCGTGATTTCGTGTATCATTGGTTTACTTATTTTTTTGC<br>CAAAGCTGTAATGGCTGAAAATTCTTACATTTATTTTACATTTTTAGAAATGGGCG<br>TGAAAAAAAGCGCGCGATTATGTAAAATATAAAGTGATAGCGGTACCGGAACTAT<br>TTTTTACAGGGGGTATATATGTTAAACAG**C**TCTAAAAGTATATTGATTC**G**TGCTCAA<br>AATAAAAATGGAACGCATGAAGAGGAGCAGTATCTCTTTGCTGTGAACAACACC<br>AAAGCGGAGTATCCACGTGATAAGACGATCCATCAGTTATTTGAGGAGCAGGTTA<br>GTAAGAGGCCAAACAATGTAGCCATTGTATGTGAAAATGAGCAACTTACCTACCA<br>TGAGCTTAATGTGAAAGCCAATCAACTAGCACGGATTTTTATAGAAAAAGGGATT<br>GGAAAAGACACTCTTGTTGGAATTATGATGGAGAAATCTATCGATTTATTTATAGG<br>CATATTAGCCGTTTTAAAAGCTGGTGGAGCATATGTTCCGATTGATATTGAATATCC<br>TAAGGAAAGAATTCAATATATTCTTGATGATAGTCAGGCAAGAATGCTACTTACCC<br>AGAAGCATTTGGTTCATTTAATTCATAATATTCAATTTAATGGGCAAGTGGAAATTT<br>TTGAAGAAGATACTATCAAAATTAGAGAAGGAACTAATCTACATGTACCAAGTAA<br>ATCAACCGATCTTGCTTATGTTATTTATACTTCTGGTACAACAGGCAATCCAAAAG<br>GTACAATGCTGGAGCATAAAGGAATAAGTAATCTAAAGGTATTTTTCGAAAATAGT<br>CTTAACGTGACTGAAAAGGATAGAATTGGTCAATTTGCCAGCATCTCTTTTGATGC<br>ATCTGTATGGGAGATGTTTATGGCTTTGTTAACGGGGGCTAGCCTGTATATTATCCT<br>GAAGGATACAATCAATGATTTTGTGAAGTTTGAACAATACATTAACCAAAAGGAA<br>ATCACTGTTATTACGTTACCACCTACCTATGTAGTTCATCTTGATCCAGAACGTATT<br>TTATCGATACAAACGTTAATTACAGCAGGCTCAGCTACCTCGCCTTCCTTAGTAAA<br>CAAG**A**GGAAGGAGAAAGTAACTTACATAAATGCCTATGGCCCTACGGAAACAAC

	TATTTGTGCGACTACATGGGTAGCCACCAAAGAAACAATAGGTCATTCAGTTCCA<br>ATCGGAGCACCAATTCAAAATACACAAATTTATATTGTCGATGAAAATCTTCAATT<br>AAAATCGGTTGGTGAAGCTGGTGAATTGTGTATTGGTGGAGAAGGGTTAGCAAG<br>GGGATATTGGAAGCGACCGGAATTAACTTCCCAGAAGTTCGTTGATAACCCGTTT<br>GTTCCAGGAGAGAAGTTGTATAAAACAGGAGATCAGGCAAGATGGCTATCTGATG<br>GAAATATTGAATATCTCGGAAGAATAGATAACCAGGTAAAGATTAGAGGTCACCG<br>AGTTGAACTAGAAGAAGTTGAGTCTATTCTTCTAAAGCATATGTATATTAGCGAAA<br>CTGCAGTAAGTGTGCATAAAGATCACCAAGAACAGCCGTATTTGTGCGCTTATTTT<br>GTATCGGAAAAGCATATACCACTAGAACAGTTAAGACAATTCTCATCAGAAGAAC<br>TGCCAACGTATATGATCCCTTCTTATTTTATCCAGTTAGACAAAATGCCGCTTACAT<br>CAAATGGGAAGATTGATCGAAAGCAGTTGCCGGAACCTGATTTAACTTTCGGGAT<br>GAGGGTAGACTATGAAGCGCCGCGAAATGAAATCGAGGAAACGCTTGTTACTATC<br>TGGCAGGATGTATTAGGTATTGAGAAAATCGGTATTAAAGATAATTTCTATGCATTA<br>GGTGGAGATTCTATTAAAGCAATACAGGTTGCTGCTCGCCTGCATTCCTACCAATT<br>AAAGCTAGAAACAAAAGATTTATTAAAGTATCCAACAATCGATCAACTCGTTCATT<br>ATATAAAAGATAGTAAAAGAAGAAGTGAGCAAGGTATTGTGGAAGGTGAGATTG<br>GACTTACACCTATTCAGCATTGGTTCTTTGAACAACAATTTACAAATATGCACCAT<br>TGGAACCAATCGTATATGTTGTATAGACCAAATGGGTTTGATAAAGAGATCTTGCT<br>AAGGGTATTTAATAAAATTGTTGAGCATCATGATGCATTACGTATGATATACAAACA<br>TCATAACGGAAAGATCGTGCAGATAAATCGGGGGCTTGAAGGTACGTTGTTTGAT<br>TTTTATACCTTTGATTTAACTGCAAATGATAATGAGCAACAGGTGATTTGTGAAGA<br>ATCTGCTCGATTACAAAATAGTATAAACTTGGAAGTAGGCCCTCTAGTAAAGATAG<br>CGCTGTTTCATACTCAGAATGGAGATCACCTGTTTATGGCTATTCATCATTTGGTTG<br>TGGATGGTATTTCTTGGAGGATTTTGTTTGAGGATTTGGCCACAGCTTATGAACAA<br>GCAATGCATCAGCAAACGATTGCTTTACCAGAGAAAACAGATTCATTTAAGGACT<br>GGTCTATTGAATTAGAAAAATATGCGAACAGCGAATTATTCCTAGAAGAAGCTGA<br>ATATTGGCATCATTTGAATTATTATACCGAGAACGTTCAAATTAAGAAAGATTATGT<br>CACCATGAACAATAAACAAAAGAATATACGTTATGTAGGAATGGAGTTAACAATA<br>GAAGAGACAGAAAAATTATTGAAAAATGTAAATAAAGCGTATCGAACAGAAATTA<br>ATGATATTTTATTAACGGCACTTGGCTTTGCACTCAAAGAATGGGCCGATATTGAT<br>AAAATTGTAATTAACTTAGAGGGACACGGACGGGAAGAAATACTGGAACAGATG<br>AACATTGCAAGGACGGTAGGCTGGTTTACTTCCCAGTATCCTGTTGTACTTGATAT<br>GCAAAAATCGGATGATTTGTCTTATCAAATCAAATTAATGAAAGAAAATTTACGCA<br>GAATACCTAACAAAGGAATCGGATATGAAATTTTTAAGTATTTAACAACTGAATAT<br>TTACGGCCTGTTTTACCCTTTACATTAAAGCCGGAAATTAACTTTAACTACTTAGG<br>ACAGTTCGATACGGACGTGAAAACTGAATTGTTTACTCGTTCTCCTTATAGCATGG<br>GTAATTCATTAGGACCAGATGGAAAAAATAATTTAAGCCCAGAAGGGGAAAGTTA<br>TTTTGTACTCAATATTAATGGTTTTATTGAAGAAGGTAAGCTTCACATCACCTTTTC<br>TTATAATGAACAGCAGTATAAGGAGGATACCATTCAGCAATTGAGCCGGAGCTATA<br>AGCAACATCTTTTGGCCATCATTGAACATTGTGTACAGAAGGAAGATACTGAGTT<br>AACTCCAAGTGATTTCAGTTTCAAGGAACTTGAATTAGAAGAGATGGATGATATT<br>TTCGATTTGTTGGCCGATTCATTAACGTAAGGCCTCGATGGCCATCGAAGAAGAA<br>GTGTGAAAAAGCGCAGCTGAAATAGCTGCGCTTTTTTGTGTCATAATCCTTATGG<br>GAAGTGCTCCGTAATACGCTGACAAGAGAGAAAGGGCTTGGAGGTATTGAAACA<br>AGAGGAGTTCTGAGAATTGGTATGCCTTATAAGTCCAATTAACAGTTGAAAACCT<br>GCATAGGAGAGCTATGCGGGTTTTTTATTTTACATAATGATACATAATTTACCGAAA<br>CTTGCGGAACATAATTGAGGAATCATAGAATTTTGTCAAAATAATTTTATTGACAA<br>CGTCTTATTAACGTTGATATAATTTAAATTTTATTTGACAAAAATGGGCTCGTGTTG<br>TACAATAAATGTAGTGGGAACATTATTATGAGGTGCTAGCATGAGTACATTTAAAA<br>AAGAACATGTTCAGGATATGTATCGTTTATCTCCCATGCAGGAAGGCATGTTGTTT<br>CACGCATTACTTGATAAAGATAAAAATGCTCACCTGGTACAAATGTCTATCGCGAT<br>CGAAGGTATCGTGGATGTGGAGCTGCTTAGTGAAAGCTTGAACATATTGATTGATA<br>GATACGATGTGTTTAGAACAACATTCTTACATGAAAAAATTAAACAACCGCTTCA<br>GGTAGTGCTAAAGGAACGGCCTGTTCAGCTTCAATTTAAAGACATATCATCCTTAG<br>ATGAAGAAAAAAGAGAACAGGCTATTGAGCAGTATAAGTATCAAGATGGGGAAA<br>CAGTCTTTGATTTAACAAGAGATCCCTTGATGAGAGTAGCTATTTTTCAAACTGGT<br>AAGGTTAACTACCAAATGATCTGGAGCTTCCACCATATTTTAATGGATGGTTGGTG

	CTTCAACATTATATTTAATGACTTGTTCAATATATATCTGTCATTAAAAGAGAAGAA<br>ACCTCTTCAGTTAGAGGCGGTGCAACCATATAAGCAGTTTATTAAGTGGCTTGAA<br>AAACAAGATAAACAGGAAGCACTTCGCTACTGGAAAGAACATTTAATGAATTATG<br>ATCAATCAGTAACATTACCTAAAAAGAAAGCAGCTATTAATAATACTACATATGAA<br>CCAGCACAGTTTCGTTTTGCGTTTGACAAAGTGCTTACCCAGCAGCTGCTTCGTA<br>TTGCCAATCAAAGCCAAGTAACACTAAATATTGTTTTTCAAACAATATGGGGGATT<br>GTACTTCAAAAATACAATTCCACTAATGATGTTGTATATGGCTCTGTTGTATCAGGC<br>CGTCCTTCTGAAATATCGGGAATTGAGAAAATGGTTGGACTATTTATTAATACTCTT<br>CCATTACGTATCCAAACGCAAAAAGATCAATCATTTATTGAATTAGTAAAGACTGT<br>TCATCAAAACGTCCTTTTCTCGCAACAGCATGAGTATTTTCCATTGTATGAAATAC<br>AAAATCATACAGAATTAAAACAGAATCTGATTGATCATATTATGGTAATTGAAAAT<br>TATCCTTTAGTAGAAGAATTGCAAAAGAATAGTATCATGCAAAAAGTAGGGTTTAC<br>AGTTCGTGATGTCAAAATGTTTGAACCAACTAATTATGATATGACAGTTATGGTTT<br>TACCTCGTGATGAAATTAGTGTCCGACTCGATTATAACGCAGCCGTTTATGATATAG<br>ATTTCATAAAAAAAATTGAAGGTCACATGAAAGAAGTGGCTTTATGCGTGGCAAA<br>TAATCCACATGTGTTAGTACAGGACGTTCCTCTGCTTACAAAGCAAGAAAAACAA<br>CATTTATTGGTAGAGCTGCATGATTCGATAACAGAGTATCCTGATAAGACGATTCA<br>TCAGTTATTTACAGAACAGGTAGAAAAAACACCAGAGCATGTGGCAGTTGTATTC<br>GAAGATGAGAAAGTGACCTATAGAGAGCTGCATGAGAGATCTAATCAATTAGCCA<br>GATTCTTAAGAGAAAAAGGCGTAAAAAAAGAAAGCATCATAGGCATTATGATGGA<br>GCGTTCAGTTGAAATGATTGTTGGGATCTTAGGGATTTTAAAAGCTGGTGGAGCT<br>TTTGTGCCTATTGATCCTGAATATCCAAAAGAAAGAATCGGCTATATGTTAGATTCT<br>GTACGGCTAGTACTTACACAACGCCATTTAAAGGATAAATTTGCTTTTACGAAAGA<br>AACGATAGTAATTGAAGATCCAAGTATTTCACACGAGTTAACTGAAGAAATAGAT<br>TATATTAATGAATCAGAGGACTTGTTTTATATTATTTATACATCAGGAACAACAGGT<br>AAACCAAAAGGGGTTATGCTAGAGCACAAAAACATCGTTAATCTGCTTCATTTTA<br>CTTTCGAGAAAACAAATATCAACTTTAGTGACAAAGTATTACAGTATACAACATGC<br>AGTTTTGACGTGTGTTACCAAGAAATTTTTTCGACGCTCTTGTCTGGAGGGCAAT<br>TATATCTTATTAGGAAAGAAACTCAACGCGATGTAGAGCAATTATTTGATTTAGTA<br>AAACGTGAAAATATTGAAGTATTATCCTTTCCTGTGGCTTTTCTAAAATTTATTTTC<br>AATGAAAGAGAATTTATCAATCGTTTTCCAACTTGCGTGAAACATATTATCACAGC<br>AGGAGAACAATTAGTAGTTAACAATGAGTTTAAACGTTATTTGCATGAACATAAC<br>GTACATTTACACAATCATTATGGTCCATCAGAAACGCATGTTGTTACCACCTATACT<br>ATTAATCCTGAAGCTGAAATTCCTGAATTACCACCGATAGGAAAACCTATCTCCAA<br>TACATGGATTTATATTTTGGATCAAGAACAACAACTACAACCACAAGGAATTGTAG<br>GAGAGTTATATATTTCGGGCGCAAATGTTGGAAGAGGATATTTGAATAATCAAGAA<br>TTAACGGCAGAAAAATTCTTTGCAGATCCCTTTAGGCCAAACGAACGGATGTACC<br>GAACAGGGGATTTAGCAAGGTGGTTGCCAGACGGAAATATCGAATTTTTAGGAA<br>GGGCCGATCATCAGGTGAAAATTAGGGGGCATCGAATAGAGCTTGGTGAGATCGA<br>GGCACAATTATTAAATTGTAAGGGTGTAAAAGAAGCTGTTGTTATCGATAAAGCG<br>GATGATAAAGGCGGAAAATATTTATGTGCCTATGTTGTTATGGAAGTAGAAGTAAA<br>TGACTCTGAGCTTCGAGAATATTTGGGGAAAGCTTTGCCTGATTATATGATCCCGT<br>CGTTCTTTGTTCCGTTGGATCAGCTGCCGCTTACACCAAACGGAAAAATAGACAG<br>**G**AAATCTCTTCCGAATCTAGAGGGGATTGTGAATACAAACGCAAAATATGTAGTA<br>CCTACAAATGAGCTGGAAGAAAAATTGGCTAAAATCTGGGAAGAAGTACTTGGG<br>ATTTCTCAGATCGGTATACAAGACAATTTCTTTTCGTTAGGCGGGCATTCTCTTAA<br>AGCCATTACGCTTATTTCCCGTATGAACAAAGAGTGTAATGTAGACATTCCTCTAC<br>GTTTGTTATTTGAAGCACCAACCATTCAGGAAATCTCTAATTATATAAACGGGGCA<br>AAGAAAGAAAGCTATGTTGCCATTCAGCCTGTACCAGAACAAGAGTACTATCCTG<br>TATCATCAGTTCAAAAAAGAATGTTTATTCTTAATGAATTTGATCGTTCAGGTACG<br>GCCTATAATTTACCTGGTGTTATGTTTCTAGATGGAAAATTGAACTACCGACAATT<br>GGAAGCAGCGGTAAAAAAATTAGTTGAGCGACATGAAGCGCTGCGTACTTCCTT<br>TCATTCAATTAATGGGGAACCAGTTCAGCGGGTGCATCAAAATGTAGAACTGCAG<br>ATTGCTTATTCAGAGTCAACGGAAGATCAGGTGGAGCGAATTATTGCGGAATTTAT<br>GCAACCATTTGCTCTTGAAGTTGCTCCGTTACTTCGTGTAGGTCTTGTTAAATTGG<br>AGGCAGAACGTCATCTATTTATAATGGATATGCATCATATCATCTCGGATGGGGTAT<br>CCATGCAGATCATGATTCAAGAAATTGCTGATTTGTATAAAGAAAAGGAACTTCCT

	ACGTTAGGCATTCAATATAAAGACTTTACTGTTTGGCATAATCGCTTGCTTCAATC<br>GGATGTTATTGAAAAACAAGAAGCTTACTGGCTGAACGTATTTGCAGAAGAGATT<br>CCAGTATTGAATCTACCGACCGATTACCCAAGACCAACCATTCAAAGCTTTGATG<br>GTAAAAGATTTACATTCAGTACAGGAAAGCAGCTTATGGATGATTTATACAAGGTG<br>GCAACAGAAACAGGAACAACACTATATATGGTTTTACTTGCTGCGTATAATGTTTT<br>CTTATCGAAGTATTCCGGGCAAGATGACATCGTTGTAGGAACACCGATTGCTGGT<br>AGGTCCCATGCTGATGTGGAAAATATGCTGGGGATGTTTGTAAATACATTAGCAAT<br>AAGAAGTCGTTTAAATAATGAGGATACTTTTAAAGATTTTTTAGCAAATGTAAAAC<br>AAACGGCTTTGCATGCCTATGAAAATCCAGATTACCCATTTGATACGCTTGTCGAA<br>AAGTTGGGTATACAGAGAGATTTAAGTAGAAATCCATTATTTGATACGATGTTTGT<br>TTTGCAAAATACGGATAGAAAGTCTTTTGAGGTTGAACAGATAACGATTACACCA<br>TATGTTCCAAATAGCAGACATTCTAAATTTGATCTTACATTAGAGGTTAGCGAAGA<br>ACAAAATGAGATTTTATTATGCCTAGAATATTGCACTAAATTATTTACGGATAAAAC<br>AGTTGAAAGAATGGCTGGTCATTTTTTACAGATCTTGCATGCAATTGTTGGGAACC<br>CAACGATTATAATATCAGAAATCGAGATATTGTCTGAAGAAGAAAAACAACATATT<br>TTATTCGAGTTCAACGATACGAAAACCACATATCCACATATGCAAACAATTCAAGG<br>ATTATTTGAGGAACAGGTGGAGAAAACGCCCGACCATGTTGCAGTTGGATGGAA<br>AGACCAAACATTAACGTATCGGGAACTTAACGAAAGAGCGAATCAGGTCGCAAG<br>AGTCTTACGGCAAAAAGGAGTCCAACCCGATAATATCGTGGGATTGCTGGTTGAG<br>CGTTCACCTGAAATGCTCGTGGGTATCATGGGAATTCTTAAAGCAGGGG**A**AGCTT<br>ATTTACCTCTTGATCCGGAGTACCCAGCGGATAGAATTTCGTACATGATACAAGAT<br>TGTGGTGTACGCA**C**TATGCTTACCCAACAGCATCTTTTATCTTTAGTACATGATGA<br>ATTTGATTGTGTTATTTTGGATGAGGACAGTTTGTACAAGGGGGATTCTTCCAATT<br>TGGCTCCGGTTAACCAGGCCGGGGATTTAGCCTACATCATGTACACTTCTGGTTCT<br>ACAGGAAAGCCTAAAGGTGTTATGGTAGAACATCGAAATGTGATTCGCCTTGTGA<br>AAAATACAAATTATGTTCAGGTCCGCGAGGACGATCGTATAATACAGACCGGAGC<br>AATTGGATTCGATGCACTGACATTTGAAGTTTTTGGCTCATTGCTGCATGGAGCTG<br>AATTGTATCCTGTTACTAAAGACGTGCTATTAGATGCAGAGAAACTACACAAATTT<br>TTACAAGCGAATCAAATTACGATTATGTGGTTAACTTCTCCGTTATTTAACCAATTG<br>TCACAAGGAACCGAAGAGATGTTTGCTGGCCTTCGCTCCCTAATTGTAGGTGGAG<br>ATGCCTTGTCTCCGAAACACATCAATAATGTAAAGCGAAAATGCCCTAATCTGACT<br>ATGTGGAACGGTTACGGCCCAACAGAAAACACCACTTTTTCTACATGCTTTCTTAT<br>TGATAAAGAATATGATGACAATATTCCGATAGGGAAGGCCATTAGTAATTCAACAG<br>TGTATATCATGGACCGGTATGGCCAGCTTCAGCCGGTGGGTGTACCAGGAGAATT<br>ATGTGTAGGAGGGGATGGGGTTGCCAGGGGATATATGAATCAGCCTGCATTAACA<br>GAAGAGAAGTTTGTCCCAAATCCATTCGCTCCTGGTGAGAGAATGTATCGCACGG<br>GGGATTTGGCAAGATGGTTGCCTGATGGAACAATTGAGTATTTAGGTCGTATTGAT<br>CAGCAAGTGAAAATCAGGGGCTACCGTATTGAACCGGGAGAGATTGAAACGCTT<br>CTTGTGAAGCACAAAAAAGTCAAAGAATCGGTAATCATGGTAGTAGAGGATAATA<br>ATGGACAAAAGGCTCTATGCGCTTATTACGTTCCGGAAGAAGAAGTAACGGTATC<br>TGAACTGAGGGAATATATAGCTAAAGAGTTGCCTGTTTACATGGTTCCAGCCTATT<br>TTGTACAGATTGAACAAATGCCTCTTACACAGAACGGTAAAGTAAATCGAAGCGC<br>GTTACCAAAACCAGATGGTGAATTTGGTACAGCAACCGAATATGTAGCGCCTAGC<br>AGCGACATTGAAATGAAGCTGGCAGAGATTTGGCATAATGTGTTAGGGGTAAACA<br>AAATCGGGGTACTGGATAACTTCTTTGAATTAGGTGGTCATTCATTAAGAGCTATG<br>ACAATGATTTCCCAGGTACATAAAGAGTTCGACGTTGA<u>**G**</u>TTGCCATTAAAAGTGT<br>TATTTGAAACACCAACGATCTCTGCATTAGCTCAATACATTGCTGATGGAGAAAA<br>AGGAATGTACCTGGCCATTCAACCTGTTACCCCGCAGGATTACTATCCAGTATCAT<br>CTGCGCAAAAGAGGATGTACATCCTTTATGAATTTGAAGGGGCTGGCATTACCTAT<br>AATGTACCTAATGTAATGTTTATAGAAGGAAAGCTGGATTATCAGCGCTTTGAATA<br>CGCTATAAAAAGTTTGGTAAATCGACATGAGGCGCTTCGAACGTCTTTCTATTCGC<br>TTAATGGAGAACCAGTTCAGCGTGTACATCAAAATGTAGAGCTACAGATTGCTTAT<br>TCGGAGGCGAAAGAAGATGAGATAGAGCAAATTGTAGAAAGCTTTGTTCAACCA<br>TTTGACCTTGAAATAGCTCCGCTGCTTCGCGTAGGGCTTGTTAAATTGGCATCGGA<br>TCGCTATTTATTCCTAATGGATATGCATCATATTATCTCAGATGGTGTATCAATGCAA<br>ATTATAACAAAAGAAATTGCCGACTTATATAAAGGAAAAGAGCTTGCTGAACTGC<br>ATATTCAGTATAAAGATTTTGCTGTATGGCAAAACGAATGGTTTCAATCTGACGCT

	CTTGAAAAACAGAAAACGTATTGGTTGAACACCTTTGCAGAGGATATTCCGGTTT<br>TAAATTTGTCAACTGATTATCCAAGACCGACAATTCAAAGTTTTGAAGGAGATATT<br>GTCACGTTTAGTGCAGGGAAGCAACTTGCGGAAGAATTGAAACGCCTGGCTGCA<br>GAAACAGGGACGACTTTGTATATGCTTCTGTTAGCGGCGTACAATGTACTTTTACA<br>CAAATACTCGGGACAGGAAGAAATTGTAGTAGGAACGCCTATTGCCGGGCGATCT<br>CACGCAGATGTGGAAAATATTGTTGGGATGTTTGTCAATACGCTTGCATTGAAAA<br>ATACCCCTATAGCCGTACGCACCTTCCACGAATTCCTGTTGGAAGTAAAACAAAA<br>TGCTTTAGAAGCTTTTGAAAATCAAGACTATCCATTTGAAAATTTGATAGAGAAGC<br>TGCAAGTGCGTCGCGACTTAAGTCGCAATCCATTATTTGATACAATGTTTAGCCTA<br>AGCAATATTGACGAACAAGTAGAGATAGGGATTGAGGGATTGAACTTCAGCCCAT<br>ATGAAATGCAGTATTGGATTGCAAAATTTGATATTTCATTCGATATTTTAGAAAAGC<br>AAGATGACATTCAATTTTATTTTAACTATTGCACGAATCTGTTTAAAAAAGAAACG<br>ATAGAACGATTAGCGACACACTTTATGCATATTTTACAGGAGATTGTTATTAATCCT<br>GAGATTAAGTTATGTGAAATTAATATGCTGTCCGAAGAAGAACAGCAGCGTGTCC<br>TGTATGACTTTAATGGCACAGATGCAACCTACGCTACGAATAAAATATTCCATGAG<br>TTATTTGAAGAACAGGTTGAAAAAACACCAGATCATATAGCGGTGATAGATGAAA<br>GAGAAAAGCTTTCCTATCAGGAGCTTAATGCGAAAGCGAATCAGCTGGCACGAG<br>TGCTGCGCCAAAAAGGAGTACAGCCTAATAGCATGGTAGGTATTATGGTAGATCG<br>CTCACTCGACATGATTGTAGGAATGCTTGGGGTTTTAAAAGCAGGAGGAGCATAT<br>GTGCCTATCGATATAGACTATCCTCAGGAACGGATTAGCTACATGATGGAAGATAG<br>TGGTGCAGCGCTCTTGTTAACACAACAAAAGTTGACACAGCAAATTGCGTTTTCT<br>GGTGACATTTTGTATCTTGACCAAGAAGAATGGCTTCATGAGGAAGCTTCAAATT<br>TAGAACCCATCGCTCGTCCGCAGGATATAGCCTATATCATTTACACTTCTGGTACA<br>ACCGGAAAGCCAAAAGGTGTGATGATTGAGCATCAAAGCTATGTGAATGTAGCA<br>ATGGCATGGAAAGATGCCTATCGGTTAGATACATTCCCGGTCCGTTTGCTTCAGAT<br>GGCTAGCTTTGCCTTTGACGTATCTGCGGGTGATTTTGCCAGAGCACTACTTACAG<br>GTGGGCAATTAATTGTATGTCCAAATGAAGTAAAGATGGACCCAGCTTCTTTATAT<br>GCCATTATTAAGAAATATGACATTACTATTTTTGAAGCAACGCCTGCTCTAGTGATT<br>CCATTGATGGAGTATATTTATGAACAGAAGCTGGATATTAGCCAGTTACAGATTCT<br>GATTGTCGGATCGGACAGTTGTTCGATGGAGGACTTTAAAACCTTGGTTTCCCGT<br>TTTGGTTCAACTATACGTATTGTGAATAGCTATGGAGTAACCGAAGCGTGCATTGA<br>TTCTAGCTATTATGAACAACCGCTTTCTTCGTTACATGTAACAGGAACTGTACCGA<br>TTGGAAAACCGTACGCTAACATGAAAATGTATATTATGAATCAATATTTGCAGATTC<br>AGCCTGTAGGTGTAATTGGAGAATTATGTATTGGAGGAGCCGGGGTTGCCCGTGG<br>ATATTTAAATAGACCGGACTTAACAGCAGAAAAGTTTGTCCCTAATCCTTTTGTTC<br>CAGGTGAAAAGCTGTATCGAACAGGCGACTTGGCAAGATGGATGCCGGATGGGA<br>ATGTTGAGTTTCTTGGTCGAAATGACCATCAGGTGAAAATCAGAGGGATTCGAAT<br>CGAGCTTGGAGAAATCGAAGCACAACTGCGTAAACATGATAGCATAAAAGAAGC<br>AACTGTGATCGCAAGAGAAGATCACATGAAAGAGAAATATTTATGTGCGTATATG<br>GTGACCGAAGGAGAAGTAAATGTAGCTGAACTGCGTGCGTATCTAGCAAATGATC<br>TGCCTGCGGCAATGATTCCGTCATATTTTGTATCGCTCGAAGCAATGCCACTTACT<br>GCTAATGGAAAAATTGATAAGCGATCTTTACCAGAGCCCGATGGTTCCATATCGAT<br>AGGAACAGAATATGTAGCTCCGCGTACCATGCTTGAGGGAAAACTAGAAGAGAT<br>ATGGAAAGATGTATTGGGTTTACAGCGTGTTGGCATTCACGATGACTTCTTTACAA<br>TAGGTGGCCATTCATTGAAGGCTATGGCTGTTATTTCGCAAGTTCATAAAGAATGC<br>CAGACTGAAGTTCCTCTGCGTGTCTTATTTGAAACACCTACCATTCAAGGACTGG<br>CTAAATATATAGAGGAAACGGACACAGAGCAATATATGGCTATTCAGCCGGTTAGC<br>GGACAGGACTATTATCCAGTATCATCAGCACAAAAGAGAATGTTTATTGTTAATCA<br>ATTTGATGGAGTAGGAATTAGCTACAATATGCCTTCCATCATGCTGATTGAAGGAA<br>AACTTGAGCGAACACGCTTGGAATCAGCATTTAAAAGATTGATAGAACGACATGA<br>GAGCCTTCGAACATCTTTTGAAATAATAAATGGTAAGCCTGTACAGAAGATTCATG<br>AGGAAGTTGATTTCAATATGTCCTATCAGGTGGCTTCTAATGAACAAGTAGAGAA<br>GATGATCGATGAGTTCATTCAGCCTTTCGATTTAAGTGTTGCACCGCTGCTTCGTG<br>TGGAACTTTTAAAATTGGAAGAGGACCGTCATGTGCTTATATTTGATATGCATCAT<br>ATTATCTCAGATGGTATATCTTCCAATATTTTGATGAAAGAATTAGGAGAACTATAT<br>CAAGGTAATGCTTTACCAGAACTTCGTATTCAATACAAGGATTTCGCTGTATGGCA<br>AAATGAGTGGTTCCAGTCAGAAGCCTTTAAAAAGCAAGAAGAATACTGGGTAAA

	TGTTTTCGCAGATGAACGCCCGATTCTGGATATACCGACGGATTATCCAAGGCCGA<br>TGCAACAAAGCTTTGATGGTGCTCAACTTACATTTGGAACCGGAAAGCAGCTTAT<br>GGATGGGTTATACAGGGTAGCAACGGAAACGGGAACAACGCTTTATATGGTTTTG<br>CTTGCGGCATATAATGTTCTTCTTTCCAAATATTCTGGTCAAGAAGATATTATTGTA<br>GGGACACCGATTGTGGGTAGATCCCATACTGACCTTGAGAATATTGTCGGGATGTT<br>TGTCAACACGTTAGCAATGAGAAATAAACCGGAAGGAGAAAAGACGTTCAAAGC<br>ATTTGTATCAGAAATAAAGCAGAATGCACTAGCGGCTTTTGAGAATCAGGATTATC<br>CATTTGAGGAGCTTATCGAAAAACTAGAGATACAAAGGGACTTAAGCAGAAATCC<br>ATTATTTGATACGCTCTTTAGCCTTCAAAACATAGGTGAAGAATCATTTGAACTAG<br>CCGAATTAACATGCAAACCTTTCGATTTGGTAAGCAAATTAGAGCATGCCAAGTT<br>TGATCTGAGTCTTGTGGCAGTAGAAAAAGAGGAAGAAATTGCATTTGGGCTTCA<br>ATACTGCACAAAACTGTATAAGGAAAAAACAGTTGAACAACTGGCTCAACATTTT<br>ATTCAAATAGTAAAAGCAATTGTAGAAAATCCAGATGTCAAATTATCTGATATTGA<br>TATGTTATCTGAAGAAGAGAAGAAACAAATCATGCTTGAGTTCAATGATACGAAA<br>ATACAATATCCGCAGAATCAAACAATACAGGAATTGTTTGAGGAGCAAGTGAAGA<br>AAACACCTGAACATATAGCAATCGTATGGGAAGGGCAAGCATTAACCTATCATGA<br>GCTAAATATAAAAGCTAATCAGTTAGCTCGTGTATTACGAGAAAAAGGGGTAACC<br>CCTAATCATCCTGTAGCGATTATGACGGAACGCTCATTAGAGATGATCGTAGGTAT<br>CTTTAGTATTTTGAAAGCAGGAGGAGCATATGTTCCAATTGATCCAGCCTATCCAC<br>AAGAACGTATTCAATACTTGCTTGAAGATAGCGGAGCGACGCTACTGCTTACTCA<br>GTCACATGTATTAAATAAATTACCGGTCGATATCGAATGGTTGGATCTTACAGATGA<br>ACAAAACTATGTAGAAGATGGTACCAATCTTCCATTTATGAATCAGTCAACAGATC<br>TTGCCTATATTATTTATACATCCGGTACAACAGGCAAGCCTAAAGGGGTTATGATTG<br>AACATCAAAGCATCATCAACTGCCTGCAATGGCGGA**T**GGAAGAATACGAATTTGG<br>ACCAGGGGATACGGCTCTACAAGTGTTTTCCTTTGCTTTTGATGGATTTGTAGCAA<br>GTTTGTTTGCTCCGATTCTTGCTGGTGCAACGTCTGTTCTCCCTAAGGAGGAAGA<br>AGCAAAAGATCCAGTTGCATTGAAAAAACTGATCGCATCAGAAGAGATTACACAT<br>TACTACGGTGTGCCTAGTTTGTTTAGTGCCATTCTTGATGTTTCTTCTAGTAAGGAT<br>TTGCAAAATTTACGCTGCGTCACTTTGGGAGGAGAGAAATTACCGGCTCAAATTG<br>TTAAAAAAATCAAAGAAAAAAATAAAGAAATTGAAGTCAACAACGAATATGGGC<br>CTACTGAAAATAGTGTAGTAACTACTATTATGCGCGATATACAGGTAGAACAAGAG<br>ATTACTATTGGTCGCCCATTATCTAACGTAGATGTATATATTGTCAATTGTAATCATC<br>AATTACAACCAGTAGGTGTAGTAGGGGAATTATGTATTGGTGGACAGGGACTTGC<br>AAGAGGATATTTGAATAAACCAGAGCTTACAGCAGATAAATTTGTTGTAAATCCAT<br>TCGTACCTGGTGAACGTATGTACAAAACCGGTGACCTTGCAAAATGGCGCTCAGA<br>TGGAATGATTGAATATGTGGGGCGTGTTGATGAACAAGTAAAAGTAAGAGGATAT<br>CGGATTGAGCTTGGTGAAATTGAATCAGCTATCCTAGAATACGAAAAAATTAAGG<br>AAGCGGTAGTTATGGTTTCGGAGCATACTGCATCTGAACAGATGTTATGTGCTTAT<br>ATTGTAGGGGAAGAAGATGTACTGACTCTGGACTTAAGAAGCTATCTAGCAAAAT<br>TACTACCAAGTTATATGATTCCAAACTATTTTATCCAATTGGATAGTATTCCGCTTAC<br>ACCAAACGGTAAAGTGGATCGTAAAGCATTGCCTGAACCTCAAACCATTGGCTTA<br>ATGGCAAGGGAGTATGTTGCACCAAGGAATGAAATCGAAGCACAGCTAGTACTC<br>ATTTGGCAAGAGGTATTAGGAATAGAACTGATCGGTATTACCGATAATTTCTTTGA<br>ATTAGGAGGGCATTCTTTAAAGGCAACGCTTTTAGTTGCAAAAATTTACGAGTAC<br>ATGCAAATAGAGATGCCATTAAATGTTGTGTTTAAACATTCAACTATTATGAAAATA<br>GCGGAATATATTACACATCAAGAATCAGAAAATAATGTACATCAGCCTATTTTGGT<br>AAATGTAGAAGCAGATAGAGAGGCGCTATCTCTTAACGGCGAGAAGCAAAGAAA<br>AAATATAGAGCTACCTATTCTGCTAAACGAAGAAACAGATCGAAACGTATTCTGCT<br>TCGCGCCCATTGGTGCACAAGGTGTTTTTTATAAAAAGCTTGCTGAACAAATCCC<br>TACTGCATCCTTGTATGGCTTTGACTTCATTGAAGATGATGATCGAATTCAGCAAT<br>ATATTGAATCGATGATTCAAACTCAGTCAGACGGACAATATGTGCTAATTGGTTAT<br>TCTTCAGGAGGGAACCTGGCTTTTGAAGTAGCAAAAGAAATGGAAAGGCAAGG<br>ATATAGTGTATCTGATTTGGTCTTGTTCGATGTTTACTGGAAGGGAAAAGTATTCG<br>AGCAAACAAAAGAAGAAGAAGAAGAAAACATAAAAATAATAATGGAAGAATTA<br>AGGGAAAATCCAGGAATGTTCAATATGACACGAGAGGATTTTGAACTGTATTTTG<br>CGAATGAATTTGTGAAACAAAGTTTCACACGGAAAATGCGCAAATACATGAGTTT<br>TTATACGCAGTTAGTTAATTATGGGGAAGTAGAAGCTACAATTCACCTTATACAAG

	CAGAATTTGAGGAAGAAAAAATTGACGAAAACGAAAAAGCCGACGAAGAAGAA AAAACATATCTAGAGGAAAAATGGAATGAAAAAGCATGGAACAAAGCAGCAAA AAGATTTGTAAAATATAACGGATATGGCGCTCATTCTAACATGCTAGGAGGTGATG GTTTAGAGAGAAATTCCTCTATCCTTAAACAGATACTACAAGGGACATTTGTAGTA AAATAAAAGAAGAAGTGTGAAAAAGCGCAGCTGAAATAGCTGCGCTTTTTTGTG TCATAA
pGETS151<br>PP1st-A8	AAAAGGCCTTCTTGGCCGCCCTTCCCGGTCGATATGAACAGCTTATTTACATAATT CACGTTATTGGTAGTTATAAATGAAATTCCTAATATCGGTTATGGAGTGAAATTGA ATTTCTACTTGATCTTTCTCTCTATTTTTGTAAAATAAAATTAAGAATATTTAAATAT TCAATGATTCATTTTTGCAGAAATCGGAGGAAGAAGAATATATGAAAACATTTAAC ATTTCTCAACAAGATCCCCCCATATTGTTGTATAAGTGATGAAATACCGAATTTAA AACCTAGTTTATATGTGGTAAAATGTTTTAATCAAGTTTAGGAGGAATTAATTATGA AGTGTAATGAATAATGAGTGTAACAGGGTTCAATTAAAAGAGGGAAGCGTATCAT TAACCCTATAAACTACGTCTGCCCTCATTATTGGAGGGTGAAATGTGAATACATCC TATTCACAATCGAATTTACGACACAACCAAATTTTAATTTGGCTTTGCATTTTATCT TTTTTTAGCGTATTAAATGAAATGGTTTTGAACGTGTCATTACCTGATATTGCAAAT GATTTTAATAAACCACCAGCGAGTACAAACTGGGTGAACACAGCCTTTATGTTAA CCTTTTCCATTGGAACAGCTGTATATGGAAAGCTATCTGATCAATTAGGCATCAAA AGGTTACTCCTATTTGGAATTATAATAAATTGTTTCGGGTCGGTAATTGGGTTTGTT GGCCATTCTTTCTTTTCCTTACTTATTATGGCTCGTTTTATTCAAGGGGCTGGTGCA GCTGCATTTCCAGCACTCGTAATGGTTGTAGTTGCGCGCTATATTCCAAAGGAAAA TAGGGGTAAAGCATTTGGTCTTATTGGATCGATAGTAGCCATGGGAGAAGGAGTC GGTCCAGCGATTGGTGGAATGATAGCCCATTATATTCATTGGTCCTATCTTCTACTC ATTCCTATGATAACAATTATCACTGTTCCGTTTCTTATGGAATTATTAAAGAAAGAA GTAAGGATAAAAGGTCATTTTGATATCAAAGGAATTATACTAATGTCTGTAGGCAT TGTATTTTTTATGTTGTTTACAACATCATATAGCATTTCTTTTCTTATCGTTAGCGTG CTGTCATTCCTGATATTTGTAAAACATATCAGGAAAGTAACAGATCCTTTTGTTGAT CCCGGATTAGGGAAAAATATACCTTTTATGATTGGAGTTCTTTGTGGGGGAATTAT ATTTGGAACAGTAGCAGGGTTTGTCTCTATGGTTCCTTATATGATGAAAGATGTTC ACCAGCTAGGTACTGCCGAAATCGGAAGTGTAATTATTTTCCCTGGAACAATGAG TGTCATTATTTTCGGCTACATTGGTGGGATACTTGTTGATAGAAGAGGTCCTTTATA CGTGTTAAACATCGGAGTTACATTTCTTTCTGTTAGCTTTTTAACTGCTTCCTTTCT TTTAGAAACAACATCATGGTTCATGACAATTATAATCGTATTTGTTTTAGGTGGGCT TTCGTTCACCAAAACAGTTATATCAACAATTGTTTCAAGTAGCTTGAAACAGCAG GAAGCTGGTGCTGGAATGAGTTTGCTTAACTTTACCAGCTTTTTATCAGAGGGAA CAGGTATTGCAATTGTAGGTGGTTTATTATCCATACCCTTACTTGATCAAAGGTTGT TACCTATGGAAGTTGATCAGTCAACTTATCTGTATAGTAATTTGTTATTACTTTTTTC AGGAATCATTGTCATTAGTTGGCTGGTTACCTTGAATGTATATAAACATTCTCAAA GGGATTTCTAAATCGTTAAGGGATCAACTTTGGGAGAGAGTTCAAAATTGATCCT TTTTTTATAACAGGAATTGGGCATCAAATAAAACGAAAGACTGGGCCTTTCGTTTT ATCTGTTGTTTGTCGGTGAACGCTCTCCTGAGTAGGACAGGTCCGCCGGGAGCG GATTTGAACGTTGCGAAGCAACGGCCCGGAGGGTGGCGGGCAGGACGCCCGCC ATAAACTGCCAGGCATCAAATTAAGCAGAAGGCCATCCTGACGGATGGCCTTTTT GCGTTTCTACAAACTCTTCCTGTCGTCATATCTACAATTCTACACAGCCCAGTCCA GACTATTGAATTGTATCACGGTTTTGATATCCTACCAATAACAAATTGATTGGAGG AATGCAAAGTGAATAATGAACCAGTAAAACGTGGTAAGAAGAACAGATGGGAAT TAAACCTACCTATAATGACTTATGTAGTAGCTGATGATTGGATTGATAAACTAGGAC ACGAAACGTTTACTTTATGGTTGAGGTTCCATACTTGGGTAGATAGAGAAGATGA ACTCCGAGATTATGATCGCATACCTAGAAGTTTTGAGAACATATATAAAAAGACAC TAGGAATCTCAAAAAGTAAGTTTTATAGATTGATAAAACCTTTATGGGAATATGGA TTAATAGACATCATAGAATACGAAGAGTCTAACCGTAATTCTACTAAACCTAAAAA TATAATTGTTTATGAGTATCCTTTACACGAAATAGAAAGAAAGTATAAACCACTAG AAAAATTAAGAGATTGGGATAAAGACTATAATTCCGTTTCTAAAGAATTAGGTAAA ACAGGTGGTAGACCAAGGAAAAAAGATAGTGAAGAAGAACCCGAAAAGAAACC CGAAGAAGTAACTAAAAAGAAACGTAAATATAAGTTAAAAAGAGTTATCCACAA CGGTTTCAAAAATGAAACGGTGGAGGGTTTCAAAAATGAAACGGTGGAGGGTTT CAAAAATGAAACGGTGACCGTTTCAAAAATAAAACCCAATAATTATTCAAATATCT

	TTAATAACTTATCAAATATTTCTACTAATGTTTCAAATAATTTATTAATTGATGATGA<br>TGAGGAAATCGAAAATGAACCAACTGGTCGTACAATAAATAGGTCATTACTTTTTT<br>CGCAAGAAGATATTAAACAGGCCTATCAATTTATTAATAGATTTTCAGTTATACAGT<br>TACGTGAAAACTTTAGCTTTGATAAACACTTTGAAGAACGGTTGGTATGTTATTTA<br>TGGAAAGCAGGGATTTCTACTTTTTAC**G**CGCACGAAATCAGTAAAATGATAAAAA<br>AAATAGCAGACTATGAAAAATCTAAAAAAGGTAGATTAAACCCAATACGTGACCG<br>AGCCTTATATATGGTAAATGGTCTTGTAATGAATAGAGCTTCTTCCCAAAGTGAAC<br>ATGCTACTTATAAACTAAACCAATATAAAAAACAGAAGGAACAGGAAAAACAAC<br>AACAGGAGCAACAAAGATCAAGAGTACCGTTCTATAATTGGTTGGAGGAAAGAG<br>AAGAACAAACCGAAGGTCAACTACCCACCACTTAAGCGGCCGCAAGCTTGAAG<br>AGCTCTTCTTTCAGAACGCTCGGTTGCCGCCGGGCGTTTTTTATGAGATGTCTCGG<br>CCTGTTTGGCCATTAATCGAAGAAGAAGTGTGAAAAAGCGCAGCTGAAATAGCT<br>GCGCTTTTTTGTGTCATAATCCTTCGATATATCGCG**C**CTATTCCGGCTTCCGGCTAT<br>CACCCGAAGATAAACAGCCCAGGGGTCACAGATGAAGTACTGAAGAAAATGAGG<br>AACGGTTTGATTAAGGTAAGGCCGTATACAGTCAATCGTCCGGAAGATATGAAGC<br>GTCTCATTGAAGCGGGAGCAGACGGCATGTTTACCGACTTTCCAGAAAAGGCTTC<br>GGCATTGCTGAAAAATGAATAGTTGTTAGAAGGAGGCTGTTTGACGCAGCCTTCT<br>TTTTTCATTCATTCATGC**T**CGTTTCAAAGCATACATTCATAGAAGACGGAAGAATA<br>AAGGGAGAGGTGAATG**A**GACTTTTATTTCACAAGTAAATAAATGGTTTGTTAATG<br>CTAATGTTAACTCAGCTGCAAAGCTTAGGCTATTCTGTATTCCATATGCAGGCGGT<br>GGTGCTTCCGCCTTTTATGAATGGAGTCATTTTTTTCCAAAGGAAATTGAAGTTTG<br>TTCAATTCAATTACCTGGAAGGGAAAATAGGGGGGCGGAAGTTCCGCTAACAAAT<br>TTACAACAGATAGTAGAAATAGTAGCTGAGGAAATACAACCATTAATAAATATTCC<br>ATTTGCTTTTTTGGGGCATAGCATGGGAGCATTAATAAGTTTTGAACTGGCTCGCA<br>CAATACGGCAAAAGAGTAATGTTAATCCGGTTCACTTGTTTGTTTCAGGGCGACA<br>TGCACCTCAAATCCCATGTGCAAAACAAGACTATCATTTACTTCCCGATGAACAAT<br>TTATACAAGAATTGCGTTCATTGAATGGAACTCCAGAG**G**TAGTATTACAAGACGC<br>AGAGATGATGAGTATATTACTCCCAAGACTTCGGGCTGATTTTTCTGTGTGTGGCT<br>CCTATCAGTACAAAAACGACGAGCCTTTTGAATGCCCAATCACTGCTTTTGGAGG<br>AAAAAATGATAATGGTGTTACTTATCAATCATTAGAAGCCTGGAGAGAGCAAACC<br>AAGAGGGAATTTTCTGTGTGTATGTATCCAGGTGATCATTTTTTTCTTTACGAAAG<br>CAAATATGAAATGATTGAGTTCATGTGTAAACAATTACGTTTAGTATTAGCTCCTAA<br>AATATAAGGCCTTGATGGCCATCGAAGAAGAAGTGTGAAAAAGCGCAGCTGAAA<br>TAGCTGCGCTTTTTTGTGTCATAATCCTTATTGAGTGGATGATTATAT**C**CCTTTTGAT<br>AGGTGGTATGTTTTCGCTTGAACTTTTAAATACAGCCATTGAACATACGGTTGATT<br>TAATAACTGACAAACATCACCCTCTTGCTAAAGCGGCCAAGGACGCTGCCGCCG<br>GGGCTGTTTGCGTTTTTGCCGTGATTTCGTGTATCATTGGTTTACTTATTTTTTTGC<br>CAAAGCTGTAATGGCTGAAAATTCTTACATTTATTTTACATTTTTAGAAATGGGCG<br>TGAAAAAAAGCGCGCGATTATGTAAAATATAAAGTGATAGCGGTACCGGA**G**CTAT<br>TTTTTACAGGGGGTATATATGTTAAACAG**C**TCTAAAAGTATATTGATTC**G**TGCTCAA<br>AATAAAAATGGAACGCATGAAGAGGAGCAGTATCTCTTTGCTGTGAACAACACC<br>AAAGCGGAGTATCCACGTGATAAGACGATCCATCAGTTATTTGAGGAGCAGGTTA<br>GTAAGAGGCCAAACAATGTAGCCATTGTATGTGAAAATGAGCAACTTACCTACCA<br>TGAGCTTAATGTGAAAGCCAATCAACTAGCACGGATTTTTATAGAAAAAGGGATT<br>GGAAAAGACACTCTTGTTGGAATTATGATGGAGAAATCTATCGATTTATTTATAGG<br>CATATTAGCCGTTTTAAAAGCTGGTGGAGCATATGTTCCGATTGATATTGAATATCC<br>TAAGGAAAGAATTCAATATATTCTTGATGATAGTCAGGCAAGAATGCTACTTACCC<br>AGAAGCATTTGGTTCATTTAATTCATAATATTCAATTTAATGGGCAAGTGGAAA**C**T<br>TTTGAAGAAGATACTATCAAAATTAGAGAAGGAACTAATCTACATGTACCAAGTA<br>AATCAACCGATCTTGCTTATGTTATTTATACTTCTGGTACAACAGGCAATCCAAAA<br>GGTACAATGCTGGAGCATAAAGGAATAAGTAATCTAAAGGTATTTTTCGAAAATA<br>GTCTTAACGTGACTGAAAAGGATAGAATTGGTCAATTTGCCAGCATCTCTTTTGAT<br>GCATCTGTATGGGAGATGTTTATGGCTTTGTTAACGGGGGCTAGCCTGTATATTATC<br>CTGAAGGATACAATCAATGATTT**C**GTGAAGTTTGAACAATACATTAACCAAAAGG<br>AAATCACTGTTATTACGTTACCACCTACCTATGTAGTTCATCTTGATCCAGAACGTA<br>TTTTATCGATACAAACGTTAATTACAGCAGGCTCAGCTACCTCGCCTTCCTTAGTA<br>AACAAG**A**GGAAGGAGAAAGTAACTTACATAAATGCCTATGGCCCTACGGAAACA

	ACTATTTGTGCGACTACATGGGTAGCCACCAAAGAAACAATAGGTCATTCAGTTC<br>CAATCGGAGCACCAATTCAAAATACACAAATTTATATTGTCGATGAAAATCTTCAA<br>TTAAAATCGGTTGGTGAAGCTGGTGAATTGTGTATTGGTGGAGAAGGGTTAGCAA<br>GGGGATATTGGAAGCGACCGGAATTAACTTCCCAGAAGTTCGTTGATAACCCGTT<br>TGTTCCAGGAGAGAAGTTGTATAAAACAGGAGATCAGGCAAGATGGCTATCTGAT<br>GGAAATATTGAATATCTCGGAAGAATAGATAACCAGGTAAAGATTAGAGGTCACC<br>GAGTTGAACTAGAAGAAGTTGAGTCTATTCTTCTAAAGCATATGTATATTAGCGAA<br>ACTGCAGTAAGTGTGCATAAAGATCACCAAGAACAGCCGTATTTGTGCGCTTATT<br>TTGTATCGGAAAAGCATATACCACTAGAAC**G**GTTAAGACAATTCTCATCAGAAGA<br>ACTGCCAACGTATATGATCCCTTCTTATTTTATCCAGTTAGACAAAATGCCGCTTAC<br>ATCAAATGGGAAGATTGATCGAAAGCAGTTGCCGGAACCTGATTTAACTTTCGGG<br>ATGAGGGTAGACTATGAAGCGCCGCGAAATGAAATCGAGGAAACGCTTGTTACTA<br>TCTGGCAGGATGTATTAGGTATTGAGAAAATCGGTATTAAAGATAATTTCTATGCAT<br>TAGGTGGAGATTCTATTAAAGCAATACAGGTTGCTGCTCGCCTGCATTCCTACCAA<br>TTAAAGCTAGAAACAAAAGATTTATTAAAGTATCCAACAATCGATCAACTCGTTC<br>ATTATATAAAAGATAGTAAAAGAAGAAGTGAGCAAGGTATTGTGGAAGGTGAGAT<br>TGGACTTACACCTATTCAGCATTGGTTCTTTGAACAACAATTTACAAATATGCACC<br>ATTGGAACCAATCGTATATGTTGTATAGACCAAATGGGTTTGATAAAGAGATCTTG<br>CTAAGGGTATTTAATAAAATTGTTGAGCATCATGATGCATTACGTATGATATACAAA<br>CATCATAACGGAAAGATCGTGCAGATAAATCGGGGGCTTGAAGGTACGTTGTTTG<br>ATTTTTATACCTTTGATTTAACTGCAAATGATAATGAGCAACAGGTGATTTGTGAA<br>GAATCTGCTCGATTACAAAATAGTATAAACTTGGAAGTAGGCCCTCTAGTAAAGAT<br>AGCGCTGTTTCATACTCAGAATGGAGATCACCTGTTTATGGCTATTCATCATTTGGT<br>TGTGGATGGTATTTCTTGGAGGATTTTGTTTGAGGATTTGGCCACAGCTTATGAAC<br>AAGCAATGCATCAGCAAACGATTGCTTTACCAGAGAAAACAGATTCATTTAAGGA<br>CTGGTCTATTGAATTAGAAAAATATGCGAACAGCGAATTATTCCTAGAAGAAGCT<br>GAATATTGGCATCATTTGAATTATTATACCGAGAACGTTCAAATTAAGAAAGATTAT<br>GTCACCATGAACAATAAACAAAAGAATATACGTTATGTAGGAATGGAGTTAACAA<br>TAGAAGAGACAGAAAAATTATTGAAAAATGTAAATAAAGCGTATCGAACAGAAAT<br>TAATGATATTTTATTAACGGCACTTGGCTTTGCACTCAAAGAATGGGCCGATATTG<br>ATAAAATTGTAATTAACTTAGAGGGACACGGACGGGAAGAAATACTGGAACAGAT<br>GAACATTGCAAGGACGGTAGGCTGGTTTACTTCCCAGTATCCTGTTGTACTTGATA<br>TGCAAAAATCGGATGATTTGTCTTATCAAATCAAATTAATGAAAGAAAATTTACGC<br>AGAATACCTAACAAAGGAATCGGATATGAAATTTTTAAGTATTTAACAACTGAATA<br>TTTACGGCCTGTTTTACCCTTTACATTAAAGCCGGAAATTAACTTTAACTACTTAG<br>GACAGTTCGATACGGACGTGAAAACTGAATTGTTTACTCGTTCTCCTTATAGCATG<br>GGTAATTCATTAGGACCAGATGGAAAAAATAATTTAAGCCCAGAAGGGGAAAGTT<br>ATTTTGTACTCAATATTAATGGTTTTATTGAAGAAGGTAAGCTTCACATCACCTTTT<br>CTTATAATGAACAGCAGTATAAGGAGGATACCATTCAGCAATTGAGCCGGAGCTAT<br>AAGCAACATCTTTTGGCCATCATTGAACATTGTGTACAGAAGGAAGATACTGAGT<br>TAACTCCAAGTGATTTCAGTTTCAAGGAACTTGAATTAGAAGAGATGGATGATATT<br>TTCGATTTGTTGGCCGATTCATTAACGTAAGGCCTCGATGGCCATCGAAGAAGAA<br>GTGTGAAAAAGCGCAGCTGAAATAGCTGCGCTTTTTTGTGTCATAATCCTTATGG<br>GAAGTGCTCCGTAATACGCTGACAAGAGAGAAAGGGCTTGGAGGTATTGAAACA<br>AGAGGAGTTCTGAGAATTGGTATGCCTTATAAGTCC**G**ATTAACAGTTGAAAACCT<br>GCATAGGAGAGCTATGCGGGTTTTTTATTTTACATAATGATACATAATTTACCGAAA<br>CTTGCGGAAC**C**TAATTGAGGAATCATAGAATTTTGTCAAAATAATTTTATTGACAA<br>CGTCTTATTAACGTTGATATAATTTAAATTTTATTTGACAAAAATGGGCTCGTGTTG<br>TACAATAAATGTAGTGGGAACATTATTATGAGGTGCTAGCATGAGTACATTTAAAA<br>AAGAACATGTTCAGGATATGTATCGTTTATCTCCCATGCAGGAAGGCATGTTGTTT<br>CACGCATTACTTGATAAAGATAAAAATGCTCACCTGGTACAAATGTCTATCGCGAT<br>CGAAGGTATCGTGGATGTGGAGCTGCTTAGTGAAAGCTTGAACATATTGATTGATA<br>GATACGATGTGTTTAGAACAACATTCTTACATGAAAAAATTAAACAACCGCTTCA<br>GGTAGTGCTAAAGGAACGGCCTGTTCAGCTTCAATTTAAAGACATATCATCCTTAG<br>ATGAAGAAAAAAGAGAACAGGCTATTGAGCAGTATAAGTATCAAGATGGGGAAA<br>CAGTCTTTGATTTAACAAGAGATCCCTTGATGAGAGTAGCTATTTTTCAAACTGGT<br>AAGGTTAACTACCAAATGATCTGGAGC**C**TCCACCATATTTTAATGGATGGTTGGTG

	CTTCAACATTATATTTAATGACTTGTTCAATATATATCTGTCATTAAAAGAGAAGAA<br>ACCTCTTCAGTTAGAGGCGGTGCAACCATATAAGCAGTTTATTAAGTGGCTTGAA<br>AAACAAGATAAACAGGAAGCACTTCGCTACTGGAAAGAACATTTAATGAATTATG<br>ATCAATCAGTAACATTACCTAAAAAGAAAGCAGCTATTAATAATACTACATATGAA<br>CCAGCACAGTTTCGTTTTGCGTTTGACAAAGTGCTTACCCAGCAGCTGCTTCGTA<br>TTGCCAATCAAAGCCAAGTAACACTAAATATTGTTTTTCAAACAATATGGGGGATT<br>GTACTTCAAAAATACAATTCCACTAATGATGTTGTATATGGCTCTGTTGTATCAGGC<br>CGTCCTTCTGAAATATCGGGAATTGAGAAAATGGTTGGACTATTTATTAATACTCTT<br>CCATTACGTATCCAAACGCAAAAAGATCAATCATTTATTGAATTAGTAAAGACTGT<br>TCATCAAA**G**CGTCCTTTTCTCGCAACAGCATGAGTATTTTCCATTGTATGAAATAC<br>AAAATCATACAGAATTAAAACAGAATCTGATTGATCATATTATGGTAATTGAAAAT<br>TATCCTTTAGTAGAAGAATTGCAAAAGAATAGTATCATGCAAAAAGTAGGGTTTAC<br>AGTTCGTGATGTCAAAATGTTTGAACCAACTAATTATGATATGACAGTTATGGTTT<br>TACCTCGTGATGAAATTAGTGTCCGACTCGATTATAACGCAGCCGTTTATGATATAG<br>ATTTCATAAAAAAAATTGAAGGTCACATGAAAGAAGTGGCTTTATGCGTGGCAAA<br>TAATCCACATGTGTTAGTACAGGACGTTCCTCTGCTTACAAAGCAAGAAAAACAA<br>CATTTATTGGTAGAGCTGCATGATTCGATAACAGAGTATCCTGATAAGACGATTCA<br>TCAGTTATTTACAG**G**ACAGGTAGAAAAAACACCAGAGCATGTGGCAGTTGTATTC<br>GAAGATGAGAAAGTGACCTATAGAGAGCTGCATGAGAGATCTAATCAATTAGCCA<br>GATTCTTAAGAGAAAAAGGCGTAAAAAAAGAAAGCATCATAGGCATTATGATGGA<br>GCGTTCAGTTGAAATGATTGTTGGGATCTTAGGGATTTTAAAAGCTGGTGGAGCT<br>TTTGTGCCTATTGATCCTGAATATCCAAAAGAAAGAATCGGCTATATGTTAGATTCT<br>GTACGGCTAGTACTTACACAACGCCATTTAAAGGATAAATTTGCTTTTACGAAAGA<br>AACGATAGTAATTGAAGATCCAAGTATTTCACACGAGTTAACTGAAGAAATAGAT<br>TATATTAATGAATCAGAGGACTTGTTTTATATTATTTATACATCAGGAACAACAGGT<br>AAACCAAAAGGGGTTATGCTAGAGCACAAAAACATCGTTAATCTGCTTCATTTTA<br>CTTTCGAGAAAACAAATATCAACTTTAGTGACAAAGTATTACAGTATACAACATGC<br>AGTTTTGACGTGTGTTACCAAGAAATTTTTTCGACGCTCTTGTCTGGAGGGCAAT<br>TATATCTTATTAGGAAAGAAACTCAACGCGATGTAGAGCAATTATTTGATTTAGTA<br>AAACGTGAAAATATTGAAGTATTATCCTTTCCTGTGGCTTTTCTAAAATTTATTTTC<br>AATGAAAGAGAATTTATCAATCGTTTTCCAACTTGCGTGAAACATATTATCACAGC<br>AGGAGAACAATTAGTAGTTAACAATGAGTTTAAACGTTATTTGCATGAACATAAC<br>GTACATTTACACAATCATTATGGTCCATCAGAAACGCATGTTGTTACCACCTATACT<br>ATTAATCCTGAAGCTGAAATTCCTGAATTACCACCGATAGGAAAACCTATCTCCAA<br>TACATGGATTTATATTTTGGATCAAGAACAACAACTACAACCACAAGGAATTGTAG<br>GAGAGTTATATATTTCGGGCGCAAATGTTGGAAGAGGATATTTGAATAATCAAGAA<br>TTAACGGCAGAAAAATTCTTTGCAGATCCCTTTAGGCCAAACGAACGGATGTACC<br>GAACAGGGGATTTAGCAAGGTGGTTGCCAGACGGAAATATCGAATTTTTAGGAA<br>GGGCCGATCATCAGGTGAAAATTAGGGGGCATCGAATAGAGCTTGGTGAGATCGA<br>GGCACAATTATTAAATTGTAAGGGTGTAAAAGAAGCTGTTGTTATCGATAAAGCG<br>GATGATAAAGGCGGAAAATATTTATGTGCCTATGTTGTTA**C**GGAAGTAGAAGTAA<br>ATGACTCTGAGCTTCGAGAATATTTGGGGAAAGCTTTGCCTGATTATATGATCCCG<br>TCGTTCTTTGTTCCGTTGGATCAGCTGCCGCTTACACCAAACGGAAAAATAGACA<br>G**G**AAATCTCTTCCGAATCTAGAGGGGATTGTGAATACAAACGCAAAATATGTAGT<br>ACCTACAAATGAGCTGGAAGAAAAATTGGCTAAAATCTGGGAAGAAGTACTTGG<br>GATTTCTCAGATCGGTATACAAGACAATTTCTTTTCGTTAGGCGGGCATTCTCTTA<br>AAGCCATTACGCTTATTTCCCGTATGAACAAAGAGTGTAATGTAGACATTCCTCTA<br>CGTTTGTTATTTGAAGCACCAACCATTCAGGAAATCTCTAATTATATAAACGGGGC<br>AAAGAAAGAAAGCTATGTTGCCATTCAGCCTGTACCAGAACAAGAGTACTATCCT<br>GTATCATCAGTTCAAAAAAGAATGTTTATTCTTAATGAATTTGATCGTTCAGGTAC<br>GGCCTATAATTTACCTGGTGTTATGTTTCTAGATGGAAAATTGAACTACCGACAAT<br>TGGAAGCAGCGGTAAAAAAATTAGTTGAGCGACATGAAGCGCTGCGTACTTCCT<br>TTCATTCAATTAATGGGGAACCAGTTCAGCGGGTGCATCAAAATGTAGAACTGCA<br>GATTGCTTATTCAGAGTCAACGGAAGATCAGGTGGAGCGAATTATTGCGGAATTT<br>ATGCAACCATTTGCTCTTGAAGTTGCTCCGTTACTTCGTGTAGGTCTTGTTAAATT<br>GGAGGCAGAACGTCATCTATTTATAATGGATATGCATCATATCATCTCGGATGGGGT<br>ATCCATGCAGATCATGATTCAAGAAATTGCTGATTTGTATAAAGAAAAGGAACTTC

<table>
<tr>
<td></td>
<td>CTACGTTAGGCATTCAATATAAAGACTTTACTGTTTGGCATAATCGCTTGCTTCAAT
CGGATGTTATTGAAAAACAAGAAGCTTACTGGCTGAACGTATTTGCAGAAGAGAT
TCCAGTATTGAATCTACCGACCGATTACCCAAGACCAACCATTCAAAGCTTTGATG
GTAAAAGATTTACATTCAGTACAGGAAAGCAGCTTATGGATGATTTATACAAGGTG
GCAACAGAAACAGGAACAACACTATATATGGTTTTACTCGCTGCGTATAATGTTTT
CTTATCGAAGTATTCCGGGCAAGATGACATCGTTGTAGGAACACCGATTGCTGGT
AGGTCCCATGCTGATGTGGAAAATATGCTGGGGATGTTTGTAAATACATTAGCAAT
AAGAAGTCGTTTAAATAATGAGGATACTTTTAAAGATTTTTTAGCAAATGTAAAAC
AAACGGCTTTGCATGCCTATGAAAATCCAGATTACCCATTTGATACGCTTGTCGAA
AAGTTGGGTATACAGAGAGATTTAAGTAGAAATCCATTATTTGATACGATGTTTGT
TTTGCAAAATACGGATAGAAAGTCTTTTGAGGTTGAACAGATAACGATTACACCA
TATGTTCCAAATAGCAGACATCCTAAATTTGATCTTACATTAGAGGTTAGCGAAGA
ACAAAATGAGATTTTATTATGCCTAGAATATTGCACTAAATTATTTACGGATAAAAC
AGTTGAAAGAATGGCTGGTCATTTTTTACAGATCTTGCATGCAATTGTTGGGAACC
CAACGATTATAATATCAGAAATCGAGATATTGTCTGAAGAAGAAAAACAACATATT
TTATTCGAGTTCAACGATACGAAAACCACATATCCACATATGCAAACAATTCAAGG
ATTATTTGAGGAACAGGTGGAGAAAACGCCCGACCATGTTGCAGTTGGATGGAA
AGACCAAACATTAACGTATCGGGAACTTAACGAAAGAGCGAATCAGGTCGCAAG
AGTCTTACGGCAAAAAGGAGTCCAACCCGATAATATCGTGGGATTGCTGGTTGAG
CGTTCACCTGAAATGCTCGTGGGTATCATGGGAATTCTTAAAGCAGGGGGAGCTT
ATTTACCTCTTGATCCGGAGTACCCAGCGGATAGAATTTCGTACATGATACAAGAT
TGTGGTGTACGCATTATGCTTACCCAACAGCATCTTTTATCTTTAGTACATGATGAA
TTTGATTGTGTTATTTTGGATGAGGACAGTTTGTACAAGGGGGATTCTTCCAATTT
GGCTCCGGTTAACCAGGCCGGGGATGTAGCCTACATCATGTACACTTCTGGTTCT
ACAGGAAAGCCTAAAGGTGTTATGGTAGAACATCGAAATGTGATTCGCCTTGTGA
AAAATACAAATTATGTTCAGGTCCGCGAGGACGATCGTATAATACAGACCGGAGC
AATTGGATTCGATGCACTGACATTTGAAGTTTTTGGCTCATTGCTGCATGGAGCTG
AATTGTATCCTGTTACTAAAGACGTGCTATTAGATGCAGAGAAACTACACAAATTT
TTACAAGCGAATCAAATTACGATTATGTGGTTAACTTCTCCGTTATTTAACCAATTG
TCACAAGGAACCGAAGAGATGTTTGCTGGCCTTCGCTCCCTAATTGTAGGTGGAG
ATGCCTTGTCTCCGAAACACATCAATAATGTAAAGCGAAAATGCCCTAATCTGACT
ATGTGGAACGGTTACGGCCCAACAGAAAACACCACTTTTTCTACATGCTTTCTTAT
TGATAAAGAATATGATGACAATATTCCGATAGGGAAGGCCATTAGTAATTCAACAG
TGTATATCATGGACCGGTATGGCCAGCTTCAGCCGGTGGGTGTACCAGGAGAATT
ATGTGTAGGAGGGGATGGGGTTGCCAGGGAATATATGAATCAGCCTGCATTAACA
GAAGAGAAGTTTGTCCCAAATCCATTCGCTCCTGGTGAGAGAATGTATCGCACGG
GGGATTTGGCAAGATGGTTGCCTGATGGAACAATTGAGTATTTAGGTCGTATTGAT
CAGCAAGTGAAAATCAGGGGCTACCGTATTGAACCGGGAGAGATTGAAACGCTT
CTTGTGAAGCACAAAAAAGTCAAAGAATCGGTAATCATGGTAGTAGAGGATAATA
ATGGACAAAAGGCTCTATGCGCTTATTACGTTCCGGAAGAAGAAGTAACGGTATC
TGAACTGAGGGAATATATAGCTAAAGAGTTGCCTGTTTACATGGTTCCAGCCTATT
TTGTACAGATTGAACAAATGCCTCTTACACAGAACGGTAAAGTAAATCGAAGCGC
GTTACCAAAACCAGATGGTGAATTTGGTACAGCAACCGAATATGTAGCGCCTAGC
AGCGACATTGAAATGAAGCTGGCAGAGATTTGGCATAATGTGTTAGGGGTAAACA
AAATCGGGGTACTGGATAACTTCTTTGAATTAGGTGGTCATTCATTAAGAGCTATG
ACAATGATTTCCCAGGTACATAAAGAGTTCGACGTTGAATTGCCATTAAAAGTGT
TATTTGAAACACCAGCGATCTCTGCATTAGCTCAATACATTGCTGATGGAGAAAA
AGGAATGTACCTGGCCATTCAACCTGTTACCCCGCAGGATTACTATCCAGTATCAT
CTGCGCAAAAGAGGATGTACATCCTTTATGAATTTGAAGGGGCTGGCATTACCTAT
AATGTACCTAATGTAATGTTTATAGAAGGAAAGCTGGATTATCAGCGCTTTGAATA
CGCTATAAAAAGTTTGGTAAATCGACATGAGGCGCTTCGAACGTCTTTCTATTCGC
TTAATGGAGAACCAGTTCAGCGTGTACATCAAAATGTAGAGCTACAGATTGCTTAT
TCGGAGGCGAAAGAAGATGAGATAGAGCAAATTGTAGAAAGCTTTGTTCAACCA
TTTGACCTTGAAATAGCTCCGCTGCTTCGCGTAGGGCTTGTTAAATTGGCATCGGA
TCGCTATTTATTCCTAATGGATATGCATCATATTATCTCAGATGGTGTATCAATGCAA
ATTATAACAAAAGAAATTGCCGACTTATATAAAGGAAAAGAGCTTGCTGAACTGC
ATATTCAGTATAAAGATTTTGCTGTATGGCAAAACGAATGGTTTCAATCTGACGCT</td>
</tr>
</table>

	CTTGAAAAACAGAAAACGTATTGGTTGAACACCTTTGCAGAGGATATTCCGGTTT<br>TAAATTTGTCAACTGATTATCCAAGACCGACAATTCAAAGTTTTGAAGGAGATATT<br>GTCACGTTTAGTGCAGGGAAGCAACTTGCGGAAGAATTGAAACGCCTGGCTGCA<br>GAAACAGGGACGACTTTGTATATGCTTCTGTTAGCGGCGTACAATGTACTTTTACA<br>CAAATACTCGGGACAGGAAGAAATTGTAGTAGGAACGCCTATTGCCGGGCGATCT<br>CACGCAGATGTGGAAAATATTGTTGGGATGTTTGTCAATACGCTTGCATTGAAAA<br>ATACCCCTATAGCCGTACGCACCTTCCACGAATTCCTGTTGGAAGTAAAACAAAA<br>TGCTTTAGAAGCTTTTGAAAATCAAGACTATCCATTTGAAAATTTGATAGAGAAGC<br>TGCAAGTGCGTCGCGACTTAAGTCGCAATCCATTATTTGATACAATGTTTAGCCTA<br>AGCAATATTGACGAACAAGTAGAGATAGGGATTGAGGGATTGAACTTCAGCCCAT<br>ATGAAATGCAGTATTGGATTGCAAAATTTGATATTTCATTCGATATTTTAGAAAAGC<br>AAGATGACATTCAATTTTATTTTAACTATTGCACGAATCTGTTTAAAAAAGAAACG<br>ATAGAACGATTAGCGACACACTTTATGCATATTTTACAGGAGATTGTTATTAATCCT<br>GAGATTAAGTTATGTGAAATTAATATGCTGTCCGAAGAAGAACAGCAGCGTGTCC<br>TGTATGACTTTAATGGCACAGATGCAACCTACGCTACGAATAAAATATTCCATGAG<br>TTATTTGAAGAACAGGTTGAAAAAACACCAGATCATATAGCGGTGATAGATGAAA<br>GAGAAAAGCTTTCCTATCAGGAGCTTAATGCGAAAGCGAATCAGCTGGCACGAG<br>TGCTGCGCCAAAAAGGAGTACAGCCTAATAGCATGGTAGGTATTATGGTAGATCG<br>CTCACTCGACATGATTGTAGGAATGCTTGGGGTTTTAAAAGCAGGAGGAGCATAT<br>GTGCCTATCGATATAGACTATCCTCAGGAACGGATTAGCTACATGATGGAAGATAG<br>TGGTGCAGCGCTCTTGTTAACACAACAAAAGTTGACACAGCAAATTGCGTTTTCT<br>GGTGACATTTTGTATCTTGACCAAGAAGAATGGCTTCATGAGGAAGCTTCAAATT<br>TAGAACCCATCGCTCGTCCGCAGGATATAGCCTATATCATTTACACTTCTGGTACA<br>ACCGGAAAGCCAAAAGGTGTGATGATTGAGCATCAAAGCTATGTGAATGTAGCA<br>ATGGCATGGAAAGATGCCTATCGGTTAGATACATTCCCGGTCCGTTTGCTTCAGAT<br>GGCTAGCTTTGCCTTTGACGTATCTGCGGGTGATTTTGCCAGAGCACTACTTACAG<br>GTGGGCAATTAATTGTATGTCCAAATGAAGTAAAGATGGACCCAGCTTCTTTATAT<br>GCCATTATTAAGAAATATGACATTACTATTTTTGAAGCAACGCCTGCTCTAGTGATT<br>CCATTGATGGAGTATATTTATGAACAGAAGCTGGATATTAGCCAGTTACAGATTCT<br>GATTGTCGGATCGGACAGTTGTTCGATGGAGGACTTTAAAACCTTGGTTTCCCGT<br>TTTGGTTCAACTATACGTATTGTGAATAGCTATGGAGTAACCGAAGCGTGCATTGA<br>TTCTAGCTATTATGAACAACCGCTTTCTTCGTTACATGTAACAGGAACTGTACCGA<br>TTGGAAAACCGTACGCTAACATGAAAATGTATATTATGAATCAATATTTGCAGATTC<br>AGCCTGTAGGTGTAATTGGAGAATTATGTATTGGAGGAGCCGGGGTTGCCCGTGG<br>ATATTTAAATAGACCGGACTTAACAGCAGAAAAGTTTGTCCCTAATCCTTTTGTTC<br>CAGGTGAAAAGCTGTATCGAACAGGCGACTTGGCAAGATGGATGCCGGATGGGA<br>ATGTTGAGTTTCTTGGTCGAAATGACCATCAGGTGAAAATCAGAGGGATTCGAAT<br>CGAGCTTGGAGAAATCGAAGCACAACTGCGTAAACATGATAGCATAAAAGAAGC<br>AACTGTGATCGCAAGAGAAGATCACATGAAAGAGAAATATTTATGTGCGTATATG<br>GTGACCGAAGGAGAAGTAAATGTAGCTGAACTGCGTGCGTATCTAGCAAATGATC<br>TGCCTGCGGCAATGATTCCGTCATATTTTGTATCGCTCGAAGCAATGCCACTTACT<br>GCTAATGGAAAAATTGATAAGCGATCTTTACCAGAGCCCGATGGTTCCATATCGAT<br>AGGAACAGAATATGTAGCTCCGCGTACCATGCTTGAGGGAAAACTAGAAGAGAT<br>ATGGAAAGATGTATTGGGTTTACAGCGTGTTGGCATTCACGATGACTTCTTTACAA<br>TAGGTGGCCATTCATTGAAGGCTATGGCTGTTATTTCGCAAGTTCATAAAGAATGC<br>CAGACTGAAGTTCCTCTGCGTGTCTTATTTGAA**G**CACCTACCATTCAAGGACTGG<br>CTAAATATATAGAGGAAACGGACACAGAGCAATATATGGCTATTCAGCCGGTTAGC<br>GGACAGGACTATTATCCAGTATCATCAGCACAAAAGAGAATGTTTATTGTTAATCA<br>ATTTGATGGAGTAGGAATTAGCTACAATATGCCTTCCATCATGCTGATTGAAGGAA<br>AACTTGAGCGAACACGCTTGGAATCAGCATTTAAAAGATTGATAGAACGACATGA<br>GAGCCTTCGAACATCTTTTGAAATAATAAATGGTAAGCCTGTACAGAAGATTCATG<br>AGGAAGTTGATTTCAATATGTCCTATCAGGTGGCTTCTAATGAACAAGTAGAGAA<br>GATGATCGATGAGTTCATTCAGCCTTTCGATTTAAGTGTTGCACCGCTGCTTCGTG<br>TGGAACTTTTAAAATTGGAAGAGGACCGTCATGTGCTTATATTTGATATGCATCAT<br>ATTATCTCAGATGGTATATCTTCCAATATTTTGATGAAAGAATTAGGAGAACTATAT<br>CAAGGTAATGCTTTACCAGAACTTCGTATTCAATACAAGGATTTCGCTGTATGGCA<br>AAATGAGTGGTTCCAGTCAGAAGCCTTTAAAAAGCAAGAAGAATACTGGGTAAA

	TGTTTTCGCAGATGAACGCCCGATTCTGGATATACCGACGGATTATCCAAGGCCGA<br>TGCAACAAAGCTTTGATGGTGCTCAACTTACATTTGGAACCGGAAAGCAGCTTAT<br>GGATGGGTTATACAGGGTAGCAACGGAAACGGGAACAACGCTTTATATGGTTTTG<br>CTTGCGGCATATAATGTTCTTCTTTCCAAATATTCTGGTCAAGAAGATATTATTGTA<br>GGGACACCGATTGTGGGTAGATCCCATACTGACCTTGAGAATATTGTCGGGATGTT<br>TGTCAACACGTTAGCAATGAGAAATAAACCGGAAGGAGAAAAGACGTTCAAAGC<br>ATTTGTATCAGAAATAAAGCAGAATGCACTAGCGGCTTTTGAGAATCAGGATTATC<br>CATTTGAGGAGCTTATCGAAAAACTAGAGATACAAAGGGACTTAAGCAGAAATCC<br>ATTATTTGATACGCTCTTTAGCCTTCAAAACATAGGTGAAGAATCATTTGAACTAG<br>CCGAATTAACATGCAAACCTTTCGATTTGGTAAGCAAATTAGAGCATGCCAAGTT<br>TGATCTGAGTCTTGTGGCAGTAGAAAAAGAGGAAGAAATTGCATTTGGGCTTCA<br>ATACTGCACAAAACTGTATAAGGAAAAAACAGTTGAACAACTGGCTCAACATTTT<br>ATTCAAATAGTAAAAGCAATTGTAGAAAATCCAGATGTCAAATTATCTGATATTGA<br>TATGTTATCTGAAGAAGAGAAGAA**G**CAAATCATGCTTGAGTTCAATGATACGAAA<br>ATACAATATCCGCAGAATCAAACAATACAGGAATTGTTTGAGGAGCAAGTGAAGA<br>AAACACCTGAACATATAGCAATCGTATGGGAAGGGCAAGCATTAACCTATCATGA<br>GCTAAATATAAAAGCTAATCAGTTAGCTCGTGTATTACGAGAAAAAGGGGTAACC<br>CCTAATCATCCTGTAGCGATTATGACGGAACGCTCATTAGAGATGATCGTAGGTAT<br>CTTTAGTATTTTGAAAGCAGGAGGAGCATATGTTCCAATTGATCCAGCCTATCCAC<br>AAGAACGTATTCAATACTTGCTTGAAGATAGCGGAGCGACGCTACTGCTTACTCA<br>GTCACATGTATTAAATAAATTACCGGTCGATATCGAATGGTTGGATCTTACAGATGA<br>ACAAAACTATGTAGAAGATGGTACCAATCTTCCATTTATGAATCAGTCAACAGATC<br>TTGCCTATATTATTTATACATCCGGTACAACAGGCAAGCCTAAAGGGGTTATGATTG<br>AACATCAAAGCA**C**CATCAACTGCCTGCAATGGCGGAAGGAAGAATACGAATTTG<br>GACCAGGGGATACGGCTCTACAAGTGTTTTCCTTTGCTTTTGATGGATTTGTAGCA<br>AGTTTGTTTGCTCCGATTCTTGCTGGTGCAACGTCTGTTCTCCCTAAGGAGGAAG<br>AAGCAAAAGATCCAGTTGCATTGAAAAAACTGATCGCATCAGAAGAGATTACAC<br>ATTACTACGGTGTGCCTAGTTTGTTTAGTG**T**CATTCTTGATGTTTCTTCTAGTAAGG<br>ATTTGCAAAATTTACGCTGCGTCACTTTGGGAGGAGAGAAATTACCGGCTCAAAT<br>TGTTAAAAAAATCAAAGAAAAAAATAAAGAAATTGAAGTCAACAACGAATATGG<br>GCCTACTGAAAATAGTGTAGTAACTACTATTATGCGCGATATACAGGTAGAACAAG<br>AGATTACTATTGGTCGCCCATTATCTAACGTAGATGTATATATTGTCAATTGTAATCA<br>TCAATTACAACCAGTA**A**GTGTAGTAGGGGAATTATGTATTGGTGGACAGGGACTT<br>GCAAGAGGATATTTGAATAAACCAGAGCTTACAGCAGATAAATTTGTTGTAAATC<br>CATTCGTACCTGGTGAACGTATGTACAAAACCGGTGACCTTGCAAAATGGCGCTC<br>AGATGGAATGATTGAATATGTGGGGCGTGTTGATGAACAAGTAAAAGTAAGAGGA<br>TATCGGATTGAGCTTGGTGAAATTGAATCAGCTATCCTAGAATACGAAAAAATTAA<br>GGAAGCGGTAGTTATGGTTTCGGAGCATACTGCATCTGAACAGATGTTATGTGCTT<br>ATATTGTAGGGGAAGAAGATGTACTGACTCTGGACTTAAGAAGCTATCTAGCAAA<br>ATTACTACCAAGTTATATGATTCCAAACTATTTTATCCAATTGGATAGTATTCCGCTT<br>ACACCAAACGGTAAAGTGGATCGTAAAGCATTGCCTGAACCTCAAACCATTGGCT<br>TAATGGCAAGGGAGTATGTTGCACCAAGGAATGAAATCGAAGCACAGCTAGTACT<br>CATTTGGCAAGAGGTATTAGGAATAGAACTGATCGGTATTACCGATAATTTCTTTG<br>AATTAGGAGGGCATTCTTTAAAGGCAACGCTTTTAGTTGCAAAAATTTACGAGTA<br>CATGCAAATAGAGATGCCATTAAATGTTGTGTTTAAACATTCAACTATTATGAAAA<br>TAGCGGAATATATTACACATCAAGAATCAGAAAATAATGTACATCAGCCTATTTTG<br>GTAAATGTAGAAGCAGATAGAGAGGCGCTATCTCTTAACGGCGAGAAGCAAAGA<br>AAAAATATAGAGCTACCTATTCTGCTAAACGAAGAAACAGATCGAAACGTATTCT<br>GCTTCGCGCCCATTGGTGCACAAGGTGTTTTTTATAAAAAGCTTGCTGAACAAAT<br>CCCTACTGCATCCTTGTATGGCTTTGACTTCATTGAAGATGATGATCGAATTCAGC<br>A**G**TATATTGAATCGATGATTCAAACTCAGTCAGACGGACAATATGTGCTAATTGGT<br>TATTCTTCAGGAGGGAACCTGGCTTTTGAAGTAGCAAAAGAAATGGAAAGGCAA<br>GGATATAGTGTATCTGATTTGGTCTTGTTCGATGTTTACTGGAAGGGAAAAGTATT<br>CGAGCAAACAAAAGAAGAAGAAGAAGAAAACATAAAAATAATAATGGAAGAAT<br>TAAGGGAAAATCCAGGAATGTTCAATATGACACGAGAGGATTTTGAACTGTATTT<br>TGCGAATGAATTTGTGAAACAAAGTTTCACACGGAAAATGCGCAAATACATGAGT<br>TTTTATACGCAGTTAGTTAATTATGGGGAAGTAGAAGCTACAATTCACCTTATACA

	AGCAGAATTTGAGGAAGAAAAAATTGACGAAAACGAAAAAGCCGACGAAGAAG AAAAAACATATCTAGAGGAAAAATGGAATGAAAAAGCATGGAACAAAGCAGCA AAAAGATTTGTAAAATATAACGGATATGGCGCTCATTCTAACATGCTAGGAGGTGA TGGTTTAGAGAGAAATTCCTCTATCCTTAAACAGATACTACAAGGGACATTTGTAG TAAAATAAAAGAAGAAGTGTGAAAAAGCGCAGCTGAAATAGCTGCGCTTTTTTG TGTCATAA

Table S2. Nucleotide and amino acid substitutions in pGETS151 P2nd-B2 and pGETS151 PP1st-A8.

Plasmid	Region [a]	Nucleotide Substitution [a]	Amino Acid Substitution [b]
pGETS151 P2nd-B2	Vector (1-3,477)	T-274-C	-
		G-1,247-A	G-270-E (TcR)
		A-1,955-G	-
		T-3,077-C	-
	grsA (5,036-8,332)		
	^{D}Phe-A domain (5,168-6,343)	T-5,972-A	W-313-R
	^{D}Phe-PCP domain (6,665-6,868)	Non	-
	^{D}Phe-E domain (6,896-7,801)	Non	-
	Non-domain region	T-5,047-C	Synonymous
		A-5,067-G	H-11-R
	grsB (8,732-22,084)		
	^{L}Pro-C domain (8,759-9,658)	Non	-
	^{L}Pro-A domain (10,151-11,338)	Non	-
	^{L}Pro-PCP domain (11,660-11,860)	Non	-
	^{L}Val-C domain (11,909-12,769)	Non	-
	^{L}Val-A domain (13,265-14,443)	G-13,473-A	G-1,581-E
		T-13,548-C	I-1,606-T
	^{L}Val-PCP domain (14,765-14,971)	A-14,905-G	Synonymous
	^{L}Orn-C domain (15,014-15,868)	Non	-
	^{L}Orn-A domain (16,373-17,578)	Non	-
	^{L}Orn-PCP domain (17,900-18,106)	Non	-
	^{L}Leu-C domain (18,149-19,009)	Non	-
	^{L}Leu-A domain (19,511-20,692)	A-20,013-T	K-3,761-M
	^{L}Leu-PCP domain (21,014-21,220)	Non	-
	TE domain (21,350-22,063)	Non	-
	Non-domain region	A-11,590-G	Synonymous
pGETS151 PP1st-A8	Vector (1-3,477)	A-101-G	-
		T-274-C	-
		A-997-G	K-187-E (TcR)
		A-1,955-G	-
		A-2,494-G	-
		A-3,107-G	-
	P_{mmgA} (3,540-3,839)	T-3,553-C	-
		C-3,811-T	-
	grsT (3,864-4,634)	T-3,865-A	V-1-E
		A-4,332-G	I-157-V
	P_{cdd} (4,710-5,009)	T-4,730-C	-
		A-5,013-G	-
	grsA (5,036-8,332)		
	^{D}Phe-A domain (5,168-6,343)	T-5,517-C	I-161-T
		T-5,821-C	Synonymous
		T-5,972-A	W-313-R
	^{D}Phe-PCP domain (6,665-6,868)	Non	-
	^{D}Phe-E domain (6,896-7,801)	Non	-
	Non-domain region	T-5,047-C	Synonymous
		A-5,067-G	H-11-R
		A-6,492-G	Q-486-R
	P_{veg} (8,408-8,707)	A-8,503-G	-
		A-8,589-C	-
	grsB (8,732-22,084)		
	^{L}Pro-C domain (8,759-9,658)	T-9,164-C	F-145-L
	^{L}Pro-A domain (10,151-11,338)	A-10,158-G	E-476-G

	^{L}Pro-PCP domain (11,660-11,860)	Non	-
	^{L}Val-C domain (11,909-12,769)	T-12,628-C	Synonymous
	^{L}Val-A domain (13,265-14,443)	T-13,673-G	L-1,648-V
		G-14,289-A	G-1,853-E
	^{L}Val-PCP domain (14,765-14,971)	A-14,936-G	T-2,069-A
	^{L}Orn-C domain (15,014-15,868)	Non	-
	^{L}Orn-A domain (16,373-17,578)	Non	-
	^{L}Orn-PCP domain (17,900-18,106)	A-18,065-G	T-3,112-A
	^{L}Leu-C domain (18,149-19,009)	Non	-
	^{L}Leu-A domain (19,511-20,692)	T-19,989-C	I-3,753-T
		C-20,226-T	A-3,832-V
		G-20,492-A	G-3,921-S
	^{L}Leu-PCP domain (21,014-21,220)	Non	-
	TE domain (21,350-22,063)	A-21,475-G	Synonymous
	Non-domain region	A-9,705-G	N-325-S
		T-11,463-C	M-911-T
		A-11,590-G	Synonymous
		T-13,001-C	S-1,424-P
		A-19,444-G	Synonymous

[a] The number represents the position of the nucleotide in the plasmid sequences in Table S1.
[b] The number represents the position of the amino acid in the corresponding gene.

Table S3. List of primers used for epPCR in this study. The bold and underlined sequence is the AarI-recognition sequence.

Primer Name	Sequence (5′ to 3′)
Fragment 1-F	TAG**CACCTGC**ATGCAAGCGTATCATTAACCCTATAAACTACGT
Fragment 1-R	CTA**CACCTGC**GCATACCTATTGTTTCTTTGGTGGCTAC
Fragment 2-F	TAG**CACCTGC**ATGCAGGTCATTCAGTTCCAATCGGA
Fragment 2-R	CTA**CACCTGC**GCATTTCCCCAAATATTCTCGAAGCTCA
Fragment 3-F	TAG**CACCTGC**ATGCGGAAAGCTTTGCCTGATTATATGATC
Fragment 3-R	CTA**CACCTGC**GCATATGGCATATAAAGAAGCTGGGTC
Fragment 4-F	TAG**CACCTGC**ATGCCCATTATTAAGAAATATGACATTACTATTTTTGAAGCAAC
Fragment 4-R	CTA**CACCTGC**GCATGCTTCCCTCTTTTAATTGAACCCT
